# Review of the Genus *Sycanus* Amyot & Serville, 1843 (Heteroptera: Reduviidae: Harpactorinae), from China Based on DNA Barcoding and Morphological Evidence †

**DOI:** 10.3390/insects15030165

**Published:** 2024-02-28

**Authors:** Ping Zhao, Suyi Chen, Yingqi Liu, Jianyun Wang, Zhuo Chen, Hu Li, Wanzhi Cai

**Affiliations:** 1 Key Laboratory of Environment Change and Resources Use in Beibu Gulf (Ministry of Education) and Guangxi Key Laboratory of Earth Surface Processes and Intelligent Simulation, Nanning Normal University, Nanning 530001, China; zpyayjl@126.com; 2 Department of Entomology and MOA Key Lab of Pest Monitoring and Green Management, College of Plant Protection, China Agricultural University, Beijing 100193, China; chensuyiii@126.com (S.C.); yingqiliu0720@163.com (Y.L.); insectchen625@126.com (Z.C.); tigerleecau@hotmail.com (H.L.); 3 Sanya Institute of China Agricultural University, Sanya 572025, China; 4 Environment and Plant Protection Institute, Chinese Academy of Tropical Agricultural Sciences, Haikou 571101, China; wjy-1989@163.com

**Keywords:** China, *Sycanus*, taxonomy, DNA barcoding, species delimitation, phylogeny, new species, biology

## Abstract

**Simple Summary:**

The reduviid genus *Sycanus* Amyot & Serville, 1843, possesses higher aesthetic value and plays a crucial role as a natural enemy in the control of agricultural and forestry pests. However, *Sycanus* from China has not undergone a comprehensive review for over four decades. Based on both molecular data and morphological evidence, we conducted a systematic review of the 14 Chinese *Sycanus* species, including a description of three new species.

**Abstract:**

Due to the variability of body coloration and morphological similarity among closely related species, unresolved issues and debates still persist in the taxonomic study of the genus *Sycanus* from China. In this study, we conducted phylogenetic analyses and species delimitation for *Sycanus* in China based on a COI DNA barcoding dataset comprising 81 samples. The results revealed that all the samples could be classified into 12 species by integrating molecular analyses with morphological comparison. This paper provides a comprehensive systematic review of the *Sycanus* species found in China, including descriptions of three new species: *S. taiwanensis* Zhao & Cai sp. nov., *S. flavicorius* Li & Cai sp. nov., and *S. hainanensis* Wang & Cai sp. nov. Furthermore, it is proposed that *S. croceovittatus* Dohrn, 1859, *S. leucomesus* Walker, 1873, and *S. villicus* Stål, 1863, are three synonyms of *S. bifidus* (Fabricius, 1787); *S. bicolor* Hsiao, 1979, is a synonym of *S. versicolor* Dohrn, 1859; and *S. hsiaoi* Maldonado-Capriles, 1990, is a synonym of *S. marginellus* Putshkov, 1987. Additionally, brief biological information is provided for two species, *S. falleni* Stål, 1863, and *S. croceus* Hsiao, 1979.

## 1. Introduction

The genus *Sycanus* Amyot & Serville, 1843 (Heteroptera: Reduviidae: Harpactorinae), now comprises 76 Oriental and 1 Madagascar species [1,2,3,4]. Prior to this study, a total of 12 species belonging to the genus *Sycanus* have been recorded within China [3,5,6,7,8,9,10,11]. This genus can be distinguished from other Chinese allied genera (*Agriosphodrus*, *Homalosphodrus*, *Maldonadocoris*, *Yolinus*, all belonging to the tribe Sycanini Dohrn, 1859) by the following characters: the head is significantly longer than the pronotum, the scutellum is armed with a spine or tubercle at its apex, and the connexivum of the abdomen is extremely laterally dilated [8,9].

However, there remain problems in the taxonomic study of *Sycanus* from China. One of the problems is the morphological similarity between *S. sichuanensis* Hsiao, 1979, distributed in the South China mainland, and some *Sycanus* specimens on its adjacent continental islands—Hainan and Taiwan—which makes it hard to determine whether these two groups of specimens are two independent species or if they should all be one mainland–island species. The same is the case between *S. croceus* Hsiao, 1979, distributed in the Chinese mainland, and *S. insularis* Hsiao, 1979, distributed in Hainan island. The other problem arises in *S. minor* Hsiao, 1979, wherein the body coloration variations are so significant that the identification and distinction of this species has become challenging. Furthermore, in *S. bifidus* (Fabricius, 1787), individuals of the same population raised in laboratory conditions often exhibit two different and stable coloration patterns, which is a phenomenon also observed in *S. marginellus* Putshkov, 1987, and *S. falleni* Stål, 1863. The aforementioned issues are interspecific or intraspecific morphological variations, which have been a source of confusion thus far and posed a significant obstacle for taxonomists who rely on traditional morphological methods to address the issue of species identification or delimitation.

Since the 21st century, DNA barcoding technology that utilizes the mitochondrial cytochrome *c* oxidase subunit I gene (COI) has emerged as a pivotal tool in insect identification, species delimitation, and species divergence evolution [12,13,14,15,16,17,18]. The integrative taxonomic method especially, which combines DNA barcoding molecular data and morphological evidence, is one of the developments in modern insect taxonomy and could provide clarity regarding the taxonomic status of species [15,16,17,18]. However, several studies have also demonstrated that DNA barcoding technology may not always be effective in resolving species delimitation and, in some cases, lead to an overestimation of species diversity or the potential suppression of the traditional morphological taxonomy [19,20,21]. In recent years, we have collected and examined a large number of specimens of *Sycanus* from China and extracted DNA from representative samples of each species to examine the validity of the DNA barcoding technology in distinguishing species of this genus and combining the morphological study to solve debatable taxonomic issues and potentially make new discoveries.

In the present study, the 14 species of *Sycanus* in China were described or redescribed, morphologically compared, and photographed in detail [3,5,6,7,8,9,10,11]; among them, we discovered three new species: *S. hainanensis* Wang & Cai sp. nov., *S. flavicorius* Li & Cai sp. nov., and *S. taiwanensis* Zhao & Cai sp. nov. We did not find the molecular samples of *S. collaris* and *S. fuscirostris* during the examination of all the collections we visited in China. With the exception of *S. collaris* and *S. fuscirostris*, the species delimitation and the phylogenetic analysis were performed for 12 Chinese *Sycanus* species, based on the COI DNA barcode dataset of 81 samples.

## 2. Materials and Methods

### 2.1. Specimens and Acronyms

This study is mainly based on the materials deposited in the Entomological Museum of China Agricultural University, Institute of Zoology of Chinese Academy of Sciences, and Nankai University. The following abbreviations for public collections are used:

BMNH, Natural History Museum, London, UK; 

CATAS, Chinese Academy of Tropical Agricultural Sciences, Haikou, Hainan, China;

CAU, Entomological Museum of China Agricultural University, Beijing, China; 

IOZ, Institute of Zoology, Chinese Academy of Sciences, Beijing, China;

MfN, the Museum of Natural History, Leibniz Institute for Evolution and Biodiversity Science, Berlin, Germany (Museum für Naturkunde—Leibniz-Institut für Evolutions- und Biodiversitätsforschung);

NKU, College of Life Sciences, Nankai University, Tianjin, China; 

NNU, Nanning Normal University, Nanning, Guangxi, China;

NRM, Swedish Museum of Natural History, Stockholm, Swedish;

TJNHM, Tianjin Natural History Museum, Tianjin, China;

ZMUC, the Natural History Museum of Denmark, Copenhagen, Denmark.

Specimens for molecular experiments were collected from Southern China (Table A1). All specimens were preserved in 100% ethanol and stored at −20 °C until DNA extraction. We finally obtained a set of COI DNA barcoding dataset (675 bp) from 81 samples of 12 Chinese *Sycanus* species (Alignment S1) used for the molecular study. All sample codes used in present study are shown in Table A1. The GenBank Accession numbers **(OP927069–OP927154)** corresponding to code of every sample are shown in Table S1.

### 2.2. Taxonomy

The morphological characters were observed using the stereoscopic microscope. The male pygophore was put into a hot 90℃ lactic acid solution for about 10–15 min to get rid of muscle and fat, then instantly put into hot boiling distilled water. A few minutes later, the endosoma extended naturally from the phallus, or we pulled it out of the phallus using a pair of pointed tweezers. The dissected parts of the genitalia were placed in a plastic micro vial with lactic acid under the corresponding specimen. Habitus photographs of specimens deposited in CAU, CATAS, IOZ, NKU, NNU, TJNHM were taken using the Canon D90 SLR camera (Canon Inc., Tokyo, Japan). The photographs of type specimens deposited in MfN were taken using a Novoflex Castel-Micro Stack shot system together with a Sony Alpha 7R III Macro G OSS with FE 90 mm F2.8 (about 28 stacks). Photographs of male genitalia were taken with the aid of the Research Stereo Microscope SMZ25 (Nikon Corporation, Tokyo, Japan). The drawings were traced with the aid of a camera lucida. All measurements are in millimeters. The measurements were based on the specimens deposited in CAU. The classification system, the classification identification, and the morphological terminology mainly followed Distant, Hsiao, and Hsiao and Ren [8,9,10]. 

### 2.3. Integrative Molecular Analysis

#### 2.3.1. DNA Extraction and Sequencing

Muscle tissue was taken from the meso- and meta-thoraxes for DNA extraction. The total DNA was extracted using the Animal Tissue Extraction Kit (Golden EasyPure^®^ Genomic DNA Kit, TransGen Biotech, Beijing, China) and stored at –20 °C. COI DNA barcodes were amplified with the primers COI-F (5′-TGATCAGGTGTAGTGGGGAC-3′) and COI-R (5′-TCCCTAATGACCCGAAAGCT-3′). The PCR reaction system consisted of a total volume of 50 µL containing 1 µL DNA template, 25 µL 2× EasyTaq^®^PCR SuperMix (TransGen Biotech, Beijing, China), 1 µL each of forward and reverse primers (10 µM), and 22 µL nuclease-free water. The reaction procedure consisted of pre-denaturation (94 °C, 3.5 min) and 35 cycles for denaturation (94 °C, 30 s), annealing (56 °C, 30 s), elongation (72 °C, 1 min), and elongation (72 °C, 8 min). The PCR products were detected with 2% agarose gel electrophoresis. For all specimens, sequences were obtained and bidirectionally sequenced using the same PCR primer pairs. Qingke Biotechnology Co., Ltd. (Hangzhou, China), mainly completed the sequencing of PCR amplicons using the Sanger sequencing method.

#### 2.3.2. Phylogenetic Analyses

A total of COI DNA barcodes of 81 individuals were successfully obtained (Table A1 and Table S1). In addition, six individuals of four species from the three closely related genera (*Agriosphodrus*, *Maldonadocoris*, *Yolinus*) were selected as outgroups (Table A1 and Table S1). Multiple sequence alignment was performed using ClustalW in MEGA X [22]. The resultant sequences were 675 bp for COI DNA barcodes.

The maximum-likelihood (ML) phylogenetic tree (Figure S1) was constructed using IQ-TREE web server [23] by an ultrafast bootstrap approximation approach with 1000 replicates. The best evolutionary model was selected as HKY+F+I+G4 according to BIC (Bayesian Information Criterion) in IQ-TREE web server [23].

The neighbor-joining (NJ) tree (Figure S2) was performed using MEGA X [22] based on the Kimura-2-Parameter (K2P) model [24] with 1000 bootstrap replicates.

**Figure 1:**
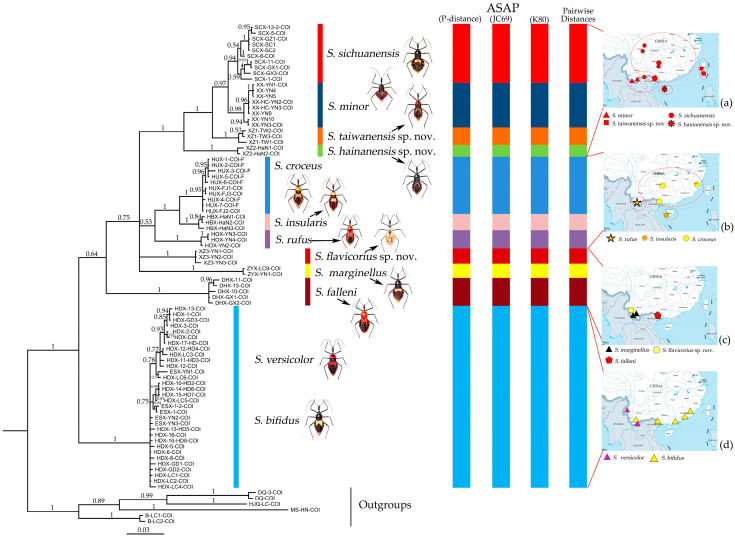
Bayesian phylogenetic tree of cytochrome *c* oxidase subunit I (COI) sequence for 81 terminals of *Sycanus*; the results of Bayesian inference (BI) analyses agreed with those of the two different species delimitation approaches, pairwise distances, and ASAP (the assemble species by automatic partitioning, p-distance, JC69, K80) analysis. The numbers above the branches were the posterior probabilities (>0.50) of the Bayesian inference (BI). The clades/species identified in this study were indicated in different colors. (**a**–**d**); distribution sites of samples used in molecular study for each *Sycanus* species in China were mapped, (**a**) Group 1: *Sycanus sichuanensis* Hsiao, 1979; *Sycanus minor* Hsiao, 1979; *Sycanus hainanensis* Wang & Cai sp. nov.; and *Sycanus taiwanensis* Zhao & Cai sp. nov. (**b**) Group 2: *Sycanus croceus* Hsiao, 1979; *Sycanus insularis* Hsiao, 1979; and *Sycanus rufus* Hsiao, 1979. (**c**) Group 3: *Sycanus falleni* Stål, 1863; *Sycanus flavicorius* Li & Cai sp. nov.; and *Sycanus marginellus* Putshkov, 1987. (**d**) Group 4: *Sycanus versicolor* Dohrn, 1859, and *Sycanus bifidus* (Fabricius, 1787).

The Bayesian inference (BI) tree (Figure 1) was generated using MrBayes 3.2.7 [25] with two simultaneous Markov Chain Monte Carlo (MCMC) runs of two million generations and tree sampling every 1000 generations. The best-fit model was HKY+G through the prediction of PartitionFinder 2 [26], chosen according to BIC. The first 25% of trees were discarded as burn-in.

#### 2.3.3. Species Delimitation

We employed two different species-delimitation methods for the COI dataset of *Sycanus*. In the estimates of evolutionary divergence over sequence pairs, we divided 12 *Sycanus* species from a total of 81 individuals in accordance with the morphological characters. Then, we utilized the Kimura-2 parameter (K2P) model [24] within MEGA X software [22] to compute intraspecific and interspecific pairwise genetic distances (p-distance) among these 12 pre-delimited species as well as interspecific pairwise genetic distances across all 81 individuals (Tables S2 and S3).

The assemble species by automatic partitioning (ASAP) analysis [27] was performed using a fasta file containing COI DNA barcode dataset of 81 individuals belonging to the genus *Sycanus* in China. The analysis utilized the Kimura (K80) TS/TV 2.0 model, simple distant (p-distance) method, and Jukes–Cantor (JC69) method for species delimitation or partition of the molecular operational taxonomic units (MOTUs) (Figure 1).

### 2.4. Biological Study

We collected live insects of *S. falleni* Stål, 1863, and *S. croceus* Hsiao, 1979, from Huashan, Ningming, Guangxi, China. They were reared on larvae of yellow mealworm (*Tenebrio molitor*). The annual life history of both species under experimental conditions (26 °C, natural light) was documented. The eggs and 1st to 5th instar nymphs were morphological described and illustrated (Tables S4 and S5).

## 3. Results 

### 3.1. Species Identification Based on Morphological Characters

Based on the morphological characters, all specimens were initially identified as belonging to 12 distinct morphological species, mainly based on the taxonomic works of Distant, Hsiao, and Hsiao and Ren [8,9,10]. Figure 2 presented the habitus of the 12 species of the genus *Sycanus* found in China and depicted the interspecific variations in body coloration and shape. Figure 3 illustrated the morphological structures of the head, pronotum, and scutellum and highlighted notable differences in the apical spine of scutellum across different species. Figure 4 presented the lateral view of the abdomen as an important distinguishing feature among *Sycanus* species. Figure 5 and Figure 6 recorded the life history and the morphological structure of eggs and nymphs for *S. falleni* and *S. croceus*. The morphological characters for 12 *Sycanus* species were illustrated in Figure 7, Figure 8, Figure 9, Figure 10, Figure 11, Figure 12, Figure 13, Figure 14, Figure 15, Figure 16, Figure 17, Figure 18, Figure 19, Figure 20, Figure 21, Figure 22, Figure 23, Figure 24, Figure 25, Figure 26, Figure 27, Figure 28, Figure 29, Figure 30 and Figure 31 of the Systematics section in detail.

Compared with the external morphology and body color changes of the 12 Chinese *Sycanus* species, the male genitalia structure can better reflect the genetic relationship between the species. Based on the structural characters of male genitalia, we divided the Chinese 12 *Sycanus* species into four groups: 

(1) Group 1: “*S. sichuanensis* species group” includes *S. sichuanensis* Hsiao, 1979; *S. minor* Hsiao, 1979; *S. taiwanensis* sp. nov.; and *S. hainanensis* sp. nov., which have nearly common morphological characters in their male genitalia; the median pygophore process is “T” shaped, the central part of the endosoma is with two weakly sclerotized stripe-shaped sclerites, and the apical part of the endosoma has five or six pairs of spines and medially has a feebly sclerotized horned process (Figure 17, Figure 23, Figure 27 and Figure 29).

*Sycanus sichuanensis* is widely distributed in South China and Vietnam; *S. minor* is only distributed in southwest Guangxi and southern Yunnan in China and Vietnam. Two new cryptic species, *S. taiwanensis* sp. nov. and *S. hainanensis* sp. nov., are separately distributed in Taiwan and the Hainan islands (Figure 1a).

(2) Group 2: “*S. croceus* species group” consisted of *S. croceus* Hsiao, 1979; *S. insularis* Hsiao, 1979; and *S. rufus* Hsiao, 1979, which have common features in the male genitalia; the median pygophore process is “Y”-shaped, the central part of the endosoma is with two well-sclerotized horned processes, the apical part is armed laterally with two or three pairs of larger spines, and 8–11 pairs of smaller spines (Figure 10, Figure 19 and Figure 25).

*Sycanus croceus* is widely distributed in South China and Vietnam; *S. insularis* is only in Hainan Island, China; and *S. rufus* is only in southern Yunnan, China (Figure 1b).

(3) Group 3: *S. marginellus* Putshkov, 1987; *S. flavicorius* sp. nov.; and *S. falleni* Stål, 1863, have similar characters in the structure of male genitalia; the median pygophore process is prominently posteriorly produced, while its shape is different among them. The paramere is clavate, the apical half is swelled with thick setae, and the middle part is distinctly bent, but in *S. falleni*, the apical half of the paramere is wider and slightly compressed; the basal plate bridge of the phallobase is thinner than the basal plate. The dorsal phallothecal sclerite is constricted apically, the endosoma is medially with two thin weakly sclerotized sclerites, and the apical part is with many small angular spines in *S. falleni*, many small processes in *S. flavicorius* sp. nov., or some small processes and six lateral pairs of small spines in *S. marginellus* (Figure 12, Figure 14 and Figure 21).

*Sycanus marginellus* is distributed in Yunnan, China; *S. flavicorius* sp. nov. is only in Yunnan, China; *S. falleni* is mainly in southwest Guangxi and southern Yunnan in China and Myanmar, Cambodia, India, and Vietnam (Figure 1c).

(4) Group 4: “*S. bifidus* species group” consisted of *S. versicolor* Dohrn, 1859, and *S. bifidus* (Fabricius, 1787), which have similar characters in the male genitalia: the median pygophore process is with two ear-shaped processes and a median large posteriorly produced process; the apical half of paramere is swelled with thick setae; the dorsal phallothecal sclerite is widened laterally; and the endosoma is medial with two weakly sclerotized stripe-shaped sclerites, apical with a well-sclerotized horned process, and lateral with five or six pairs of small spines (Figure 8 and Figure 31).

*Sycanus bifidus* is distributed in southern Yunnan; southeast and south coastal regions of China and Myanmar, India, Vietnam, and Malaysia; and *S. versicolor* is in southern Yunnan, China, and Bengalen, Penang (Figure 1d).

### 3.2. Phylogenetic Relationships and Species Delimitation

#### 3.2.1. Phylogenetic Relationships

The COI DNA barcode dataset (Alignment S1) comprised a total of 81 ingroup individuals from 12 delimited morphological species and 6 outgroup individuals from 3 closely related genera. After trimming both ends of the alignment, the dataset with a sequence length of 675 bp was utilized for subsequent analyses. Within the COI sequence matrix, 433 sites (64.15% of all sites) were invariant (constant or ambiguous constant), 242 sites (35.85% of all sites) were variant, and 215 sites (31.85% of all sites) were parsimony informative.

The phylogenetic trees, constructed using three approaches (Bayesian inference, BI; maximum likelihood, ML; neighbor-joining, NJ), showed some differences in the topological structure of the clades (Figure 1 and Figures S1 and S2). The *Sycanus* species, which owned similar characteristics in the structure of their male genitalia, had a closer relationship in the phylogenetic analysis. The clades, referred to as the “*S. sichuanensis* species group” (Group 1) and “*S. croceus* species group” (Group 2) mentioned above, were both supported in the BI (Figure 1a,b), ML, and NJ trees. These two species groups always coexisted within the same clade (Figure 1 and Figures S1 and S2).

Group 3 consisted of *S. marginellus*, *S. flavicorius* sp. nov., and *S. fallen*, sharing a common clade with the *S. sichuanensis* species group and *S. croceus* species group in the BI tree (Figure 1c). However, *S. falleni* did not group together with the aforementioned species in both ML and NJ trees (Figures S1 and S2). Therefore, the BI tree was more consistent with the morphological results based on the structure of male genitalia (Figure 1c).

In the phylogenetic analysis, the individuals of *S. versicolor* and *S. bifidus* were mixed together (Group 4; Figure 1d and Figures S1 and S2). The group consisting of *S. versicolor* and *S. bifidus* formed the species complex (Group 4), which was located at the basal part of the ingroup in the BI tree (Figure 1d).

**Figure 2:**
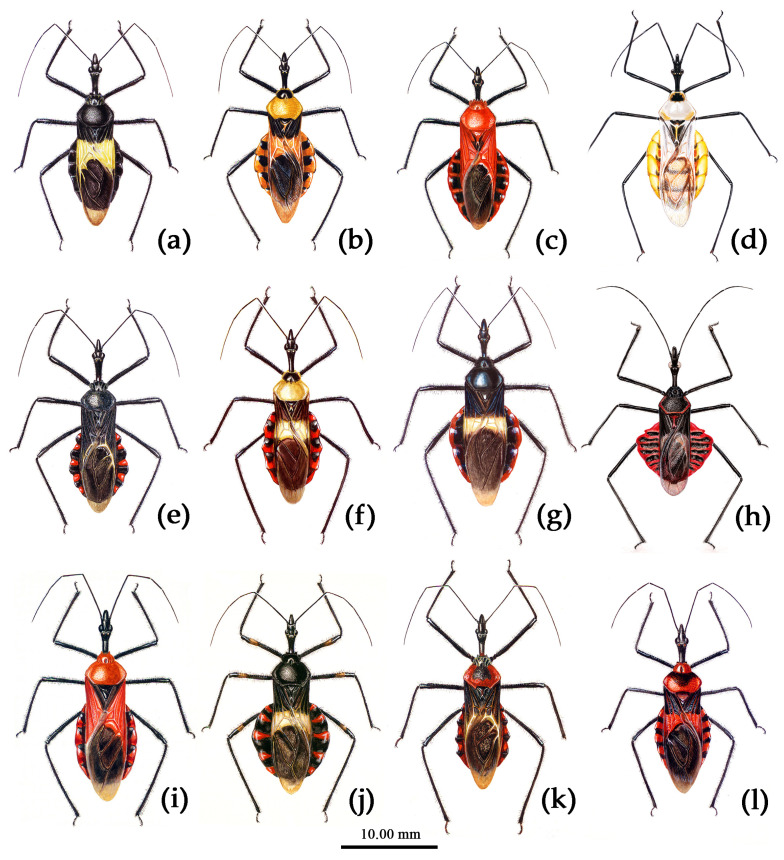
*Sycanus* spp. in China, habitus. (**a**) *Sycanus bifidus* (Fabricius, 1787); (**b**) *Sycanus croceus* Hsiao, 1979; (**c**) *Sycanus falleni* Stål, 1863; (**d**) *Sycanus flavicorius* Li & Cai sp. nov.; (**e**), *Sycanus hainanensis* Wang & Cai sp. nov.; (**f**), *Sycanus insularis* Hsiao, 1979; (**g**) *Sycanus marginellus* Putshkov, 1987; (**h**) *Sycanus minor* Hsiao, 1979; (**i**) *Sycanus rufus* Hsiao, 1979; (**j**) *Sycanus sichuanensis* Hsiao, 1979; (**k**) *Sycanus taiwanensis* Zhao & Cai sp. nov.; (**l**) *Sycanus versicolor* Dohrn, 1859.

**Figure 3:**
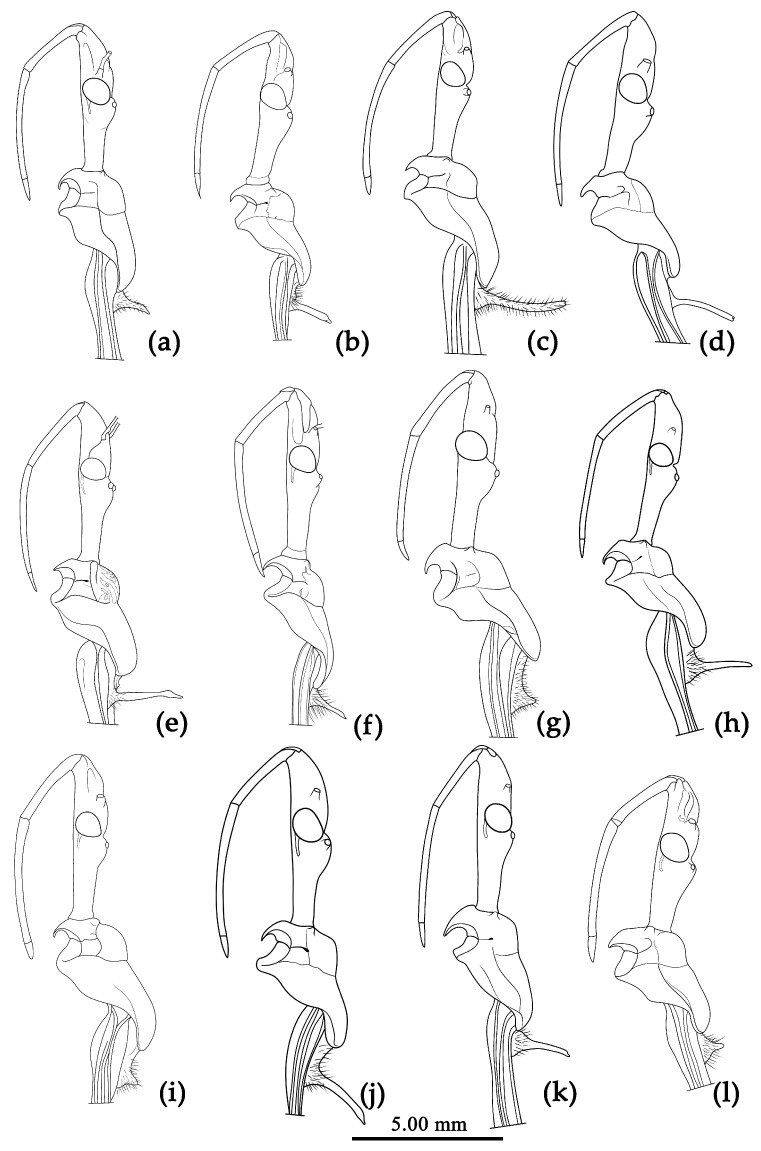
*Sycanus* spp. in China, outline of lateral view of head, pronotum and scutellum. (**a**) *Sycanus bifidus* (Fabricius, 1787); (**b**) *Sycanus croceus* Hsiao, 1979; (**c**) *Sycanus falleni* Stål, 1863; (**d**) *Sycanus flavicorius* Li & Cai sp. nov.; (**e**) *Sycanus hainanensis* Wang & Cai sp. nov.; (**f**) *Sycanus insularis* Hsiao, 1979; (**g**) *Sycanus marginellus* Putshkov, 1987; (**h**) *Sycanus minor* Hsiao, 1979; (**i**) *Sycanus rufus* Hsiao, 1979; (**j**) *Sycanus sichuanensis* Hsiao, 1979; (**k**) *Sycanus taiwanensis* Zhao & Cai sp. nov.; (**l**) *Sycanus versicolor* Dohrn, 1859.

**Figure 4:**
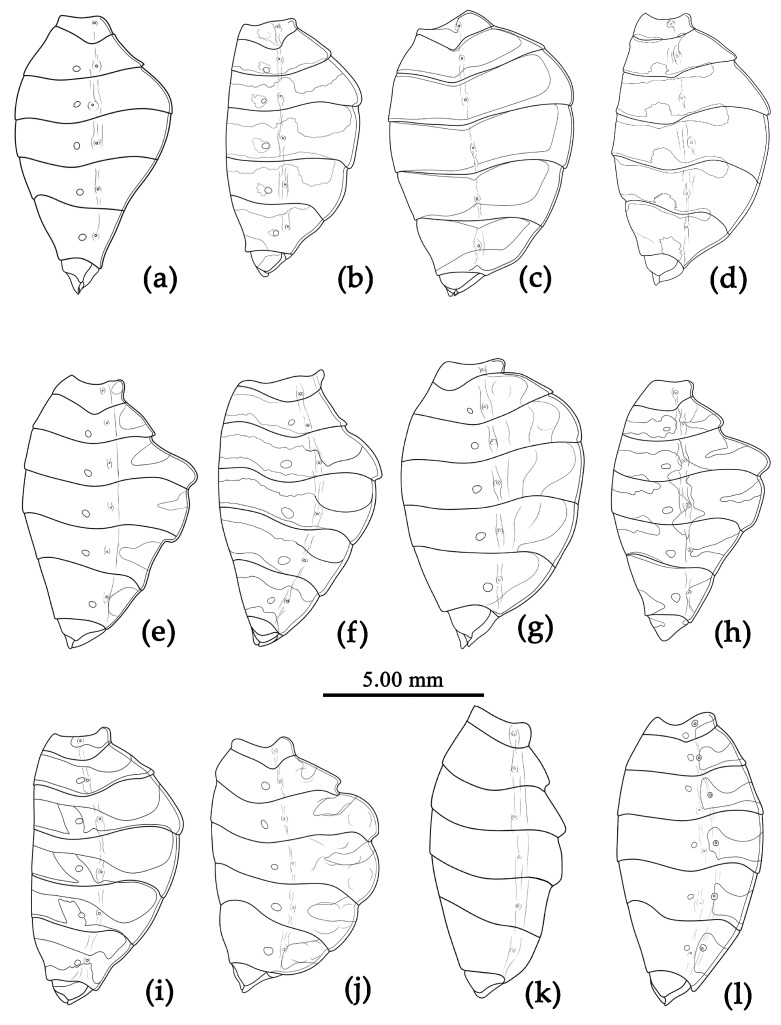
*Sycanus* spp. in China, outline of lateral view of abdomen. (**a**) *Sycanus bifidus* (Fabricius, 1787); (**b**) *Sycanus croceus* Hsiao, 1979; (**c**) *Sycanus falleni* Stål, 1863; (**d**) *Sycanus flavicorius* Li & Cai sp. nov.; (**e**) *Sycanus hainanensis* Wang & Cai sp. nov.; (**f**) *Sycanus insularis* Hsiao, 1979; (**g**) *Sycanus marginellus* Putshkov, 1987; (**h**) *Sycanus minor* Hsiao, 1979; (**i**) *Sycanus rufus* Hsiao, 1979; (**j**) *Sycanus sichuanensis* Hsiao, 1979; (**k**) *Sycanus taiwanensis* Zhao & Cai sp. nov.; (**l**) *Sycanus versicolor* Dohrn, 1859.

Due to the utilization of only 675 bp COI DNA barcodes, the results for phylogenetic analysis based on different methods are kind of unstable in the present study. However, the molecular phylogenetic trees (Figure 1 and Figures S1 and S2), especially the BI tree (Figure 1), provided strong support for the species delimitation and species group divisions derived from the morphological characters of male genitalia and the geography distribution, with the exception of *S. versicolor* and *S. bifidus.* All of our phylogenetic analyses failed to delimit these two species (Figure 1d, Figure 2a,l, Figure 3a,l, Figure 4a,l and Figures S1 and S2).

#### 3.2.2. Species Delimitation

The COI DNA barcode dataset of 81 individuals from 12 pre-delimited species was used to compute intraspecific and interspecific pairwise genetic distances (p-distance). The mean divergence for the entire dataset was 9.79%. The p-distances between the 12 pre-delimited species (except *S. versicolor* and *S. bifidus*) ranged from 3.02% to 19.69% (Table S2). The p-distances between individuals of *S. versicolor* and *S. bifidus* ranged from 0.15% to 1.96%, while it was 1.05% between the two pre-delimited species (<2.00%) [13]. The p-distances between the 81 sampling individuals ranged from 0% to 17.67% (Table S3). The intraspecific p-distances within the 12 pre-delimited species ranged from 0.15% to 1.16% (Table S2).

The ASAP analysis produced ten kinds of partition results, corresponding to different ASAP scores ranging from 2.50 to 10.00 (10 groups/MOTUs at a score of 2.50; 7 groups at a score of 3.00; 8 groups at a score of 4.00; 11 groups at a score of 6.50; 64 groups and 49 groups at a score of 7.00; 37 groups at a score of 8.00; 6 groups and 9 groups at a score of 8.50; and 47 groups at a score of 10.00). The ASAP scale of the 10 group/MOTU partition was 2.50, which was the lowest score among them (a lower score indicates a better partition) [27]. The difference between the partition result consisting of 11 groups and that of 10 groups primarily arose from the closer relationship of *S. sichuanensis* and *S. minor*, but the p-distance between them was 3.02% (>2%) [13], and the body coloration and shape were distinctly different (Figure 2h,j, Figure 3h,j, Figure 4h,j, Figure 22 and Figure 26). Therefore, we selected and accepted the partition result of 11 groups/MOTUs (ASAP score: 6.50) based on the results of interspecific pairwise genetic distances (Table S2) and morphological comparison. The 11 MOTUs delimited by the ASAP analysis were also found to have strong support in the BI, ML, and NJ trees. In addition, within the 9 group/MOTU partition, *S. insularis* and *S*. *croceus* were merged into a single species, but it was worth noting that the p-distance between these two species was 4.28% (>2%) [13] (Table S2). The genetic distance between *S. versicolor* and *S. bifidus* was 1.05%, and the ASAP analysis also failed to differentiate them.

**Figure 5:**
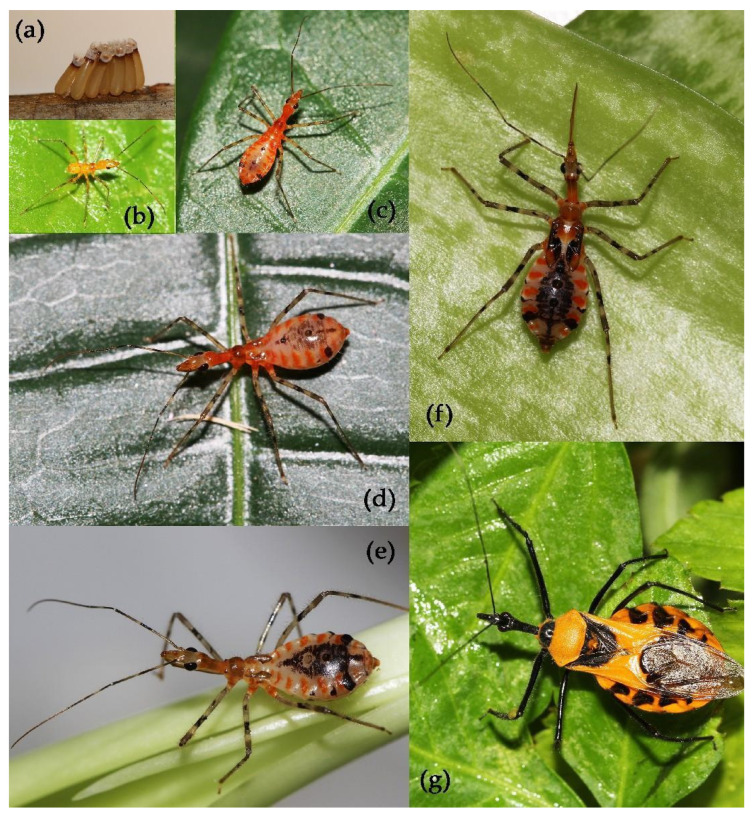
*Sycanus croceus* Hsiao, 1979, (**a**) egg mass; (**b**) first instar; (**c**) second instar; (**d**) third instar; (**e**) fourth instar; (**f**) fifth instar; (**g**) adult.

### 3.3. Biology

#### 3.3.1. *Sycanus croceus* Hsiao, 1979

We documented the annual life history of *Sycanus croceus* Hsiao, 1979, based on the live insects collected from Ningming, Guangxi, China (Figure 5; Table S4). The species occurs in one generation per year. The overwintering adults occur in large numbers in May. The females in laboratory conditions laid about 60 eggs. The egg surface had less mucus secretion, and the eggs were not well adhered to each other but rather formed several smaller egg masses (Figure 5a). They prefer to lay eggs on the surface of plant stems (Figure 5a). The 1st to 5th instar nymphs were shown in the Figure 5b–f. The nymph stage is 156 ± 4.16 days from late June to early December (Table S4). The adults emerge from early October to late December. The adults or 5th nymph overwinter from early November to April of the following year, and the adults peak in May (Figure 5g), then begin to reproduce in June and July. The adults (females and males) generally die in late June.

**Egg**. The egg is yellowish brown, 2.95–3.00 mm in length, 0.95–1.00 mm in width, and elongate oblong. The egg gradually changes from yellowish brown to reddish brown, with a metallic luster; the operculum is white (Figure 5a).

**Coloration of nymph**. The body coloration of the 1^st^ to 5^th^ instar nymphs is similar. The body of a nymph is orange with black and yellowish markings (Figure 5b–f). The anterior lobe of the head is yellowish brown; the posterior lobe is orange. The rostrum is yellowish brown. The first to third antennal segments and the basal part of the fourth are black, but two median annular markings of the first and the median annular markings of the second are greyish white, most of the fourth is reddish brown, and the apical part is black-brown. The eyes are black. The femora are yellowish white, with an apical grey annular marking, with a medial black annular marking, and with a basal irregular black marking; the tibiae are pale greyish brown, the sub-basal annulation of tibiae is black, and the basal part and the apical part are greyish brown. The tarsi are pale greyish brown. The wing pads of the 3rd to 5th instar nymphs is orange, the inner side is black. The abdomen is yellowish-white with orange stripes on two sides of each segment, with dorsally central leaf-shaped black markings and with two posterior round black spots, and the three dorsal abdominal glands of the third to fifth abdominal segments are black.

**Structure of nymph.** The body is covered with pale, long setae. The first antennal segment and basal half of the second are sparsely covered with long setae, and the apical half of the second, the third, and the fourth are covered with yellowish brown short setae. The legs are sparsely covered with yellowish brown setae of various lengths. The body is posteriorly widened; the head is elongate and spindle-shaped. The fourth antennal segment is the longest and slightly longer than the first, and the second is the shortest and somewhat shorter than the third. The second rostral segment is the longest and longer than first and third segments together; the ocelli is absent; the legs and antennae are slender; and the wing pads of the third to fifth instar nymphs are visible (Figure 5b–f). The abdomen is dilated on two sides.

**Measurement of nymph [1st: 2nd: 3rd: 4th: 5th, in mm].** Body length 3.40–3.45: 5.09–5.10: 5.95–6.00: 9.45–9.50: 16.00–15.63; maximal width of abdomen 1.00–1.09: 1.45–1.50: 1.82–1.90: 2.70–2.73: 4.55–4.60; head length 1.00–1.09: 1.60–1.64: 2.18–2.20: 2.70–2.73: 4.55–4.60; length of antennae I/II/III/IV = 1.27–1.30: 1.80–1.82: 2.55–2.60: 3.27–3.30: 4.55–4.60/0.36–0.40: 0.80–0.82: 1.09–1.10: 1.45–1.50: 1.80–1.82/0.18–0.20: 0.30–0.33: 0.40–0.42: 0.90–0.91: 1.45–1.50/1.60–1.64:2.18–2.20:2.70–2.73:3.27–3.30:?; length of visible rostrum I/II/III= 0.36–0.40: 0.55–0.60: 0.70–0.73: 1.27–1.30: 1.80–1.82/0.70–0.73: 1.09–1.10: 1.45–1.50: 2.18–2.20: 3.09–3.10/0.10–0.12: 0.18–0.20: 0.36–0.40: 0.55–0.60: 0.90–0.91; length of thorax 0.60–0.64: 0.90–0.91: 1.27–1.30: 2.18–2.20: 3.60–3.64; maximal width of pronotum 0.36–0.40: 0.90–0.91: 0.90–0.91: 1.27–1.30: 2.55–2.60; length of wing pad /:/:0.36–0.40:0.90–0.91:2.70–2.73.

#### 3.3.2. *Sycanus falleni* Stål, 1863

**Figure 6:**
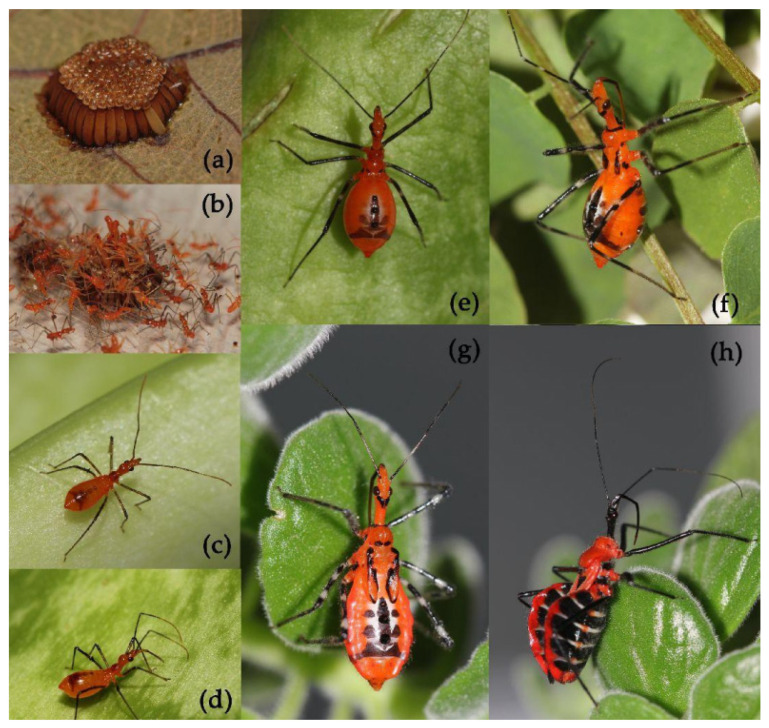
*Sycanus falleni* Stål, 1863, (**a**) egg mass; (**b**,**c**) first instar, hatching; (**d**) second instar; (**e**) third instar; (**f**) fourth instar; (**g**) fifth instar; (**h**) adult.

We documented the part of the annual life history of *Sycanus falleni* Stål, 1863, based on the live insects collected from Ningming, Guangxi, China (Figure 6; Table S5). They occur one generation per year in Ningming. Female adults lay eggs on the surface of leaves and stems of plants in an egg mass with more than 100 eggs (Figure 6a). The morphology of the 1st–5th instar nymphs is shown in Figure 6b–f. The stage of the 1st–5th instar nymphs is 111.20 ± 3.35 days from early August to late December (Table S5). The adults emerge in December (Figure 6g). 

**Egg.** The egg is yellowish brown, 3.50–3.55 mm in length, 0.95–1.00 mm in width, and elongate oblong. The egg gradually changes from yellowish brown to reddish brown, with a metallic shine; the operculum is pale brown (Figure 6a).

**Coloration of nymph.** The body color of the 1st to 5th instar nymphs is similar. The body of the nymph is red with black and white markings (Figure 6). The head is red with black markings on the vertex. The first to third antennal segments and the basal part of the fourth are black; two median annular markings of the first, median annular markings of the second are greyish white; and most of the fourth is brown. The eyes are black. The rostrum is black; the apical part is brown. The legs are black; the basal, median, and sub-apical annular markings of the femora are yellowish white; and the apical half and the sub-basal part of the tibiae are blackish brown. The abdomen has a dorsally central longitudinal black stripe and four black spots and posteriorly a transversal black stripe, and the three dorsal abdominal glands are black. The abdomen has ventral black and white transversal stripes. The wing pads of the 3rd to 5th instar nymphs are red with black markings. 

**Structure of nymph.** The body of the nymph is covered with pale setae. The body is widened gradually posteriorly. The head is robust and elongate. The antennae are slender, the fourth segment is the longest and slightly longer than the first, and the third segment is the shortest. The second rostral segment is subequal to the first and third segments together in length. The legs are slender. The wing pads of the 3rd to 5th instar nymphs are visible. The abdomen is dilated on two sides. 

**Measurement of nymph [1st: 2nd: 3rd: 4th: 5th, in mm].** Body length 4.70–4.73: ?: 7.60–7.64: 9.09–9.10: ?; maximal width of abdomen 1.40–1.45: ?: 2.50–2.55: 3.60–3.64: ?; head length 1.45–1.50: ?: 2.18–2.20: 3.27–3.30: ?; length of antennae I/II/III/IV= 1.80–1.82: ?: 3.09–3.10: 3.80–3.82: ?/0.70–0.73: ?: 0.95–1.00: 1.45–1.50: ?/0.20–0.22: ?: 0.36–0.40: 0.70–0.73: ?/2.18–2.20: ?: 3.27–3.30: 4.55–4.60:?; length of visible rostrum I/II/III= 0.55–0.60: ?: 0.80–0.82: 1.09–1.10: ?/0.90–0.91: ?: 1.60–1.64: 1.60–1.64: ?/ 0.36–0.40: ?: 0.36–0.40: 0.55–0.60: ?; length of thorax 1.27–1.30: ?:2.36–2.40: 2.70–2.73: ?; maximal width of pronotum 0.70–073: ?: 1.45–1.50: 1.80–1.82: ?; length of wing pad /: /: 0.39–0.40: 1.45–1.50: 4.19–4.20.

### 3.4. Systematics

**Genus *Sycanus* Amyot & Serville, 1843**
*Sycanus* Amyot & Serville, 1843: 360 [1], type species by monotypy: *Reduvius collaris* Fabricius, 1781 [2]; Hsiao, 1979: 154, 159, keyed nine Chinese species [9].
*Cosmosphodrus* Stål, 1867: 278 [28], type species by subsequent designation (Putshkov, Putshkov & Štys, 1987 [29]): *Sycanus generosus* Stål, 1863, synonymized by Lethierry & Severin, 1896: 171 [30]; Stål, 1874: 28, as subgenus of *Sycanus* [31]; Putshkov & Putshkov, 1985: 4 [32]; Putshkov, Putshkov & Štys, 1987: 103 [29].

**Diagnostic characters.** Body elongate ovate, median to large sized. Head long, somewhat slender, about as long as pronotum and scutellum together, postocular part much longer than anteocular; rostrum slender, first segment longer than anteocular part of head, and distinctly shorter than second segment; first antennal segment about as long as fore femur. Pronotum unarmed and constricted before middle, anterior pronotal lobe small and bulged, much shorter and narrower than posterior lobe, its median longitudinal sulcus short and deeply depressed; posterior lobe rugosely punctate or wrinkled; posterior and lateral angles obtuse and rounded; posterior margin nearly straight; posterior margin of scutellum round, apical part with an erect long spine or tubercle, its apex sometimes bifid. Abdomen on each side strongly roundly dilated, connexivum more or less undulated.

**Distribution.** Oriental region (76), Ethiopian region (Madagascar) (1).

**The key to the Chinese species of the genus *Sycanus* Amyot & Serville, 1843**

1. Pronotum totally black··········································································································································································································2

-. Pronotum red or bicolor·········································································································································································································7

2. Scutellum with a short tubercle-shaped spine, its apex not bifid········································································································*Sycanus marginellus* Putshkov, 1987

-. Scutellum with a long spine, its apex bifid··········································································································································································3

3. Connexivum of abdomen with red markings·····································································································································································4

-. Connexivum of abdomen totally black················································································································································································5

4. Corium of fore wing black, its posterior margin yellowish························································································································*Sycanus hainanensis* Wang & Cai sp. nov.

-. Corium of fore wing yellowish, its basal part black··························································································*Sycanus sichuanensis* Hsiao, 1979

5. Corium of fore wing mostly greyish yellow, only basal part black·········································································································*Sycanus fuscirostris* Dohrn, 1859

-. Basal half of corium of fore wing black, apical half yellow··············································································································································6

6. Apical half of corium of fore wing golden-yellow·········································································································*Sycanus bifidus* (Fabricius, 1787)

-. Apical half of corium of fore wing stramineous·········································································································*Sycanus collaris* (Fabricius, 1781)

7. Apical spine of scutellum long, its apex bifid····································································································································································8

-. Apical spine of scutellum very short, its apex un-bifid···················································································································································13

8. Pronotum and corium of fore wing totally red·········································································································*Sycanus falleni* Stål, 1863

-. Pronotum and corium of fore wing partly red or yellow·················································································································································9

9. Posterior pronotal lobe red, median part with black markings; fourth and sixth abdominal connexiva laterally expanded·································10

-. Posterior pronotal lobe yellow or greyish yellow; abdomen laterally roundly expended···························································································11

10. Abdomen and connexivum red or yellow with black markings····························································*Sycanus minor* Hsiao, 1979

-. Abdomen totally black, connexivum bicolor····························································*Sycanus taiwanensis* Zhao & Cai sp. nov.

11. Corium of fore wing totally yellowish····························································*Sycanus flavicorius* Li & Cai sp. nov.

-. Basal part of corium of fore wing black, apical half yellow··············································································································································12

12. Posterior pronotal lobe and median transversal markings of fore wing pale grayish yellow; abdomen red, with black markings····························································*Sycanus insularis* Hsiao, 1979

-. Posterior pronotal lobe, and most of corium of fore wing (except inner side and most of clavus black) orange; abdomen orange, with black markings····························································*Sycanus croceus* Hsiao, 1979

13. Pronotum and corium of fore wing totally red; ventral surface of head paler; abdominal sterna yellow, inter-segment with black transversal stripe, and lateral side with black longitudinal stripe and round white markings; black markings of sixth and seventh connexival segments not extending to outer margin····························································*Sycanus rufus* Hsiao, 1979

-. Pronotum and corium of fore wing bicolor, black and red; ventral surface of head black; abdominal sterna black; black markings of connexivum totally extending to outer margin ····························································*Sycanus versicolor* Dohrn, 1859

**Figure 7:**
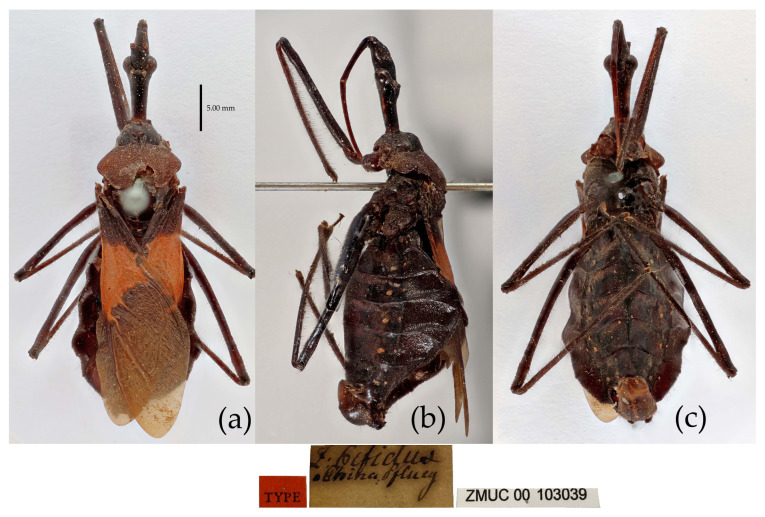
*Sycanus bifidus* (Fabricius, 1787), **Syntype**, habitus, male (ZMUC). (**a**) dorsal view; (**b**) lateral view; (**c**) ventral view.

**Figure 8:**
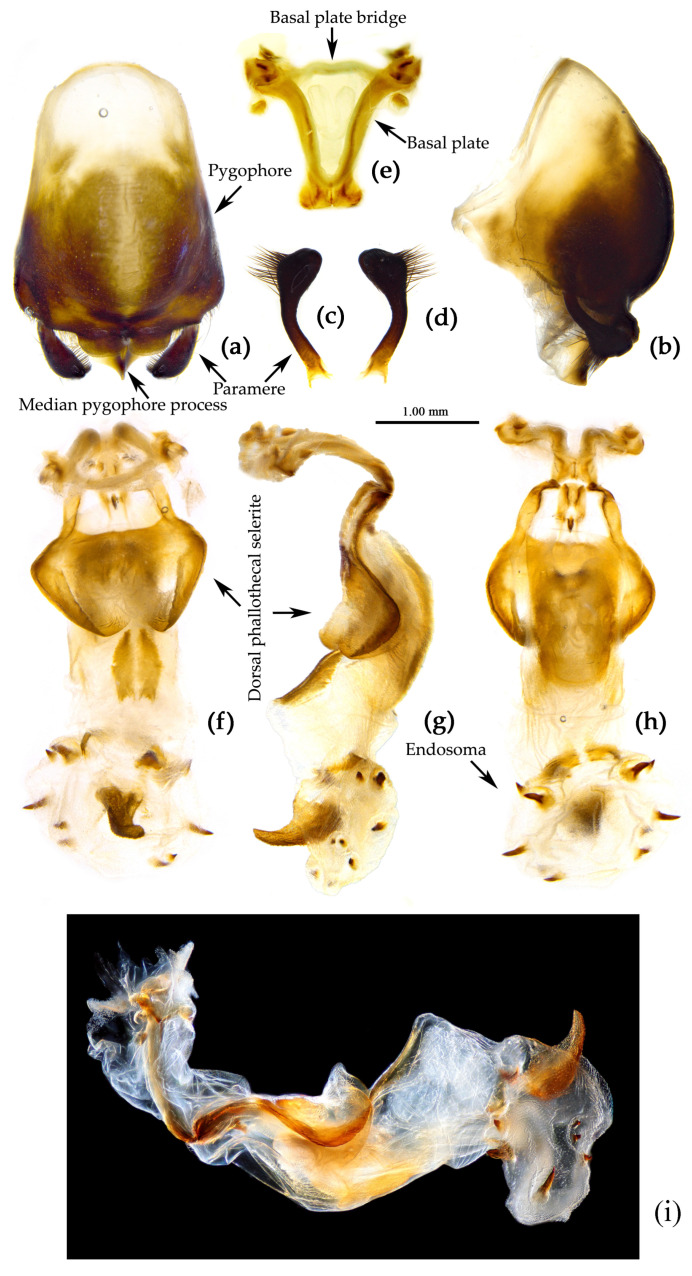
*Sycanus bifidus* (Fabricius, 1787), (**a**–**i**) male external genitalia; (**a**,**b**) pygophore with parameres; (**c**,**d**) paramere; (**e**) phallobase; (**f**–**i**) phallus; (**f**) dorsal view; (**b**,**g**,**i**) lateral view; (**a**,**h**) ventral view.

**(1) *Sycanus bifidus* (Fabricius, 1787)**
(Figure 2a, Figure 3a, Figure 4a, Figure 7, Figure 8 and Figures S3, S4, and S12a,b)
*Reduvius bifidus* Fabricius, 1787: 312 [5] (Figure 7); Zimsen, 1964: 339 [33].
*Cimex bifidus*: Gmelin, 1790: 2200 [34].
*Zelus bifidus*: Fabricius, 1803: 285 [35].
*Sycanus bifidus*: Dohrn, 1859: 97 [6]; Distant 1904: 353 [8]; Putshkov & Putshkov 1996: 259 [11]; Maldonado-Capriles 1990: 310 [3].
*Sycanus croceovittatus* Dohrn, 1859: 97 [6]; Hsiao & Ren, 1981: 519 [10]; Maldonado-Capriles, 1990: 311 [3]; Putshkov & Putshkov, 1996: 259 [11]. **syn. nov.** (Figure S3a,b)
*Sycanus villicus* Stål, 1863: 34 [7]. **syn. nov.** (Figure S3c,d)
*Sycanus leucomesus* Walker, 1873: 84 [36], synonymized *Sycanus croceovittatus* by Hua, 1984: 16 [37]. **syn. nov.** for *S. bifidus* (Figure S3e,f)
**Chinese common name**: 黄带犀猎蝽

**Redescription. Coloration.** Body black, slightly shiny (Figure 2a, Figure 7, Figures S3 and S4). Apical half of corium of fore wing yellow or orange; membrane of fore wing pale brown, semitransparent; spot between ipsolateral eye and ocellus yellowish brown; each sternum of abdomen laterally with two round white spots (Figure 7, Figures S3 and S4). 

**Structure.** Body large-sized. Body mostly clothed with black long or short setae and procumbent short yellow setae; first antennal segment sparsely with long setae, second segment densely with short setae, third and fourth segments densely with procumbent short setae; anterior margin of fore wing with brown bent short setae; legs with black long setae; each segment of sterna of abdomen laterally with two white setae floccus. Anterior lobe of head sub-equal to posterior lobe in length, anteocular part distinctly shorter than postocular part; rostrum incurved, long and slender. Collar process indistinct; anterior pronotal lobe small, hemisphered and bulged, deeply depressed at base; posterior pronotal lobe rugose, lateral pronotal angle obtuse and round, posterior margin nearly straight, posterior angle nearly absent; scutellum with an erect thick spine, its apex bifid (Figures S3 and S4). Femora nearly of equal thickness, apical part somewhat thickened; fore wing extending beyond tip of abdomen. Fourth to sixth connexival segments of abdomen laterally expended (Figure 4a and Figure 7). Pygophore elliptic, median pygophore process laterally with two ear-shaped processes and medially with a large posteriorly produced process (Figure 8a,b); paramere clavate, apical half swelled with thick setae, middle part bent (Figure 8c,d). Phallobase triangular (Figure 8e); dorsal phallothecal sclerite widened laterally; endosoma medially with two weakly sclerotized stripe-shaped sclerites, apically with a well sclerotized horned process and laterally with six pairs of small spines (Figure 8f–i).

**Measurement [♂(n = 21)/♀(n = 19), in mm].** Body length 22.31–23.00/21.8–24.52; maximal width of abdomen 6.83–8.70/6.83–9.80. head length 5.25–5.41/5.36–6.14; length of anteocular part 1.84–1.94/2.00–2.21; length of postocular part 2.36–2.52/2.36–3.05; distance between ocelli 0.39–0.50/0.45–0.53; length of synthlipsis 0.79–0.89/0.84–0.95; length of antennal segments I–IV= 6.46–6.93/6.20–7.51, 2.21–2.52/2.26–2.36, 1.21–1.73/1.21–1.42, 7.35–7.46/7.88–9.19; length of visible rostral segments I–III = 2.52–2.68/2.84–3.36, 3.89–4.10/3.83–5.07, 0.74–0.79/0.74–0.89; length of anterior pronotal lobe 1.31–1.37/1.13–1.50; length of posterior pronotal lobe 2.31–2.78/2.63–3.57; maximal width of pronotum 4.31–4.78/4.73–6.46; length of scutellum 1.31–1.42/1.10–1.21; length of fore wing 13.39–15.12/14.18–16.80. 

**Type material. Syntype**, ♂, Hong Kong, “Habitat in China, Pflug”, No. ZMUC 00 103039. The antennae and the right foreleg are lost, and the apical spine of the scutellum is broken (ZMUC) (Figure 7). **Syntype**, ♂, Hong Kong, China, No. ZMUC 00 103040. The third and fourth segments of the left antenna; the left fore, mid, and hind legs; the right hind leg; and the abdomen are lost (ZMUC).

**Specimens examined. Syntype** of *Sycanus croceovittatus* Dohrn, 1859, ♀, China, 2542 (MfN) (Figure S4a,b); **Holotype** of *Sycanus villicus* Stål, 1863, ♀, Cambodia, BMNH(E)1255108, URL: https://data.nhm.ac.uk/object/9ac6a020-304f-4c2d-9cd5-7c0def47b4aa, accassed on 26 February 2024 (BMNH) (Figure S4c,d); **Holotype** of *Sycanus leucomesus* Walker, 1873, ♀, Burma, BMNH(E)1255110, URL: https://data.nhm.ac.uk/object/456b420e-6818-4b48-88c6-fa861fa18d76, accassed on 26 February 2024 (BMNH) (Figure S4e,f).

CHINA, Yunnan: 1♀, Mengyang, 800 m, 1991-VI-8, Cai Wanzhi leg. (CAU); 2♂, Menglun, 1991-V-18, Cai Wanzhi leg. (CAU); 1♀, Mengla, 1991-V-28, Cai Wanzhi leg. (CAU); 1♂, Menghai, 1991-V-30, Cai Wanzhi leg. (CAU); 1♀, Mengla, Yaoqu, 2005-V-9, Bai Xiaoshuan leg. (CAU); 1♂, 1♀, Wenshan, Malipo, 2005-VIII-17 (CAU); 4♂, 1♀, Lvchun, Huanglian mountain, 2012-V-10, Cai Wanzhi & Niu Xinwei leg. (CAU); 2♂, 1♀, Banna, Jinghong, Jinuo, 1053 m, 2021-VII-25, Chen Zhaoyang & Liu Qinpeng leg. (CAU); 1♀, Honghe, Jinping, Fenshuiling National Nature Reserve, 2012-IX-24 (CAU); 1♀, Honghe, Yuanjiang, 2014-VIII-18 (CAU); 1♂, Puer, Mojiang, Sinanjiang, Dashaba, 2005-IX-5 (CAU); 1♂, Yingpan Town, Fengqing County, Lincang, 2005-VI-20 (CAU); 1♂, 1♀, Wenshan, Malipo, 2005-VIII-17 (CAU); 2♂, 1♀, Banna, Jinghong, Jinuo, 1053 m, 2021-VII-25 (CAU); 6♂, 2♀, Hekou, 2011-V-20 (CAU); 1♂, Xishuangbanna, Mengla, Yaoqu, 2013-III-24 (CAU).

CHINA, Guangxi: 5♂, 8♀, Shangsi, Shiwandashanm, 2006-VIII-28, 1♂, 1♀, 2006-IX-2, Huang Xia leg. (CAU); 6♂, 4♀, Ningming, Longrui, 2006-V-13–22, Huang Xia & Shi Zhongting leg. (CAU); 1♂, Huaping, 1963-VI-6, Yang Jikun leg. (CAU); 2♀, Nanning, Fusui, 240 m, 2004-VIII-18/20, Zhang Kuiyan leg. (CAU); 2♂, Longzhou, Nonggang, 1982-V-19, 240 m, Wang Xinli leg. (CAU); 1♂, Jinxiu, 720 m, 1982-V-10, Wang Xinli leg. (CAU); 1♂, Ningming, Longrui, 180 m, 1984-V-25, Li Fasheng leg. (CAU); 1♂, Baise, Napo, Baisheng, 2020-V-26, Zhao Ping leg. (NNU); 5♂, 7♀, Baise, Napo, 2020-V-26 (NNU); 1♀, Nanning, Wuming, Daming Mountain, 2016-VIII-8–10, Zheng Yuchen leg. (CAU); 1♂, Baise, Leye, 1200 m, 2017-V-24 (CAU); 4♂, 4♀, Ningming, Longrui Nature Reserve, 2006-V-17, Huang Xia & Shi Zhongting leg. (CAU).; 1♀, Nanning, Fusui, 200 m, 2004-VIII-18, Zhang Kuiyan leg. (CAU); 5♂, 7♀, Baise, Napo, 2020-V-26 (NNU); 3♂, 1♀, Chongzuo, Longzhou, Zhubu, 2020-V-26 (NNU); 4♂, Fangchenggang, Shangsi, Shiwandashan, 2006-VIII-28, Huang Xia leg. (CAU). 

CHINA, Guangdong: 1♂, Meixian, 1981-IX-6 (CAU); 1 ♂, Yangjiang, Yangchun, Mashui, 2002-IV-30 (CAU); 1♂, Huizhou, 2004-VIII-19 (CAU); 1♂,Meixian, 1981-IX-6 (CAU). 

CHINA, Hong Kong: 2♀, 2019-V-21–22 (CAU). 

CHINA, Hainan: 1♂, 3♀, Baisha, Yinggeling, 2008-X-8, Zhang W.J. leg. (CAU); 1♀, Wuzhishan, Fanyang, Bulun, 2008-X-26, Zhang W.J. leg. (CAU); 3♂, Baisha, Nankai, Mohao, 2008-V-30, Zhang W. J. leg. (CAU); 1♂, Ledong, Jianfengling, 2007-V-10, Zhang W.J. leg. (CAU); 1♂, Baisha, Nankai, 2008-IV-28, Zhang W.J. leg. (CAU).

**Distribution.** China (Hong Kong, Guangdong <Baiyun Mountain, Gaoyao, Guangzhou, Meixian>, Guangxi <Fangcheng Fulong, Shangsi, Nanning, Napo, Ningming, Jinxiu, Longzhou, Huaping>, Yunnan <Jinping, Hekou, Xishuangbanna: Mengban, Menghai, Menglun, Mengyang, Mengla>, Guizhou <Maolan, Guiyang, Wangmo, Guiding>, Hunan, Fujian <Fuzhou, Hua’an>, Hainan<Jianfengling>); Myanmar, India, Indonesia, Vietnam, Malaysia? (Federated Malay States), Java, Borneo, Bengal, Thailand, Cambodia.

**Remark.** In *S. bifidus,* the posterior half of the corium is yellowish to reddish brown, and the pronotum is brown to black. The polymorphism of body coloration brings difficulties in the species identification of *S. bifidus*.

The two Syntype specimens of *S. bifidus* are deposited in ZMUC, one in Kiel, collected from Hong Kong, China [11,33]. We examined two Syntype specimens’ photographs of *S. bifidus* deposited in ZMUC (Figure 7). We also examined the Syntype specimen’s photograph of *S. croceovittatus* deposited in MfN (Figure S3a,b), which was described by Dohrn in 1859 based on five syntypes (♀/♂) collected from China [11]. The species name, *S. croceovittatus*, was used for many years as a common species in South China [9,10]. We thought that *S. croceovittatus* Dohrn, 1859, should be a synonym of *S. bifidus*.

We examined holotype specimen of *S. villicus* Stål, 1863 (Figure S3c,d), deposited in BMNH (URL: https://data.nhm.ac.uk/object/9ac6a020-304f-4c2d-9cd5-7c0def47b4aa, accassed on 26 February 2024). Though most of the thorax is yellowish brown (vs. in *S. bifidus*, where the pronotum is totally black), we thought that *S. villicus* should be a synonym of *S. bifidus* on the basis of the body shape and the body coloration.

Hua [37] treated *S. leucomesus* as a synonym of *S. croceovittatus.* After we examined the holotype specimen of *S. leucomesus* (Figure S3e,f), deposited in the BMNH (URL: https://data.nhm.ac.uk/object/456b420e-6818-4b48-88c6-fa861fa18d76, accassed on 26 February 2024)*,* we thought that *S. leucomesus* should be a synonym of *S. bifidus*.

**(2) *Sycanus collaris* (Fabricius, 1781)**
*Reduvius collaris* Fabricius, 1781: 380 [2]; Zimsen 1964: 339 [33].
*Cimex carbonarius* Gmelin, 1790: 2199 [34], synonymized by Stål, 1874: 28 [31].
*Zelus collaris*: Fabricius, 1803: 285 [35].
*Arilus collaris*: Burmeister, 1835: 229 [38].
*Sycanus collaris*: Amyot & Serville, 1843: 360 [1]; Distant, 1904: 351 [8]; Distant, 1910: 207 [39]; Maldonado-Capriles, 1990: 311 [3].
*Sycanus leucomesus* Walker, 1873: 84 [36], synonymized in part by Distant, 1903: 212 [40]; Maldonado-Capriles, 1990: 311 [3].
**Chinese common name**: 淡带犀猎蝽

**Redescription.** Body black; about apical half of corium (excluding apical angle) and basal margin of membrane stramineous; membrane bronzy; antennae black, basal and subapical annulations of first segment, subbasal annulation of second segment, and apex of rostrum castaneous. Head about as long as pronotum and scutellum together; antenae with first segment subequal in length to fore femora. Apical spine of scutellum long, a little obliquely erect, its apex distinctly and somewhat broadly bifid; abdomen strongly dilated on each side, especially at the third and fourth segments, the posterior angles of the third and fourth segments more or less acute. Body length 22.00–25.00 mm.

**Distribution.** China?; Philippines, Malay Archipelago, Malay Peninsula, Sri Lanka, Thailand, Malacca, Sarawak, India, Aaasam, Myanmar.

**Remark.** *Reduvius collaris* Fabricius, 1781, the type species of the genus *Sycanus*, was built based on specimens from India. In the world catalogue of the assassin bugs [3], *S. collaris* (Fabricius, 1781) has been recorded to be distributed within China [2,8]. Zimsen in 1964 [33] recorded the three type specimens of *Reduvius collaris* Fabricius, 1781, one specimen in London and two specimens in Kiel. We examined many Indian specimens of *S. collaris* deposited in BMNH. However, we could not confirm whether the identification was correct, and we did not find the type specimen of *S. collaris* in BMNH. We also tried to communicate with the museum in Kiel but failed to obtain the photographs of two type specimens in Kiel. The species is mainly distributed in Southeast and South Asian countries. We have doubts about the distribution of this species in China but have listed it herein. The redescription of *Sycanus collaris* (Fabricius, 1781) is based on Fabricius [2] and Distant [39].

**Figure 9:**
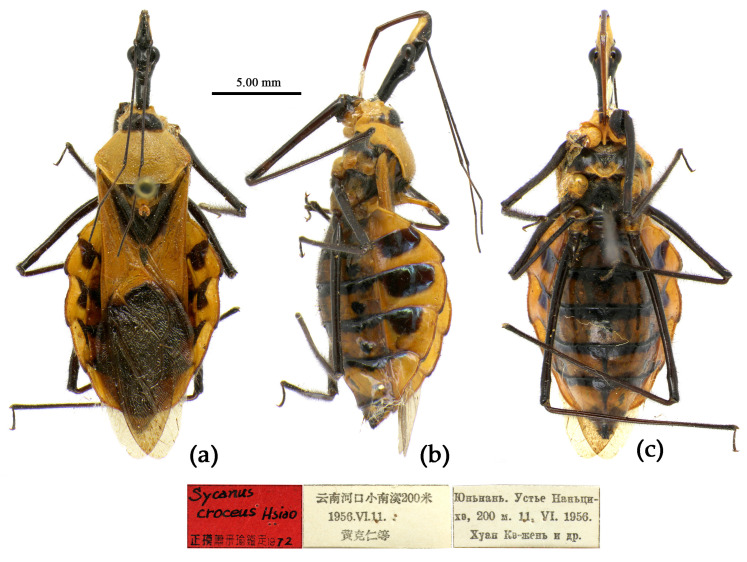
*Sycanus croceus* Hsiao, 1979, **Holotype**, habitus, female (IOZ). (**a**) dorsal view; (**b**) lateral view; (**c**) ventral view.

**Figure 10:**
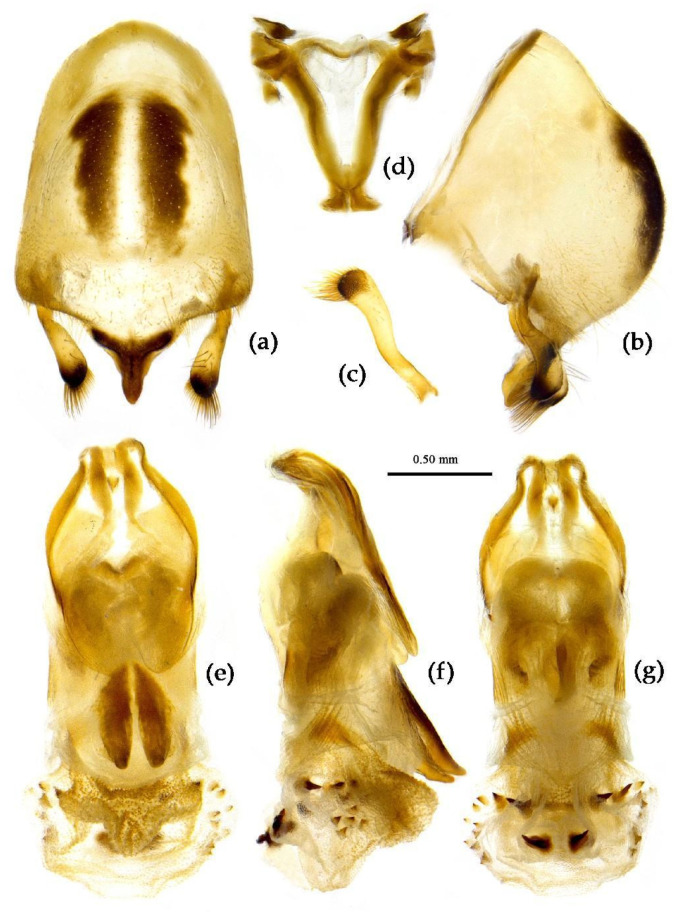
*Sycanus croceus* Hsiao, 1979, (**a**–**g**) male external genitalia; (**a**,**b**) pygophore with parameres; (**c**) paramere; (**d**) phallobase; (**e**–**g**) phallosoma; (**e**) dorsal view; (**b**,**f**) lateral view; (**a**,**g**) ventral view.

**(3) *Sycanus croceus* Hsiao, 1979**
(Figure 2b, Figure 3b, Figure 4b, Figure 5, Figure 9, Figure 10 and Figures S5)
*Sycanus croceus* Hsiao, 1979: 145 [9] (Figure 9); Hsiao & Ren, 1981: 520 [10]; Maldonado-Capriles, 1990: 311 [3]; Putshkov & Putshkov, 1996: 259 [11].
**Chinese common name**: 黄犀猎蝽

**Redescription. Coloration.** Body orange, with black markings (Figure 2b, Figure 5g, Figure 9 and Figure S5). Head (except ventral surface orange), antennae, eyes, anterior pronotal lobe, scutellum (except apical spine and posterior margin orange), clavus (except basal part yellow), inner side and apical angle of corium, leg (except coxae yellow), meso-sternum (except anterior and posterior margins yellow), meta-sternum, anterior half of meso- and meta-pleura, basal part of scutellum, inter-segmental stripes of abdomen, large spots of connexivum, discontinuous median longitudinal stripe and small round spots laterally on ventral surface of abdomen black; membrane of fore wing pale brown, semitransparent, with metallic-shiny; first visible rostral segment and basal part of second black, most of second and third brown.

**Structure.** Body median to large-sized. Body clothed with yellowish short setae; legs with erect long setae; thorax densely with short setae (Figure 9 and Figure S5). Postocular part of head longer than anteocular; antennae long and slender, first segment longest, third shortest, fourth slightly shorter than first; rostrum long, bent, first extending beyond middle part of eyes, second segment longest. Collar process with a small round tuber; anterior pronotal lobe small, hemisphere, basal part with short median longitudinal sulcus; posterior pronotal lobe rough, lateral angle round; posterior margin of scutellum round, apical spine sub-erect, apex bifid and posteriorly produced (Figure 3b, Figure 9a,b and Figure S5); legs slender; fore wing extending beyond tip of abdomen; fourth to sixth connexival segments roundly laterally dilated (Figure 4b, Figure 9 and Figure S5). Pygophore elliptic, median pygophore process “Y”-shaped (Figure 10a and Figure 10b); paramere clavate, apical part with thick setae, middle part slightly bent (Figure 10a–c). Phallobase triangular (Figure 10d); middle part of endosoma with two well sclerotized horned processes, apical part laterally with three pairs of larger spines and eight pairs of smaller spines (Figure 10e–g).

**Measurement [♂(n = 3)/♀(n = 3), in mm].** Body length 19.64–20.74/20.91–25.20; maximal width of abdomen 7.27/8.18. Head length 4.73/5.09–5.46; length of anteocular part 1.63–1.64/1.82–1.84; length of postocular part 2.36–2.52/2.55–2.84; distance between ocelli 0.47–0.55/0.47–0.55; length of synthlipsis 0.79/0.72–0.84; length of antennal segments I–IV = 6.73–6.83/6.18–7.30, 2.21/2.36–2.63, 1.64–1.94/1.64–2.10, 6.55/5.09–6.56; length of visible rostral segments I–III = 2.26–2.36/2.55–2.68, 3.41–3.64/3.04–4.04, 0.55–0.71/0.36–0.74; length of anterior pronotal lobe 1.21–1.27/1.27–1.37; length of posterior pronotal lobe 2.36–2.55/2.55–3.07; maximal width of pronotum 3.82–4.15/4.55–5.36; length of fore wing 13.64–13.65/14.18–17.06.

**Type material. Holotype**, ♀, CHINA, Yunnan, Hekou, Xiaonanxi, 200 m, 1956-VI-11, Huang Keren et al. Leg. (IOZ) (Figure 9). **Allotype**, same as Holotype, 1956-VI-10. **Paratype**, 1 ♀, CHINA, Guangxi, Baishou (IOZ); 1 ♀, Yunnan, Hekou (IOZ).

**Specimens examined.** CHINA, Guangxi: 1♂, 1♀, Jinxiu, 1983-VI-13 (NKU); 1♀, Longsheng, Huaping, 1983-VI-13 (NKU); 1♂, 1♀, Jinxiu, 800 m, 1990-VI-12, Li Xinzheng leg. (NKU); 1♀, Jinxiu, 2005-VII-24, Huang Xia leg. (CAU); 1♂, Laibin, Jinxiu, Dayaoshan, 1250 m, 2015-V-7 (CAU); 1♀, Jinxiu, Dayaoshan, Luoyingou, 1100 m, 2016-V-24, Zhao Jintang leg. (CAU); 2♀, Laibin, Jinxiu, Dayaoshan, 1100 m, 2016-V-31, Zhao Jintang leg. (CAU); 1♀ Jinxiu, Dayao Mountain, 1100 m, 2017-VII-20, Zhao J. T. leg. (CAU); 2♀, Jinxiu, Dayaoshan, 600 m, 2018-V-30 Zhao J. T. leg. (CAU); 1♂, 1♀, Longsheng, Huaping, 1963-V-1, Liu Sikong leg. (NKU); 1♂, Huaping, Cujiang, 1963-IV-8, Yang Jikun leg. (CAU); 1♀, Huaping, Tianping mountain, 1963-IV-5, Yang Jikun leg. (CAU); 1♂, 1♀, Guilin, Huaping, Tianping mountain, 1963-VI-5–8, Yang Jikun leg. (CAU); 1♂, Longsheng, 1992-V-23, Liu Guang leg. (CAU); 1♀, Jinxiu, 2005-VII-23, Zhao Ping leg. (CAU); 2♂, 2♀, Laibin, Jinxiu, Dayaoshan, 900 m, 2015-V-14, Lu Yanquan leg. (CAU); 1♂, Longzhou Nonggang, 2019-IV-22, He Zhuqing leg. (CAU); 1♀, Chongzuo, Longzhou, Nonggang Nature Reserve, 2020-V-27 (CAU); 2♀, Laibin, Jinxiu, Fenzhan, 810 m, 2020-VI-24 (CAU); 3♂, 5♀, Ningming, Huashan, 2014-VIII-13, 2020-VI (NKU); 1♂, 1♀, Baishou, 1957-VI-27 (NKU); 1♀, Xing’an, Jinshi, 2007-VII (CAU); 1♀, Baise, Jingxi, Bangliang, Renzhuan, 2010-VIII-1, Zhou S.Y. leg. (CAU); 1♂, Baise, Napo, 2020-V-26 (NNU); 3♀, Hezhou, Gupo mountain, 2011-VII (CAU).

CHINA, Yunnan: 1♂, 1♀, Honghe, Hekou, Nanxi, Huayudong, 200 m, 2016-IV-24, Wang Yutang & Yang Xiaodong leg. (CAU); 1♂, Xishuangbanna, Mengla, Mengxing, 1981-V-23, Yang Pingzhi leg. (CAU); 1♀, Xishuangbanna, Menghai, 1984-VI-21, Xiong Jiang leg. (CAU); 1♀, Menghai, 1984-VI-21, Xiong Jiang leg (CAU).; 1♂, Mengxing, 1981-V-23, Yang Pingzhi leg. (CAU); 1♂, Guangxi, Ren Shuzhi leg. (NKU).

CHINA, Guangdong: 2♀, Guangzhou, Conghua, Liuxihe, 2004-VI-20 (CAU); 1♀, Shixing, Chebaling Nature Reserve, 2003-IX-15 (CAU); 1♂, Nanling Nature Reserve, 2017-VI-18, Zhao Yisheng leg. (CAU); 1♀, Shaoguan, Wujiang, 2016-V-28, Ge Zhentai leg. (CAU); 1♂, 1♀, Shaoguan, Qujiang, Xiaokeng National Forest Park, 2013-V-1, Zheng Chaowu leg. (CAU); 1♂, 3♀, Shaoguan, Qujiang, Xiaokeng National Forest Park, 2013-X-1, Zheng Chaowu leg. (CAU); 3♂, 2♀, Nanling Nature Reserve, 2017-VI-18, Zhao Yisheng leg. (CAU).

CHINA, Jiangxi: 1♀, Ganzhou, Longnan, Hengkeng, Jiulian Mountain, 500 m, 2020-VII-05, Zheng Yuchen leg. (CAU); 1♂, 1♀, Ganzhou, Chongyi, Yangling National Forest Park, 2014-VI-18, Li Yanjing leg. (CAU).

CHINA, Guizhou: 1♂, Rongjiang, Pingyang, Xiaodanjiang, 2005-V-31, Song Yuehua leg. (CAU); 1♂, Rongjiang, Pingyang, 2005-VI-1, Song Yue Hua leg (CAU).

CHINA, Fujian: 2♂, 2♀, Quanzhou, Dehua, Shangyong, 2014-VI-25 (CAU); 1♀ Wuyi Mountain, 2010-VII-8, Luo X.Y. leg. (CAU); 1♂, 2♀, Quanzhou, Dehua, Chishui, 2014-VI-20 (CAU).

**Distribution.** China (Guizhou <Rongjiang>, Yunnan <Hekou Nanxi, Menghai, Mengxing>, Guangxi <Baishou, Jinxiu, Huaping, Rong’an, Longzhou, Ningming, Du’an, Longsheng, Napo, Fangcheng, Fulong, Longzhou Nonggang>, Guangdong <Conghua, Chebaling, Shaoguan, Nanling>, Jiangxi <Ganzhou>, Fujian (Fuzhou, Sanming, Longyan, Dehua, Hua’an, Yongding, Longqishan, Quanzhou);Vietnam.

**Figure 11:**
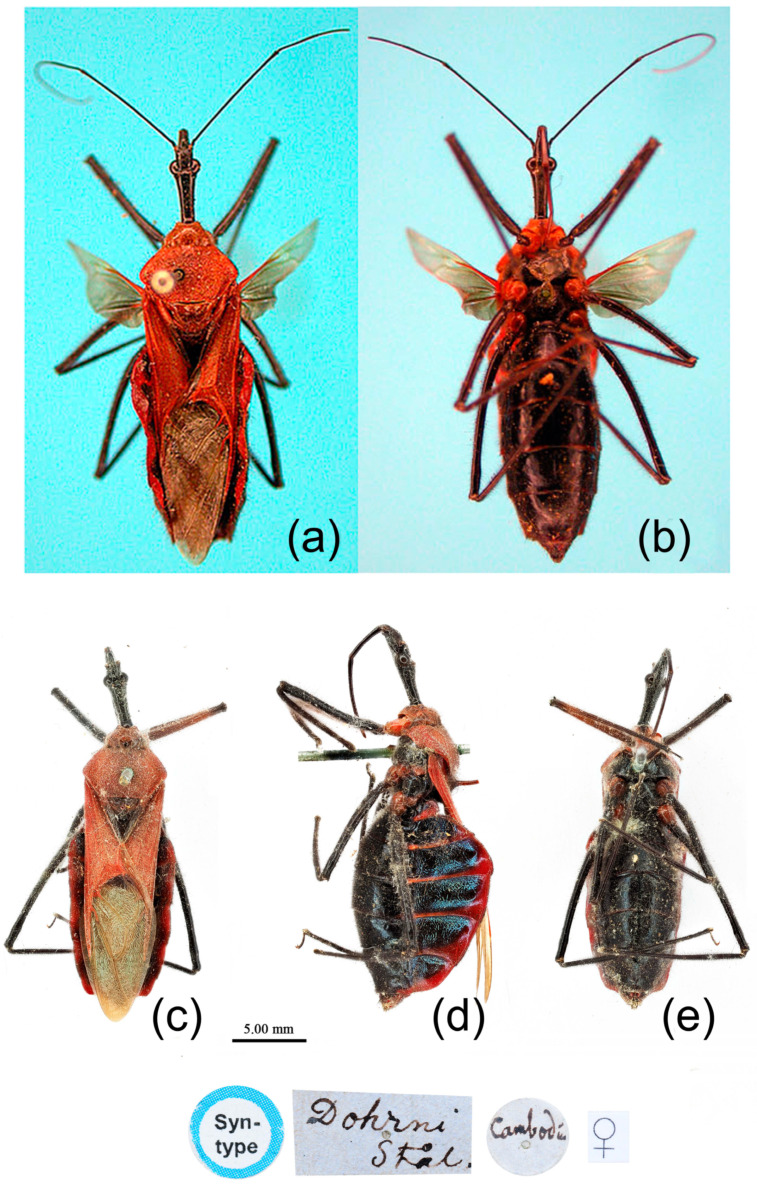
*Sycanus falleni* Stål, 1863, (**a**,**b**) **Type**, habitus, female (NRM); (**c**–**e**) **Syntype**, habitus, female (BMNH). (**a**,**c**) dorsal view; (**d**) lateral view; (**b**,**e**) ventral view.

**Figure 12:**
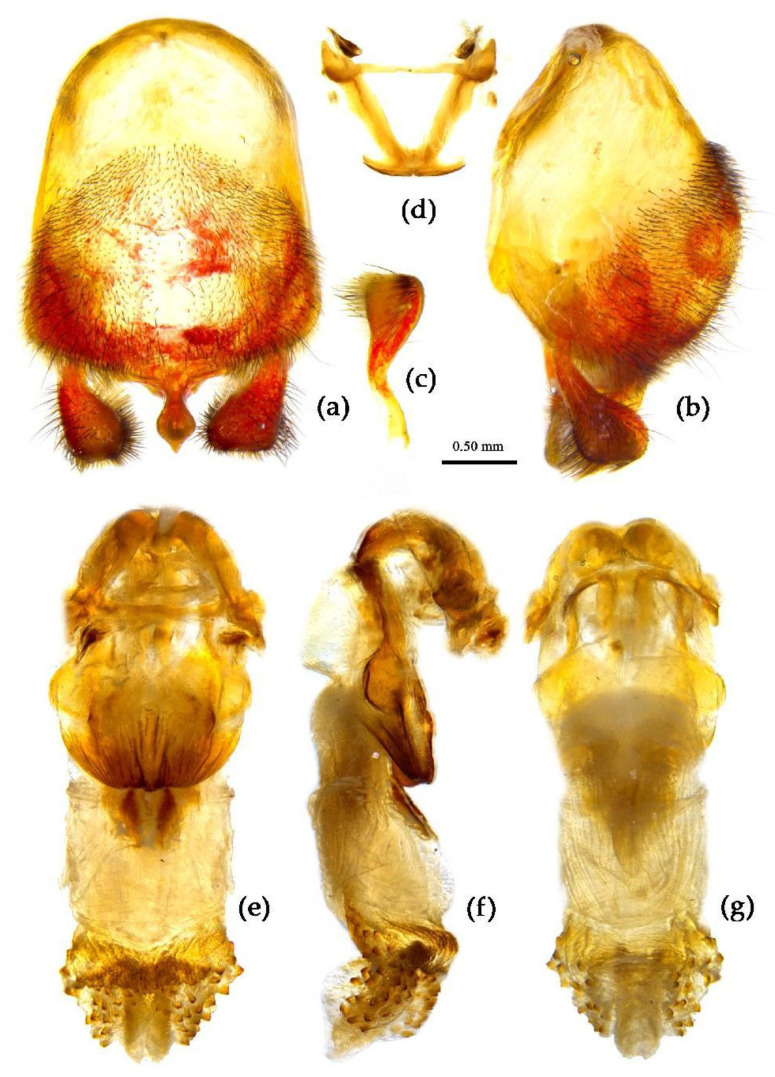
*Sycanus falleni* Stål, 1863, (**a**–**g**) male external genitalia, (**a**,**b**) pygophore with parameres, (**c**) paramere, (**d**) phallobase, (**e**–**g**) phallus, (**e**) dorsal view, (**b**,**f**) lateral view, (**a**,**g**) ventral view.

**(4) *Sycanus falleni* Stål, 1863**
(Figure 2c, Figure 3c, Figure 4c, Figure 6, Figure 11, Figure 12 and Figures S6, S12c, and S12d)
*Sycanus falleni* Stål, 1863: 34 [7] (Figure 11); Distant 1904: 354 [8]; Hsiao, 1979: 146 [9]; Hsiao & Ren, 1981: 520 [10]; Maldonado-Capriles, 1990: 311 [3]; Putshkov & Putshkov, 1996: 259 [11].
**Chinese common name**: 大红犀猎蝽

**Redescription. Coloration.** Body red, with black markings (Figure 2c, Figure 11 and Figure S6). Head, antennae, legs (except coxae red), rostrum (except apical part brown), most of meso- and meta-pleura (except posterior part red), sterna of meso- and meta-thorax, sterna and connexiva of abdomen (except inter-segmental stripe of abdomen yellow, and inter-segmental stripe and lateral margins of connexiva red), posterior half of clavus of fore wing, basal part of scutellum black and with blue shiny; pronotum and propleuron, prosternum (except middle part black), posterior half of meso- and meta-pleura, scutellum, coxae, inter-segmental stripe and lateral margins of connexivum, apical part of abdomen red; corium of fore wing red, clavus black (except basal part red), membrane pale brown and semitransparent (Figure 11 and Figure S6).

**Structure.** Body large-sized. Body clothed densely with yellowish short setae and procumbent short setae; legs with erect longer setae; thorax densely with short setae. Postocular part of head longer than anteocular; antennae long and thin, fourth segment longest and as long as first, third shortest; rostrum long, bent, first extending to middle part of eyes, second segment longest. Collar process round and indistinct; anterior pronotal lobe small, hemispherical, basally with short median longitudinal sulcus; posterior pronotal lobe rough, lateral angle round; posterior margin of scutellum round, apical spine erect, long, its apex bifid (Figure 3c, Figure 11b and Figure 11e); legs slender; fore wing extending beyond tip of abdomen; fourth to sixth segments of connexivum dilated laterally (Figure 4c and Figure 11). Pygophore elliptic, median pygophore process distinctly produced posteriorly (Figure 12a,b); paramere clavate, apical half swelled with thick setae, middle part bent (Figure 12a–c). Phallobase triangular, basal plate bridge thinner than basal plate (Figure 12d); dorsal phallothecal sclerite apically constricted; endosoma apically laterally with more than 30 pairs of small spines and medially with a pair of feebly sclerotized short stripe-shaped sclerites (Figure 12e–g).

**Measurement [♂(n = 10)/♀(n = 10), in mm].** Body length 23.63–24.46/24.94–26.62; maximal width of abdomen 6.56–8.66/8.14–8.40. Head length 5.46–5.51/5.51–6.20; length of anteocular part 1.79–1.84/2.10–2.15; length of postocular part 2.78/2.36–2.89; distance between ocelli 0.58/0.53–0.58; length of synthlipsis 0.84–0.89/0.89–1.00; length of antennal segments I–IV = 7.09–7.35/6.83–7.25, 2.52–2.57/2.52–2.63, 2.15–2.21/2.00–2.31, 7.35–7.61/6.83–7.35; length of visible rostral segments I–III = 2.63–2.78/2.84–3.05, 4.10–4.41/4.46–4.73, 0.84–0.89/0.79–0.89; length of anterior pronotal lobe1.31–1.37/1.50–1.58; length of posterior pronotal lobe 2.52–2.78/3.15–3.57; maximal width of pronotum 4.67–4.99/5.57–6.41; length of scutellum 1.42–1.47/1.47–1.58; length of fore wing 15.23–16.28/16.80. 

**Type material. Syntype**, ♀, Cambodia, (URL: http://www2.nrm.se/en/het_nrm/f/sycanus_falleni.html, accessed on 26 February 2024) (Figure 11a,b) (NRM); **Syntype**, ♀, Cambodia (Figure 11c–e) (BMNH).

**Specimens examined. Holotype** of *Sycanus ventralis* Distant, 1919, ♂, Tonkin, Hagiang, May-1914, R.V. de Salvaza leg., BMNH(E)1255105, URL: https://data.nhm.ac.uk/object/fd802d93-58b1-4c1e-a72c-49e6cae36788, accessed on 26 February 2024 (BMNH) (Figure S7a,b); **Holotype** of *Sycanus viduus* Distant, 1919, ♂, Luang Prabang, Ban Samang, 9-XI-1917, R.V. de Salvaza leg. URL: https://data.nhm.ac.uk/object/9ac6a020-304f-4c2d-9cd5-7c0def47b4aa, accessed on 26 February 2024 (BMNH) (Figure S7c,d).

CHINA, Guangxi: 10♀, 2♂, Ningming, Longrui, 2006-V-18, Huang Xia & Shi Zhongting leg. (CAU); 1♀, Ningming, Longrui, 1984-V-20, Lu Xiaolin & Wu Zheng Liangcai leg. (CAU); 2♀, Longrui, 1984-V-23, Ren Shuzhi leg. (NKU); 1♀, Pingxiang, Daqingshan (NKU); 1♂, 1♀, Pingxiang, 1964-VII-26, Wang Liangchen leg. (NKU); 1♀, Ningming, Longrui, 1984-V-18, Ren Shuzhi leg. (NKU).

CHINA, Yunnan: 1♂, Pingbian, Lincang, 1956-VI-27, 800–1300 m, Huang Keren leg. (NKU).

**Distribution.** China (Guangxi <Longsheng, Ningming, Pingxiang, Napo, Fangcheng Fulong, Longzhou Nonggang, Longrui>, Yunnan <Pingbian>); Myanmar, Cambodia, India, Vietnam.

**Remark.** The holotype specimen of *Sycanus ventralis* Distant, 1919 (Indochina) (BMNH) (Figure S7a,b), and the holotype specimen of *Sycanus viduus* Distant, 1919 (Indochina, Malaya) (BMNH) (Figure S7c,d), should be males of *Sycanus falleni* Stål, 1863. However, we did not dissect their male genitalia and compare their structural differences, so we did not treat them as two synonyms of *Sycanus fallen* Stål, 1863. Further research is needed to determine whether these two species are synonyms of *S. fallen.*

**Figure 13:**
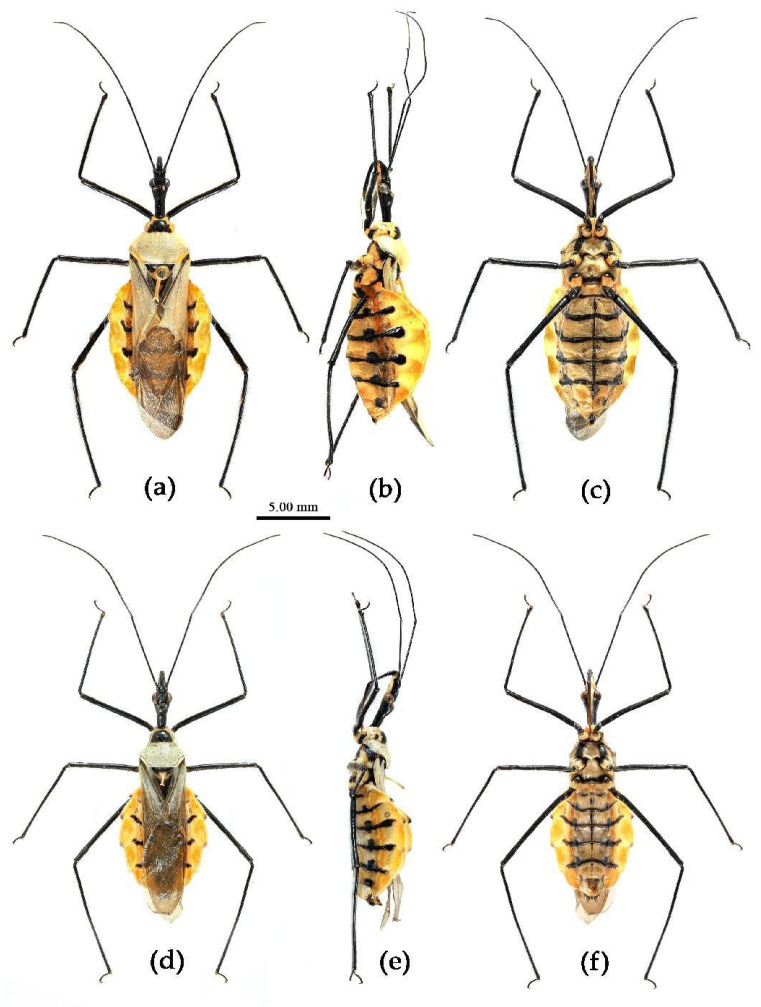
*Sycanus flavicorius* Li & Cai **sp. nov.**, habitus, (**a**–**c**) **Holotype**, female (CAU), (**d**–**f**) **Paratype**, male (CAU). (**a**,**d**) dorsal view; (**b**,**e**) lateral view; (**c**,**f**) ventral view.

**Figure 14:**
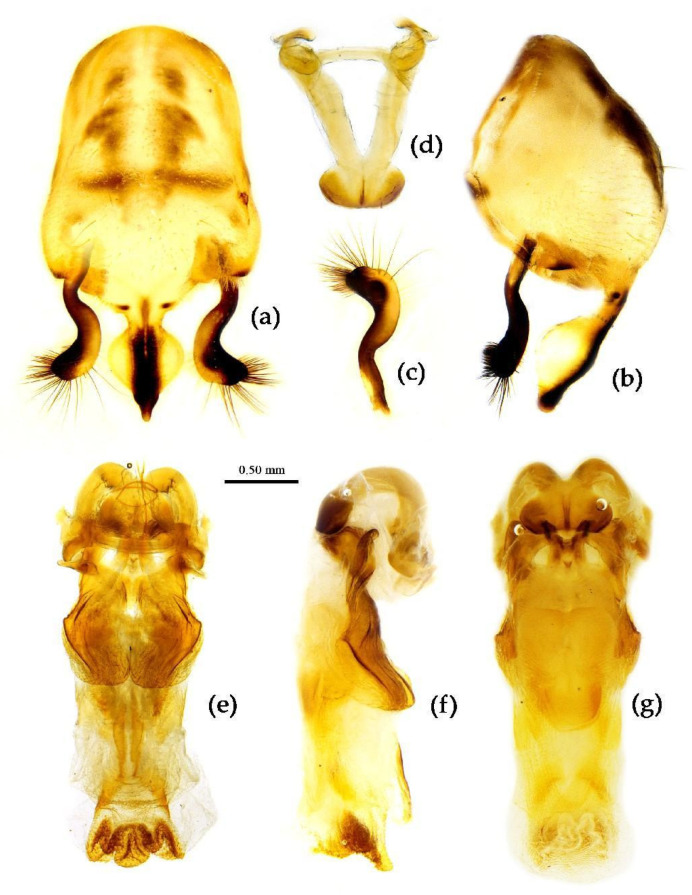
*Sycanus flavicorius* Li & Cai **sp. nov.**, (**a**–**g**) **Paratype**, male external genitalia, (**a**,**b**) pygophore with parameres, (**c**) paramere, (**d**) phallobase, (**e**–**g**) phallus, (**e**) dorsal view, (**b**,**f**) lateral view, (**a**,**g**) ventral view.

**(5) *Sycanus flavicorius* Li & Cai sp. nov.**
urn:lsid:zoobank.org:act:BB25EB72-46FC-4F46-9461-ED3588126F50
(Figure 2d, Figure 3d, Figure 4d, Figure 13 and Figure 14)
**Chinese common name**: 黄革犀猎蝽

**Diagnosis.** The new species resembles *S. croceus* in the body shape and coloration. However in the new species, the coxa of the leg is yellow, and its basal part is black; the sterna of meso- and meta-thoraxes are yellow, and the markings on lateral side of the sternum of the meso-thorax and posterior margin of the sternum of the meta-thorax are black. The black marking of the connexivum is smaller and only laterally extends to half of the connexivum or nearly reaches the lateral margin; the posterior pronotal lobe and the corium of the fore wing are yellowish white (vs. in *S. croceus*, where the coxa is yellow without black markings; the sterna of meso- and meta-thoraxes are black; the anterior and posterior margins of sterna of meso- and meta-thoraxes are yellow; the black marking of connexivum is larger, almost laterally extending to the lateral margin of the connexivum; and the posterior pronotal lobe and the corium of the fore wing are yellow). 

The male external genitalia of the new species is somewhat similar to that of *S. marginellus* among these Chinese *Sycanus* species, but there are great differences in the body coloration between the two species: the new species is yellowish with some black markings (vs. *S. marginellus*, which is black with red stripes). In addition, in new species, the pygophore is armed with a larger median process, the apical part of the lateral endosoma is without small spines (vs. in *S. marginellus*, where the median process of the pygophore is smaller and the apical part of endosoma is laterally armed with six pairs of small spines).

**Description. Coloration.** Body yellowish with black markings (Figure 2d and Figure 13). Head (except ventral surface yellowish), anterior pronotal lobe (except anterior and lateral margins yellowish), upper margin of pleura of meso- and meta-thoraxes, scutellum (except posterior margin and apical spine yellowish), round markings of two lateral sides, median longitudinal stripe and inter-segmental transversal stripes of ventral surface of abdomen, legs (except apical half of coxa yellowish), antennae, black; posterior pronotal lobe (except post-lateral margin yellowish), corium, clavus, epimeron of propleuron, sterna of thorax (except lateral margin of meso-sternum and anterior margin of meta-sternum black), sterna of abdomen (except black stripes), milk-white to yellowish white; ventral surface of head, coxa (except basal part black), anterior and lateral margins of anterior pronotal lobe, sternum of pro-thorax, episternum of propleuron, pleura of meso- and meta-thoraxes (except upper margin black), posterior margin and apical spine of scutellum, connexivum (except black markings) yellowish.

**Structure.** Body median-sized. Body clothed with white setae; legs clothed with longer erect setae. Head long, anteocular part shorter than postocular; ocelli separated, distance between ocelli wider than that between ipsolateral ocellus and eyes. Anterior pronotal lobe hemispherical basal median longitudinal sulcus short; posterior pronotal lobe rough with irregular wrinkles; lateral pronotal angle round; posterior margin of pronotum straight; apical spine of scutellum situated in middle part, thick and long, produced upwards then backward, apex bifid (Figure 3d and Figure 13). Fore wing extending beyond tip of abdomen. Abdomen laterally widely roundly dilated, especially fourth to sixth connexival segments, posterior-lateral angle of each connexival segment indistinct and round (Figure 4d and Figure 13). Pygophore elliptic, median pygophore process distinctly produced posteriorly (Figure 14a,b); paramere clavate, apical part with thick long setae, middle part bent (Figure 14a–c). Phallobase triangular, basal plate bridge thinner than basal plate (Figure 14d); dorsal phallothecal sclerite apically constricted; endosoma apically armed with many small processes, but laterally without small spines, subapical part with two pieces of narrow and long feebly sclerotized sclerites (Figure 14e–g).

**Measurement [Holotype ♀, ♂(n = 2)/♀(n = 1), in mm].** Body length 19.8, 17.27–17.28/19.87, maximal width of abdomen 5.82, 5.38–6.91/5.82; Head length 4.65, 4.36–4.55/4.65; length of anteocular part 1.47, 1.63–1.64/1.47; length of postocular part 2.03, 2.00/2.03; length of synthlipsis 0.73, 0.61–0.69/0.73; distance between ocelli 0.28, 0.27–0.36/0.28; length of antennal segments I–IV = 5.34, 5.09–5.27/5.34, 2.18, 2.00–2.10/2.18, 2.37, 2.00–2.18/2.37, 5.55, 5.09–5.45/5.55; length of visible rostral segments I–III = 2.56, 2.00/2.56, 3.13, 2.73–2.91/3.13, 0.73, 0.63–0.68/0.73; length of anterior pronotal lobe 1.14, 1.09/1.14; length of posterior pronotal lobe 2.33, 1.64–1.82/2.33; maximal width of pronotum 4.43, 3.27–3.45/4.43; length of scutellum 1.61, 1.27/1.61; length of fore wing 14.22, 11.27–11.45/14.22.

**Type material. Holotype**, ♀, CHINA, Yunnan, Lancang, Qianliuyizu town, Tianba village, 1375 m, 2017-VII-20, Zhou Zhen & Leo Xiaolong leg. (CAU) (Figure 13a–c). **Paratypes**, 1♂, 1♀, CHINA, Yunnan, Lancang, Gengma, 600 m, 2019-VII (CAU)(Figure 13d–f); 1♂, CHINA, Yunnan, Puer, 2022-VII-4, Zhang Guirong leg. (NNU).

**Etymology.** The species name alludes to the flavus corium of the fore wing. The Latin noun flavus means “yellow”.

**Distribution.** China (Yunnan<Lancang, Puer>).

**Figure 15:**
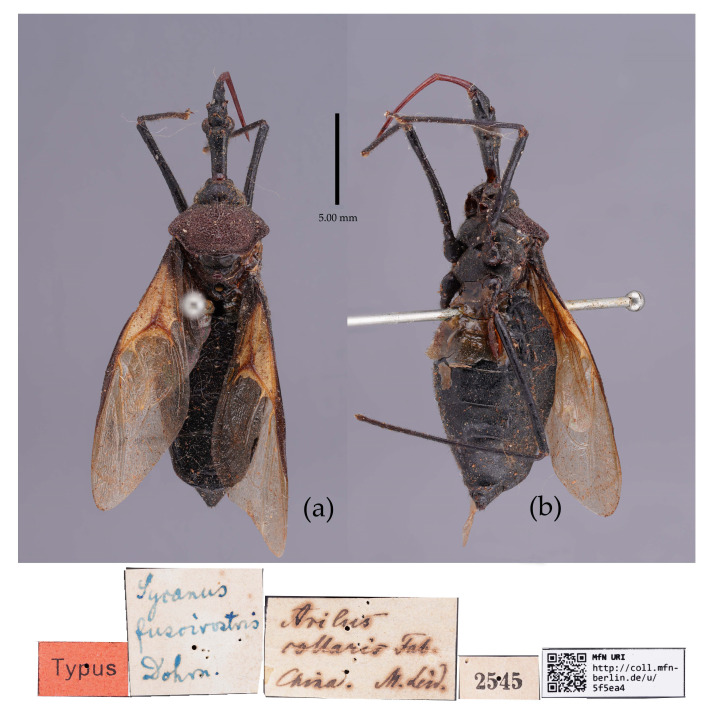
*Sycanus fuscirostris* Dohrn, 1859, **Syntype**, female (MfN), habitus, collected from China, 2545. (**a**) dorsal view; (**b**) lateral view.

**(6) *Sycanus fuscirostris* Dohrn, 1859**
(Figure 15)
*Sycanus fuscirostris* Dohrn, 1859: 99 [6] (Figure 15); Maldonado-Capriles, 1990: 312 [3]; Putshkov & Putshkov, 1996: 259 [11].
**Chinese common name**: 黄翅犀猎蝽

**Redescription. Coloration.** Body black, not shiny (Figure 15). Corium of fore wing yellowish white (except basal part and anterior margin black), vein of corium brown, membrane semitransparent. Rostrum brown, shiny.

**Structure.** Body large-sized, 23.00 mm (Figure 15). Body clothed with brown procumbent short setae; legs with black suberect setae. Anterior lobe of head sub-equal to posterior lobe, anteocular part distinctly shorter than postocular part; rostrum incurved, slender. Collar process indistinct; anterior pronotal lobe small, hemisphered and bulged, deeply depressed at base; posterior pronotal lobe rugose, lateral pronotal angle round, posterior margin nearly straight, posterior angle nearly absent; apical spine of scutellum lost. Fore wing narrow. Abdomen laterally expended (Figure 15).

**Type material. Holotype**, ♀, collected from China, 2545. The antennae, foreleg, right hind leg, and apical spine of the scutellum are lost (Figure 15) (NRM).

**Distribution.** China.

**Remark.** We examined the type specimens of *S. fuscirostris* Dohrn, 1859 (Figure 15). The holotype of the species was one female, collected from China, and deposited in MfN [6,11]. We did not find any other Chinese specimens of this species. *S. fuscirostris* is very similar to *S. bifidus* in body shape and coloration. We redescribed this species based on the holotype photograph of *S. fuscirostris* Dohrn, 1859 (Figure 15). In *S. fuscirostris*, most of the corium is yellow, and just the basal part is black, the veins of the corium is brown (vs. in *S. bifidus*, where the corium is black, its apical half is yellow, and its veins are not brown). However, we hypothesized *S. fuscirostris* Dohrn, 1859 may be another synonym of *S. bifidus* (Fabricius, 1787). The relationship between these two species needs further research.

**Figure 16:**
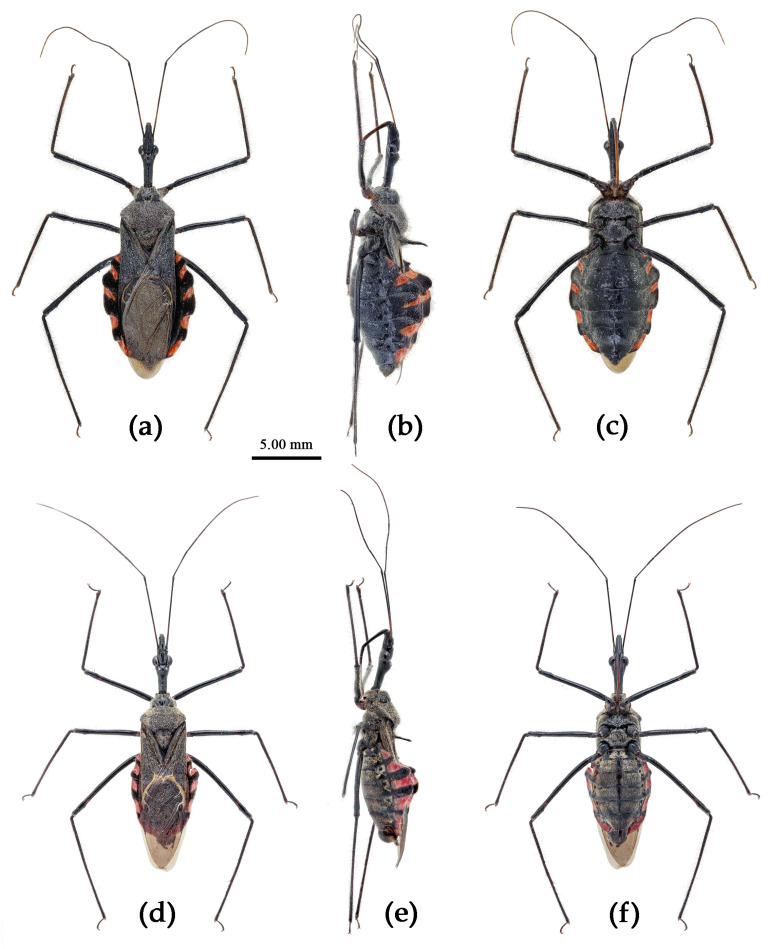
*Sycanus hainanensis* Wang & Cai **sp. nov.**, (**a**–**c**) **Holotype**, female (CAU); (**d**–**f**) **Paratype**, male (CAU); (**a**,**d**) dorsal view; (**b**,**e**) lateral view; (**c**,**f**) ventral view.

**Figure 17:**
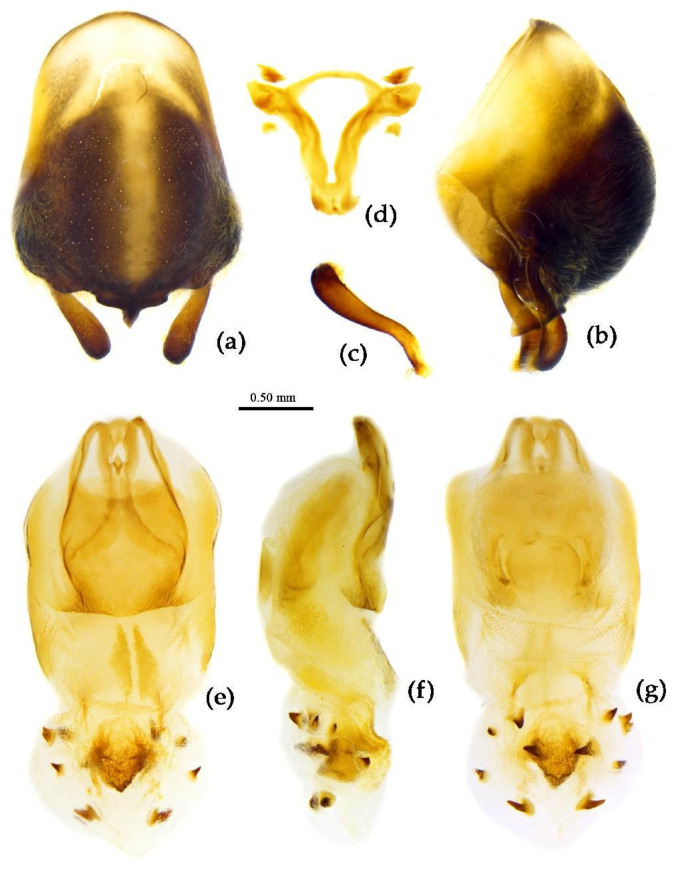
*Sycanus hainanensis* Wang & Cai **sp. nov.**, (**a**–**g**) male external genitalia; (**a**,**b**) pygophore with parameres; (**c**) paramere; (**d**) phallobase; (**e**–**g**) phallosoma; (**e**) dorsal view; (**b**,**f**) lateral view; (**a**,**g**) ventral view.

**(7) *Sycanus hainanensis* Wang & Cai sp. nov.**
urn:lsid:zoobank.org:act:0A5B2FB7-BF18-41B2-9BA1-75D9C60C8EBD
(Figure 2e, Figure 3e, Figure 4e, Figure 16 and Figure 17)
**Chinese common name**: 海南犀猎蝽

**Diagnosis.** The new species is similar to *S. sichuanensis* in body shape and coloration. However, in the new species, the posterior margin of the corium is milk-white to yellowish brown (Figure 16) (vs. in *S. sichuanensis*, where most of the corium is milk-white to yellowish white). Through the molecular analyses based on COI DNA barcodes, the genetic distance between the two species (p-distance) was 9.64% (>2%) [13], which supports them as the two independent valid species (Table S2). 

**Description. Coloration.** Body black, with red markings (Figure 2e and Figure 16). Connexivum black, transversal markings of each segment red; posterior margin of corium of fore wing yellowish brown, membrane pale brown and semitransparent.

**Structure.** Body median-sized. Head, thorax, ventral surface of abdomen, corium of fore wing clothed with procumbent short white setae; legs with pale vertical setae of different length. Anteocular part distinctly shorter than postocular; rostrum in-curved, long and slender. Collar process indistinct, tuber-shaped; anterior pronotal lobe small, hemispherical and bulged; posterior pronotal lobe rugose, lateral angle obtuse and round, posterior margin nearly straight; scutellum sub-apically with a erect long spine, apex of spine bifid (Figure 3e and Figure 16). Femora nearly of equal thickness, apical part somewhat thickened; fore wing extending beyond tip of abdomen. Fourth and fifth connexival segments of abdomen laterally distinctly expended (Figure 4e and Figure 16). Pygophore elliptic, median pygophore process “T”-shaped (Figure 17a,b); paramere clavate, sub-basal part somewhat bent, apical part with short setae (Figure 17a–c). Phallobase triangular (Figure 17d); dorsal phallothecal sclerite sclerotized, tongue-shaped; endosoma medially with two very feebly sclerotized stripe shaped sclerites, apical part with a feebly sclerotized horned process and laterally with six pairs of small spines (Figure 17e–g).

**Measurement [Holotype ♀, ♂(n = 3)/♀(n = 7), in mm].** Body length 19.63, 16.38–16.82/18.62–19.63; maximal width of abdomen 7.09, 4.61–4.94/5.22–7.45; head length 5.27, 4.25–4.30/4.63–5.27; length of anteocular part 1.64, 1.55–1.56/1.51–1.73; length of postocular part 2.18, 1.99–2.01/2.18–2.27; distance between ocelli 0.36, 0.31–0.38/0.31–0.39; length of synthlipsis 0.55, 0.56–0.57/0.52–0.55; length of antennal segments I–IV= 6.18, 5.19–5.24/5.18–6.18, 2.00, 1.80–2.05/1.90–2.09, 1.27, 1.07–1.18/1.16–1.37, 6.30, 6.05–6.87/6.00–6.30; length of visible rostral segments I–III= 2.36, 1.81–1.86/1.94–2.55, 3.82, 2.76–3.24/3.31–3.82, 0.55, 0.36–0.41/0.42–0.55; length of anterior pronotal lobe 1.09, 0.76–0.86/1.00–1.09; length of posterior pronotal lobe 2.18, 1.54–1.71/1.89–2.18; maximal width of pronotum 4.18, 3.23–3.29/3.83–4.18; length of scutellum 1.27, 1.13–1.24/1.27–1.34; length of fore wing 12.18, 10.39–10.59/11.85–12.73. 

**Type material. Holotype**, ♀, CHINA, Hainan, Ledong, Jianfengling, Yulin Valley, 680 m, 2022-V-11, Zheng Yuchen leg. (CAU)) (Figure 16a–c). **Paratypes**, 3♂ (Figure 16d–f), 3♀ (CAU), 2♂, 2♀ (MfN), 2♂, 2♀ (ZMUC), 10♂, 10♀ (CATAS), CHINA, Hainan, Jianfengling, 2022-V-13, Wang Jianyun & Liu Yinyi leg.; 2♀, CHINA, Hainan, Diaoluoshan, Mengshuichang, 1981-IX-5 (CAU); 1♀, CHINA, Hainan, Wanning, Shimei, 1981-VI-10 (CAU). 

**Etymology.** The specific name *hainanensis* alludes to the type locality in Hainan province, China.

**Distribution.** China (Hainan).

**Figure 18:**
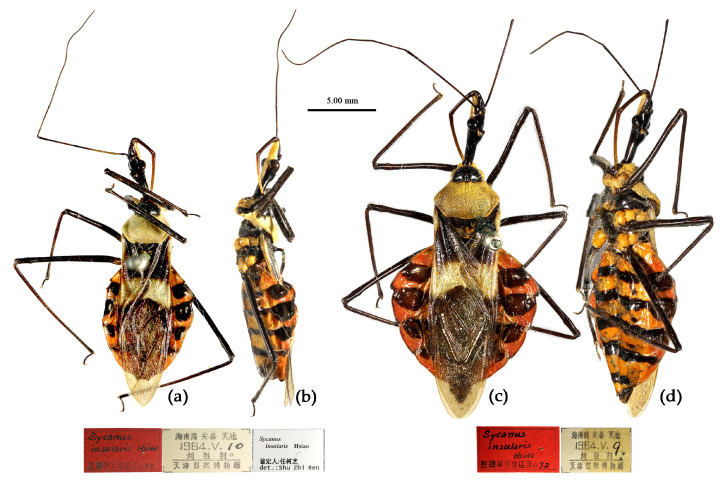
*Sycanus insularis* Hsiao, 1979, (**a**,**b**) male (NKU), **Holotype**; (**c**,**d**) female (NKU), **Allotype**, habitus; (**a**,**c**) dorsal view; (**b**,**d**) lateral view.

**Figure 19:**
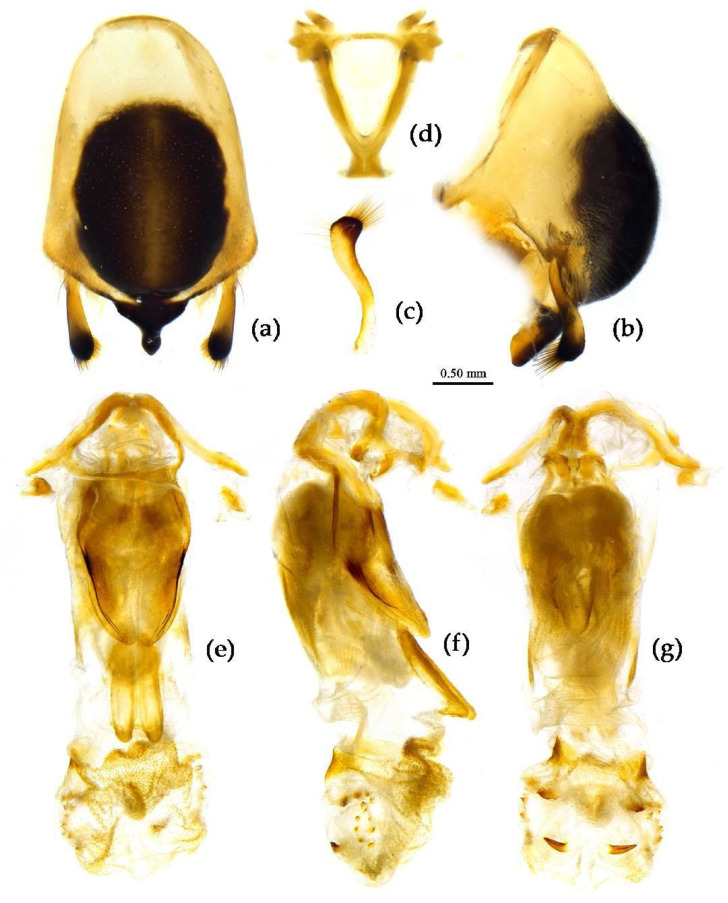
*Sycanus insularis* Hsiao, 1979, (**a**–**g**) male external genitalia; (**a**,**b**) pygophore with parameres; (**c**) paramere; (**d**) phallobase; (**e**–**g**) phallus; (**e**) dorsal view; (**b**,**f**) lateral view; (**a**,**g**) ventral view.

**Figure 20:**
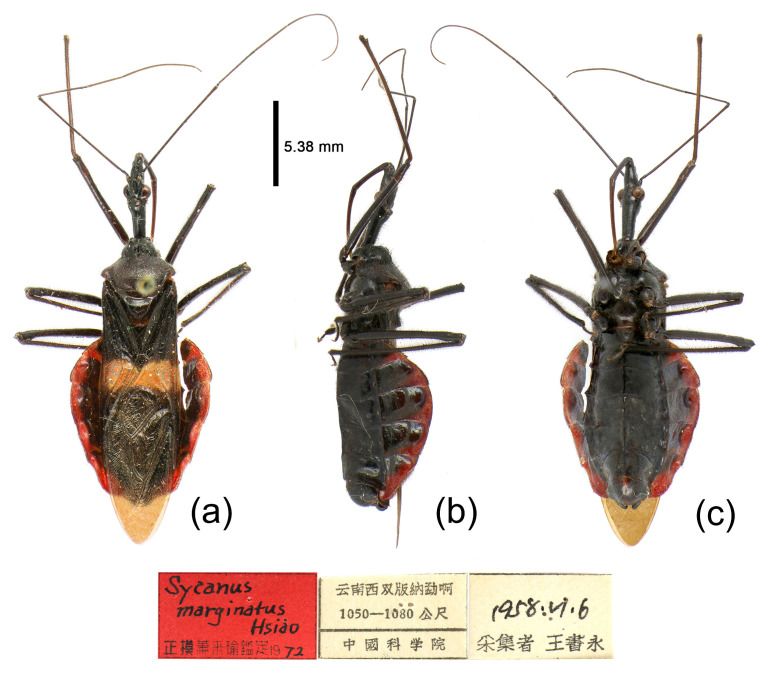
*Sycanus marginellus* Putshkov, 1987, **Holotype** of *Sycanus marginatus* Hsiao, 1979 (preoccupied), male (IOZ). (**a**) dorsal view; (**b**) lateral view; (**c**) ventral view.

**(8) *Sycanus insularis* Hsiao, 1979**
(Figure 2f, Figure 3f, Figure 4f, Figure 18 and Figure 19)
*Sycanus insularis* Hsiao, 1979: 146, 154 [9] (Figure 20a,b); Hsiao & Ren, 1981: 521 [10]; Maldonado-Capriles, 1990: 312 [3]; Putshkov & Putshkov, 1996: 259 [11].
**Chinese common name**: 黄背犀猎蝽

**Redescription. Coloration.** Body black, with yellowish and orange markings (Figure 2f and Figure 18). Head (except ventral surface yellow and apical part of rostrum pale brown), antennae (except two median annular markings of first segment brown), legs (except coxae orange), anterior pronotal lobe, episterna of pleura of meso- and meta-thoraxes, sterna of thorax, inter-segmental transversal stripe of abdomen and its expending round markings of connexivum, basal half and apical angle of corium of fore wing black; pronotum (except anterior lobe), propleuron, anterior margin of corium, apical half of corium (except apical angle) yellowish; coxae, abdomen (except black inter-segmental stripes), epimera of pleura of meso- and meta-thoraxes orange (Figure 2f and Figure 18).

**Structure**. Body median to large sized. Body clothed with white short setae; legs, pronotum, scutellum with erect long setae. Head longer, postocular part of head longer than anteocular; antennae slender and thin, first segment longest, and as long as fourth, third shortest and a little shorter than second; rostrum long, bent, first segment extending to middle part of eyes, second segment longest. Collar process round and indistinct; anterior pronotal lobe small, hemisphere, posterior part with short longitudinal sulcus; posterior pronotal lobe rough, lateral angle round; posterior margin of scutellum round, apical spine sub-erect, its apex bifid (Figure 3f and Figure 18); legs slender; fore wing extending beyond tip of abdomen; fourth and fifth connexival segments dilated laterally (Figure 4f and Figure 18). Pygophore elliptic, median pygophore process “Y”-shaped (Figure 19a,b); paramere clavate, apical half swelled with thick setae, middle part somewhat bent (Figure 19c). Phallobase triangular (Figure 19d); phallosoma elliptic; dorsal phallothecal sclerite sclerotized, tongue-shaped; endosoma medially with a pair of sclerotized horned processes, apically with a feebly sclerotized horned process, and laterally with two pairs of larger spines and 11 pairs of small spines (Figure 19e–g).

**Measurement [♂ (n=4)/♀ (n=3), in mm].** Body length 19.55/23.71–24.85, maximal width of abdomen 7.70/10.26–10.83; Head length 4.85/5.13–5.42; length of anteocular part 1.71/ 1.82–2.00; length of postocular part 2.28/2.28–2.85; length of synthlipsis 0.68/0.80–0.86; distance between ocelli 0.46/0.46–0.57; length of antennal segments I–IV = 6.16/6.84–7.13, 2.28/2.57–2.68, 1.71/1.82, 6.10/8.55–6.27; length of visible rostral segments I–III = 2.28/2.74–2.85, 3.14/3.82–3.93, 0.68/0.68–0.80; length of anterior pronotal lobe 1.14/1.20–1.31; length of posterior pronotal lobe 2.17/2.57–3.02; maximal width of pronotum 3.88/4.56–5.13; length of scutellum 1.37/1.43–1.82; length of fore wing 12.08/15.39–16.82. 

**Type material. Holotype**, ♂, CHINA, Hainan, Jianfengling, Tianchi, 1964-V-10, Liu Shengli leg (NKU) (Figure 18a,b). **Allotype**, ♀, same as Holotype, 1964-V-9 (NKU) (Figure 18c,d). **Paratype**, 3♀, same as holotype (deposited in NKU, NOT in TJNHM).

**Specimens examined.** CHINA, Hainan: 1♀, Jianfengling Nature Reserve, 900 m, 1980-IV-10, Xiong Jiang leg.; 1♂, 2♀, 2023-IV-21, Zhao Ping leg. (CAU); 1♀, Jianfengling Nature Reserve, 1982-IV-10, Liu Yuanfu leg. (CAU); 1♂, 2♀, Ledong, Jianfengling, Tianchi, 2015-V-07, Cai Nanyi leg. (CAU); 1♀, Ledong County, Xingfengling, Fengming Valley, 2015-IV-2, Lu Qiu leg. (CAU); 1♂, Ledong, Main peak of Jianfengling, 1412 m, 2019-IV-14–16, Song Haitian leg. (CAU); 1♀, Ledong, Jianfengling, 2013-IV-12, Sun & Zhang leg. (CAU); 1♀, Jianfeng mountain, 1982-VI-10, Liu Yuanfu leg. (CAU); 1♂, 1♀, Jianfengling (CAU); 1♀, Lingshui County, Diaoluoshan, 1981-V-6 (CAU); 1♀, Lingshui County, Diaoluoshan, 2009-IV-9–12, Hou Xiaohui leg., by light trap (CAU); 1♂, 1♀, Wuzhishan Nature Reserve, 2023-IV-18, Liu Xingyue leg. (CAU).

**Distribution.** China (Hainan <Jianfengling, Diaoluoshan, Wuzhishan>).

**Figure 21:**
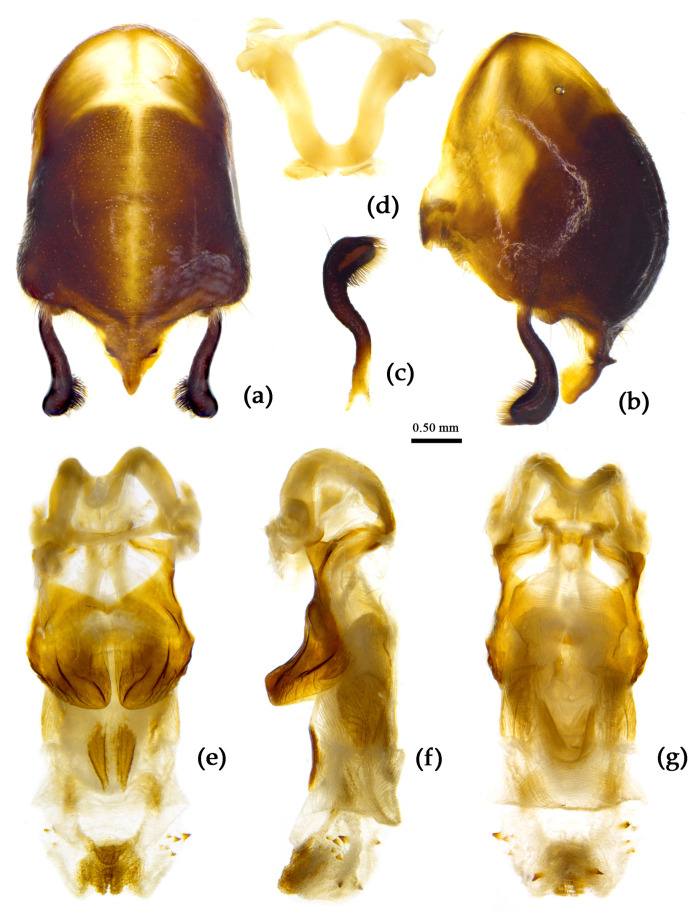
*Sycanus marginellus* Putshkov, 1987, (**a**–**g**) male external genitalia, (**a**,**b**) pygophore with parameres, (**c**) paramere, (**d**) phallobase, (**e**–**g**) phallus, (**e**) dorsal view, (**b**,**f**) lateral view, (**a**,**g**) ventral view.

**(9) *Sycanus marginellus* Putshkov, 1987**
(Figure 2g, Figure 3g, Figure 4g, Figure 20, Figure 21 and Figures S8)
*Sycanus marginatus* Hsiao, 1979: 143, 154 [9] (Figure 16). Preoccupied by *Sycanus marginatus* Walker, 1873 [36].
*Sycanus marginellus* Putshkov, 1987, Putshkov, Putshkov & Štys, 1987: 104 [29], new name for *S. marginatus* Hsiao, 1979; Putshkov & Putshkov, 1996: 259 [11].
*Sycanus hsiaoi* Maldonado-Capriles, 1990: 312 [3], new name for *S. marginatus* Hsiao, 1979. **syn. nov.**
**Chinese common name**: 赭缘犀猎蝽

**Redescription. Coloration.** Body black. Apical half of corium of fore wing (except apical angle of corium) yellowish; outer margin of connexivum red; apical part of rostrum brown (Figure 2g, Figure 20 and Figure S8). 

**Structure.** Body large-sized. Body clothed with black short setae; legs, pronotum, scutellum with longer vertical setae. Head longer, postocular part of head longer than anteocular; antennae slender and thin, fourth segment longest and as long as first, third shortest and a little shorter than second; rostrum long, bent, first extending to middle part of eyes, second segment longest. Collar process round; anterior pronotal lobe small, hemisphere, middle part basally with short longitudinal sulcus; posterior pronotal lobe rough, lateral angle round; posterior margin of scutellum round, sub-apically with a pyramidal process (Figure 3g, Figure 20 and Figure S8); legs slender; fore wing extending beyond tip of abdomen; fourth to sixth connexival segments roundly laterally ampliated (Figure 4g, Figure 20 and Figure S8). Pygophore elliptic, median pygophore process distinctly produced posteriorly (Figure 21a,b); paramere clavate, apical half swelled with thick setae, middle part distinctly bent (Figure 21c). Phallobase triangular, basal plate bridge thinner than basal plate (Figure 21d); phallosoma elliptic; dorsal phallothecal sclerite apically constricted; endosoma medially with two feebly smaller sclerotized stripe-shaped sclerites, apical part laterally with six pairs of small spines and medially with many tiny processes (Figure 21e–g).

**Measurement [♂(n = 10)/♀(n = 10), in mm].** Body length 21.27–25.08/26.11–27.27, maximal width of abdomen 8.18–8.55/9.92–10.91; Head length 4.73–5.53/5.70–5.82; length of anteocular part 1.63–1.88/2.00; length of postocular part 2.18–2.74/2.73–2.74; length of synthlipsis 0.64/0.91; distance between ocelli 0.27/0.33–0.51; length of antennal segments I–IV = 5.82/6.91–6.95, 2.36/2.96–3.09, 2.85/2.28, 6.55/9.09; length of visible rostral segments I–III = 2.18–2.85/2.91–2.96, 3.27–4.39/4.10–4.18, 0.64–0.74/0.80–0.91; length of anterior pronotal lobe 1.09–1.31/1.27–1.43; length of posterior pronotal lobe 2.00–2.85/3.02–3.09; maximal width of pronotum 3.64–4.85/5.42–5.45; length of scutellum 1.43/1.71; length of fore wing 13.09–16.53/17.67–18.18. 

**Type material. Holotype** of *Sycanus marginatus* Hsiao, 1979, ♀, CHINA, Yunnan, Xishuangbanna, Meng’a, 1050–1080 m, 1958-VI-6, Wang Shuyong leg. (IOZ) (Figure 20).

**Specimens examined.** CHINA, Yunnan: 1♂, 1♀, Menghai, 600 m, 20-V-1991, Cai Wanzhi leg. (CAU); 30♂, 30♀, Puer, Simao district, Nanping, Baizhi tamper, Wanmu tea Garden, 2022-VI-15, Zhang Guirong leg. (NNU).

**Distribution.** China (Yunnan<Lancang, Xishuangbanna: Menga, Menghai, Mengzha, Puer>).

**Remark.** Stål in 1874 [31] assigned *S. stali* Dohrn, 1859 (Figure S9a–d), and *S. generosus* Stål, 1863 (Figure S9e,f), as two variants of *S. stali*. We found the images of the syntype of *S. generosus* Stål, 1863 [7], deposited in NRM, URL: http://www2.nrm.se/en/het_nrm/g/sycanus_generosus.html, accessed on 26 February 2024 (Figure S9e,f). The type specimen’s photograph of *S. marginatus* Hsiao, 1979 (Figure 20), is morphologically similar to the type photograph of *S. generosus* Stål, 1863, based on the following characters: the general body shape, the black body coloration, the red outer margin of abdominal connexivum, and the posterior half of the corium with yellow markings. We have examined the type specimens’ photographs of *S*. *stali* deposited in MfN (Figure S9a–d) [6]. *S. generosus* resembles *S. stali* in the general body shape, the black body coloration, the coloration of the rostrum and antennae, and the yellow markings of the corium. However, we thought that the two species are maybe two independent valid species because in *S. generosus*, the outer margin of abdominal connexivum is red (vs. in *S. stali*, where the posterior margin of the third to fifth connexival segments are red). We did not dissect their genitalia to compare their differences. Therefore, we used *S. marginellus* Putshkov, 1987, as this species name and proposed that *S. hsiaoi* Maldonado-Capriles, 1990, is one **new synonym**.

**Figure 22:**
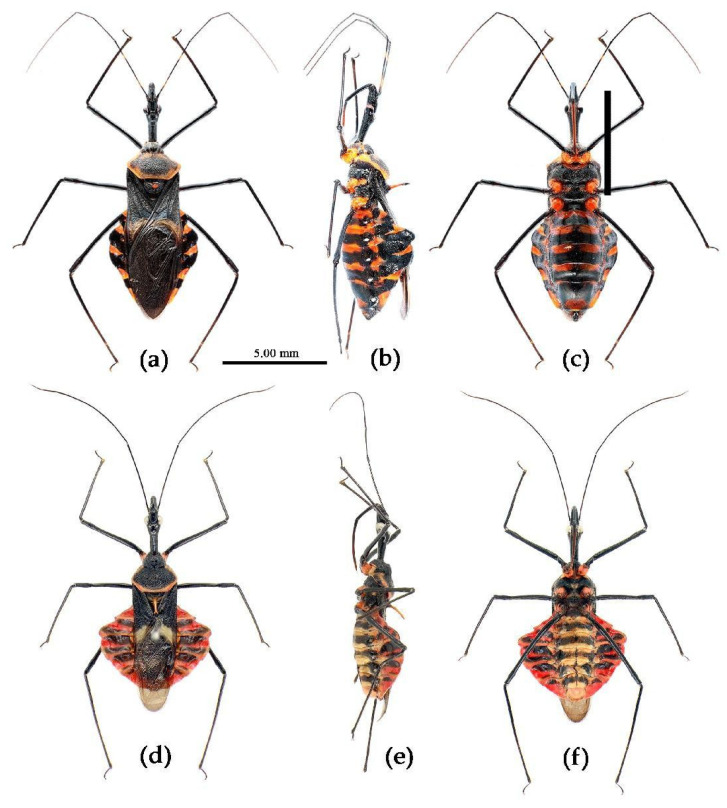
*Sycanus minor* Hsiao, 1979, habitus, (**a**–**c**) female, (**d**–**f**) males, (**a**,**d**) dorsal view, (**b**,**e**) lateral view, (**c**,**f**) ventral view.

**Figure 23:**
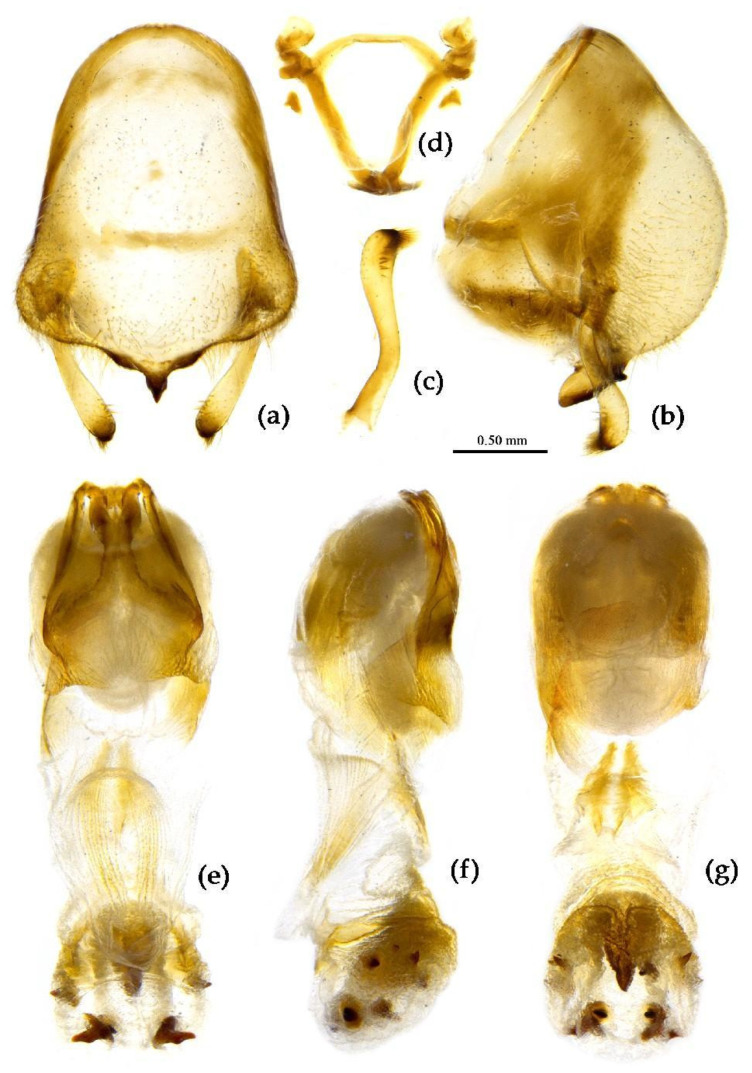
*Sycanus minor* Hsiao, 1979, (**a**–**g**) male external genitalia, (**a**,**b**) pygophore with parameres, (**c**) paramere, (**d**) phallobase, (**e**–**g**) phallosoma, (**e**) dorsal view, (**b**,**f**) lateral view, (**a**,**g**) ventral view.

**(10) *Sycanus minor* Hsiao, 1979**
(Figure 2h, Figure 3h, Figure 4h, Figure 22, Figure 23 and Figures S12e)
*Sycanus minor* Hsiao 1979: 43, 154 [9]; Hsiao & Ren 1981: 521 [10]; Maldonado-Capriles 1990: 313 [3]; Putshkov & Putshkov 1996: 259 [11].
**Chinese common name**: 小犀猎蝽

**Redescription. Coloration.** Body coloration variable greatly, generally dark brown to black, with red markings, sometimes almost totally black (Figure 2h, Figure 22 and Figure S12e). Head (except apical part of rostrum and spot around ocellus yellowish brown), antennae, pronotum (except lateral and posterior margins of posterior lobe orange, or anterior, lateral and posterior margins of pronotum orange), thorax (except coxal cavity orange, or most of pro-pleuron, markings of meso- and meta-pleura, anterior margin of meso- and meta- sterna orange), scutellum (except apical spine orange, posterior margin greyish brown), legs (except coxae orange, sometimes annular markings on sub-apical part of femur and tibiae paler), corium of fore wing (except sub-apical part yellowish or milk white) black; membrane of fore wing semitransparent, blackish brown; abdomen yellowish to red, median longitudinal stripe, transversal stripe of sternum and markings of each connexival segment black (Figure 22). Sometimes body almost entirely black, except posterior margin or transversal tripe of corium of fore wing, sub-apical annular markings of tibiae and femora, subapical annular markings of first segment milk-white, third and fourth antennal segments pale brown. 

**Structure.** Median-sized. Body clothed with white setae and procumbent curved short setae, legs with erect setae of different length. Head longer, postocular part of head longer than anteocular; antennae slender and thin, first segment longest and as long as fourth, third shortest and distinctly shorter than second; rostrum long and bent, first extending to middle part of eyes, second segment longest. Collar process round and indistinct; anterior pronotal lobe small and hemispherical, medially with short longitudinal sulcus at base; posterior pronotal lobe rough, lateral angle round; posterior margin of scutellum round, apical spine erect, its apex bifid (Figure 3h and Figure 22); legs slender; fore wing extending beyond tip of abdomen; fourth to sixth connexival segments dilated laterally (Figure 4h and Figure 22). Pygophore elliptic, median pygophore process “T” shaped (Figure 23a,b); paramere clavate, apical part with setae, middle part somewhat bent (Figure 23c). Phallobase triangular (Figure 23d); phallosoma elliptic, dorsal phallothecal sclerite sclerotized; endosoma medially with two feebly sclerotized stripe-shaped sclerites, apically with a feebly sclerotized horned process and laterally with five pairs of small spines (Figure 23e–g).

**Measurement [♂(n = 3)/♀(n = 1), in mm].** Body length 17.20–18.18/19.27, maximal width of abdomen 8.00–8.80/8.18; Head length 4.46–4.73/4.73; length of anteocular part 1.47–1.64/1.64; length of postocular part 2.15–2.18/2.18; length of synthlipsis 0.43–0.56/0.58; distance between ocelli 0.36–0.55/0.36; length of antennal segments I–IV = 5.20–6.00/5.45, 2.00–2.36/2.00, 0.91–1.10/1.09, 6.00-6.18/5.55; length of visible rostral segments I–III = 1.92–2.18/2.36, 3.15–3.64/3.64, 0.55–0.58/0.55; length of anterior pronotal lobe 0.84–1.09/1.27; length of posterior pronotal lobe 1.71–2.00/2.18; maximal width of pronotum 3.31–3.73/4.36; length of scutellum 1.45/1.64; length of fore wing 9.87–11.82/12.36. 

**Type material. Holotype**, ♂, CHINA, Yunnan, Xishuangbanna, Banna Menglong, Manbing, 1958-IV-14, Cheng Hanhua leg., 650 m, Hsiao Tsai-Yu Det, 1972 (TJNHM). 

**Specimens examined.** CHINA, Guangxi: 2♂, Ningming, Huashan, 2019-VIII-26, Zhao Ping leg. (NNU). 

CHINA, Yunnan: 1♂, Xishuangbanna, Manbing, 1958-IV-14, Zheng leg., Ren Shuzhi Det., 1972 (NKU); 1♂, 1♀, Mengla, Menglu, Cui Jianxin leg. (CAU); 1♂, 1♀, Banna, Jinghong, N21.59955, E100.47813, 530 m, 2009-V-3, Cao Liangming leg. (CAU); 5♂, Banna, Jinghong, Jinuo, Jinuo, 1099 m, 2021-VII-22, Chen Zhaoyang & Liu Qinpen leg. (CAU); 1♀, Xishuangbanna, Menglun botanical garden, Wild Elephant Valley (CAU); 1♂, 1♀, Banna, Jinghong, Jinuo mountain, National Highway 213, 1053 m, 2021-VII-25 (CAU).

**Distribution.** China (Yunnan <Xishuangbanna>, Guangxi <Ningming>); Vietnam.

**Remark.** We found a male specimen deposited in Nankai University, China, with the same collecting information as the type specimen and identified by Prof. Ren Shuzhi but without a type label, so we could not be sure it is the type specimen. In addition, the body color of *S. minor* is changeable from orange or red with black markings to almost totally black. In the three specimens distributed from Mengla, Jinghong, Xishuangbanna, and Yunnan, China, the body is almost entirely black (except the posterior margin of the corium of the fore wing, which is yellow to milk-white, and the annular markings of the sub-apical part of the femur, which is milk-white). Through the molecular analysis based on DNA barcoding, the result showed that the three specimens should be identified as *S. minor*.

**Figure 24:**
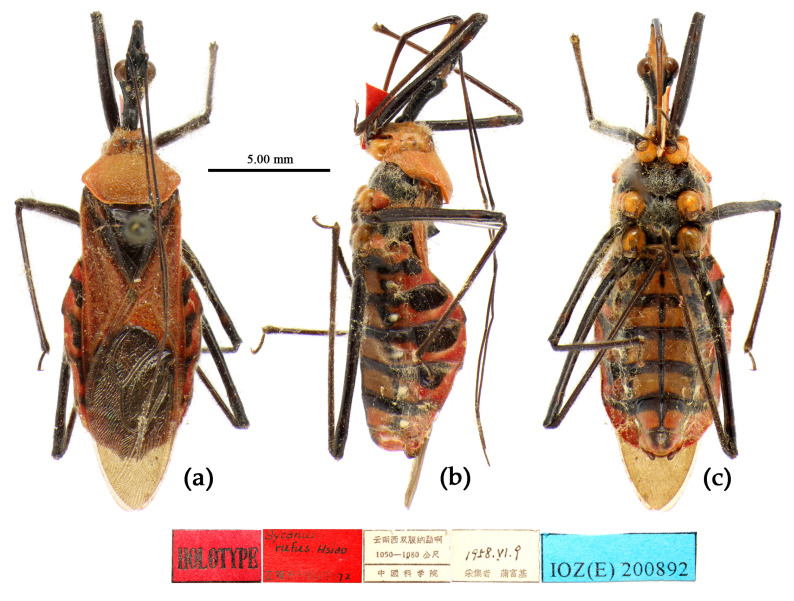
*Sycanus rufus* Hsiao, 1979, **Holotype**, habitus, male (IOZ). (**a**) dorsal view; (**b**) lateral view; (**c**) ventral view.

**Figure 25:**
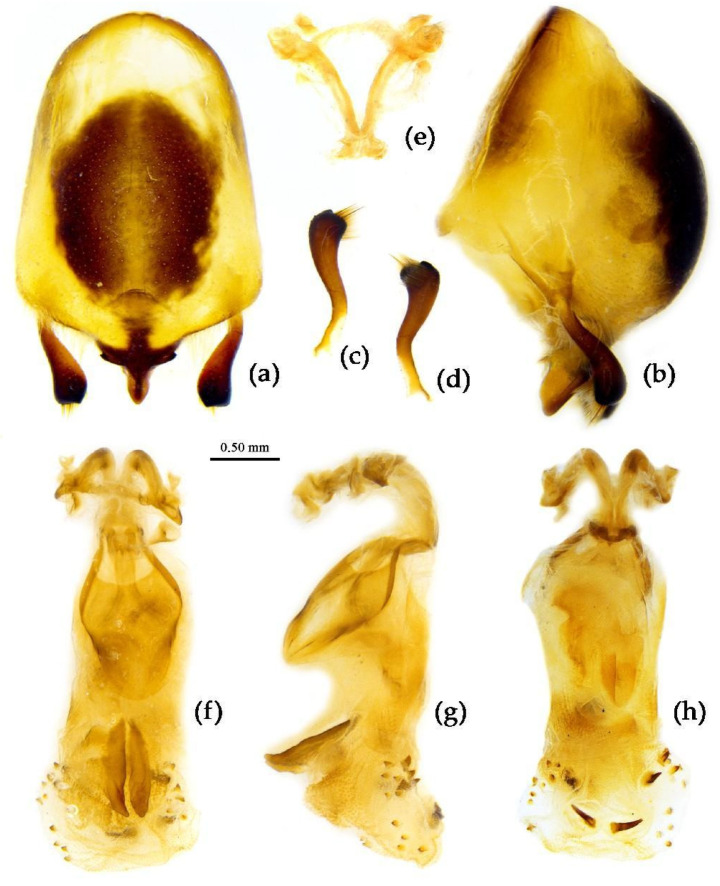
*Sycanus rufus* Hsiao, 1979, (**a**–**h**) male external genitalia, (**a**,**b**) pygophore with parameres, (**c**,**d**) paramere, (**e**) phallobase, (**f**–**h**) phallus, (**f**) dorsal view, (**b**,**g**) lateral view, (**a**,**h**) ventral view.

**(11) *Sycanus rufus* Hsiao, 1979**
(Figure 2i, Figure 3i, Figure 4i, Figure 24 and Figure 25)
*Sycanus rufus* Hsiao, 1979: 141 [9] (Figure 24); Hsiao & Ren, 1981: 522 [10]; Maldonado-Capriles, 1990: 313 [3]; Putshkov & Putshkov, 1996: 260 [11].
**Chinese common name**: 红犀猎蝽

**Redescription. Coloration.** Body red, with black markings (Figure 2i and Figure 24). Head (except apical part of rostrum and annular spot around ocellus yellowish brown), antennae, sterna and pleura of meso- and meta-thoraxes (except coxal cavities orange), scutellum (sometimes except apical spine orange, posterior margin greyish brown), spots on central part of anterior pronotal lobe, legs (except coxae orange, sometimes sub-apical annular markings of femur and tibiae pale brown), basal part of corium of fore wing dark brown to black; membrane of fore wing semitransparent, yellowish brown; abdomen red to yellowish red, median longitudinal stripe, intersegmental transversal stripe and lateral oblique markings of sterna, and large round markings on each connexival segment black, and transversal stripe of third to seventh sterna laterally extended, markings of sixth and seventh not extending to outer margin of connexivum; ventral surface of head, pronotum (except markings of anterior lobe black), pro-pleuron, corium of fore wing (except basal and apical parts black), coxae, coxal cavity red or orange.

**Structure.** Body large-sized. Ventral surface of head, pronotum, pleura and sterna of thorax, corium of fore wing clothed with procumbent short yellow setae; head (except ventral surface), pronotum and propleuron, scutellum, legs, ventral surface of abdomen with setae of different length. Anteocular part of head distinctly shorter than postocular; rostrum incurved, long and slender. Collar process indistinct and round; anterior pronotal lobe small, hemisphere and bulged, deeply medially depressed at base; posterior pronotal lobe rugose and reticulated, lateral angle obtuse and round, posterior margin nearly straight, posterior angle nearly absent; scutellum sub-apically with an erect pyramidal spine (Figure 3i and Figure 24a,b). Femora nearly of equal thickness, apical part somewhat thickened; fore wing extending beyond tip of abdomen. Abdomen moderately laterally expended, especially fourth to sixth connexival segments (Figure 4i and Figure 24). Pygophore elliptic, median pygophore process "Y"-shaped (Figure 25a,b); paramere clavate, apical part with setae, middle part somewhat bent (Figure 25c,d). Phallobase triangular (Figure 25e); phallosoma elliptic, dorsal phallothecal sclerite sclerotized; endosoma medially with two sclerotized horned processes, and laterally with two pairs of larger spines and about 10 pairs of smaller spines (Figure 25f–h).

**Measurement [♂(n = 4)/♀(n = 3), in mm].** Body length 20.29–20.63/24.85–25.88, maximal width of abdomen 5.81–5.99/8.44–10.15; Head length 4.67–4.96/5.24–5.47; length of anteocular part 1.71/1.94–2.05; length of postocular part 2.28/2.45–2.57; length of synthlipsis 0.68–0.73/0.80–0.86; distance between ocelli 0.46/0.51; length of antennal segmentsI–IV = 5.89–6.38/6.95–7.01, 2.51–2.85/2.85–3.02, 2.17–2.45/2.37–2.57, 5.53/5.87–6.67; length of visible rostral segments I–III = 2.28/2.85, 3.42/3.99, 0.68–0.71/0.74–0.80; length of anterior pronotal lobe 1.03–1.14/1.14–1.20; length of posterior pronotal lobe 2.11–2.17/2.25–3.08; maximal width of pronotum 3.93–3.99/5.47–5.76; length of scutellum 1.14–1.25/1.20–1.25; length of fore wing 12.83–12.85/16.82–17.67. 

**Type material. Holotype**, ♂, CHINA, Yunnan, Xishuangbanna, Menga, 1050–1080 m, 9-VI-1958, Fu Fuji leg. (IOZ) (Figure 24). **Allotype**, ♀, same as holotype, 1958-VI-6 (IOZ). **Paratype**, 2♂, 1♀, CHINA, Yunnan, Lancang, Xishuangbanna (IOZ). 

**Specimens examined.** CHINA, Yunnan: 1♂, Mengla, 650 m, V-29 (CAU); 1♀, Xishuangbanna, Mengla, Yaoqu, 2006-VI-5, Wang Hesheng leg. (CAU); 1♀, Banna, Jinghong, Jinuo, Yunfenggusi, 1070 m, 2021-VII-20, Chen Zhaoyang & Liu Qinpeng leg. (CAU); 3♂, 1♀, Xishuangbanna, Mengla, Bubeng, 2012-VII-10, Zhao & Chen leg. (CAU); 1♀, Banna, Jinghong, Jinuo, 1100 m, 2018-V-31 (CAU); 1♀, Pu’er, Simao, 1436 m, 2018-VI-20 (CAU); 1♀, Xishuangbanna, Jinghong Guanping, 2008-V-27, Huang Xinyong leg. (CAU).

**Distribution.** China (Yunnan <Lancang, Pu’er, Xishuangbanna: Mengla, Meng’a, Xiaomengyang, Mengzha>).

**Figure 26:**
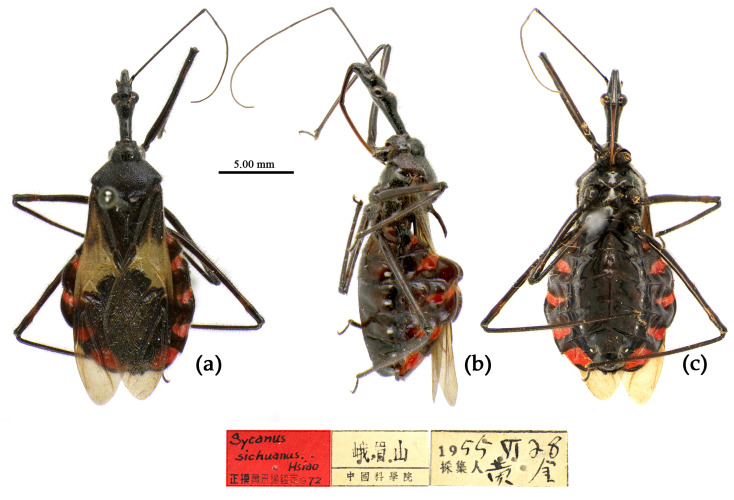
*Sycanus sichuanensis* Hsiao, 1979, **Holotype**, habitus, male (IOZ). (**a**) dorsal view; (**b**) lateral view; (**c**) ventral view.

**Figure 27:**
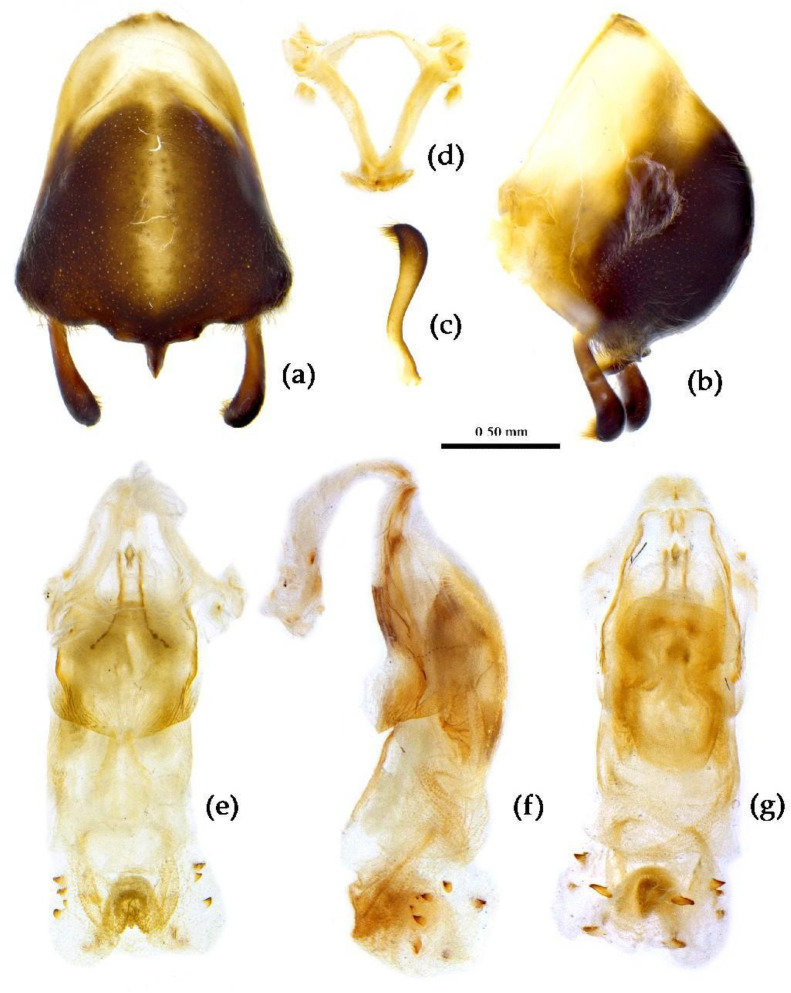
*Sycanus sichuanensis* Hsiao, 1979, (**a**–**g**) male external genitalia; (**a**,**b**) pygophore with parameres; (**c**) paramere; (**d**) phallobase; (**e**–**g**) phallus; (**e**) dorsal view; (**b**,**f**) lateral view; (**a**,**g**) ventral view.

**(12) *Sycanus sichuanensis* Hsiao, 1979**
(Figure 2j, Figure 3j, Figure 4j, Figure 26, Figure 27 and Figures S12f)
*Sycanus sichuanensis* Hsiao, 1979: 153, 154 [9] (Figure 26); Maldonado-Capriles, 1990: 313 [3]; Putshkov & Putshkov, 1996: 260 [11].
*Sycanus szechuanus* (sic): Hsiao & Ren, 1981: 520 [10].
**Chinese common name**: 四川犀猎蝽

**Redescription. Coloration.** Body dark brown to black, slightly shiny, with yellow and red markings (Figure 2j, Figure 26 and Figure S12f). Corium of fore wing (except basal part and apical angle black), basal part of membrane, yellowish to milk-white; third to seventh connexival segments mediately with red transversal markings; membrane semitransparent, brown; femur subapically with an annular brown stripe; annular spot around ocellus yellow; apical part of rostrum brown.

**Structure.** Body median to large sized, oblong and posteriorly widen. Head, thorax, corium of fore wing clothed with procumbent short yellow setae and vertical short setae of different length; legs with vertical setae of different length. Anteocular part of head distinctly shorter than postocular; rostrum incurved, long and slender. Collar process round; anterior pronotal lobe small, hemisphered and bulged, deeply medially depressed at base; posterior pronotal lobe rugose and reticulated, posterolateral angle obtuse and round, posterior margin nearly straight, posterior angle nearly absent; scutellum sub-apically with an long spine, apical spine produced posteriorly, its apex bifid (Figure 3j and Figure 26a,b). Femora nearly of equal thickness, apical part somewhat thickened; fore wing extending beyond tip of abdomen. Abdomen distinctly roundly laterally dilated, and middle part of each connexival segment elevated (Figure 4j and Figure 26). Pygophore elliptic, median pygophore process "T"-shaped shown in Figure 27a,b; paramere clavate, apical half somewhat swelled with thick setae, middle part somewhat bent (Figure 27c). Phallobase triangular (Figure 27d); phallosoma elliptic, dorsal phallothecal sclerite sclerotized; endosoma medially with two very feebly sclerotized stripe-shaped sclerites, apically with a feebly sclerotized horned process and laterally with five pairs of small spines (Figure 27e–g).

**Measurement [♂(n = 8) /♀ (n = 5), in mm].** Body length 16.55–18.73/16.73–22.58; maximal width of abdomen 6.36–7.27/6.36–11.16. head length 4.18–4.73/4.18–5.25; length of anteocular part 1.45–1.55/1.45–1.84; length of postocular part 1.82–2.18/2.00–2.68; distance between ocelli 0.25–0.55/0.29–0.47; length of synthlipsis 0.55–0.73/0.55–2.26; length of antennal segments I–IV = 5.45–6.00/5.82–6.62, 2.00/2.18–2.94, 2.27–2.36/1.64–2.26, 6.50/6.67–6.91; length of visible rostral segments I–III = 1.64–1.82/1.64–2.63, 3.27–3.45/2.73–3.89, 0.64/0.55–0.74; length of anterior pronotal lobe 1.00–1.09/0.91–1.21; length of posterior pronotal lobe 1.82/1.82–2.57; maximal width of pronotum 3.09–4.00/3.36–4.41; length of fore wing 10.18–11.45/10.36–14.70. 

**Type material. Holotype**, ♂, CHINA, Sichuan, Ermeishan, 1955-VI-28, Jin Huang leg. (IOZ) (Figure 26). **Allotype**, ♀, same as Holotype, 1955-VI-23 (IOZ). **Paratypes**: 3♀, 1♂, CHINA, Sichuan, Ermeishan (IOZ); 1♀, 1♂, CHINA, Sichuan, Ermeishan, 580 m, 1955-VI-27, Xie Dabin leg. (IOZ); 1♀, 3♂, CHINA, Sichuan, Er’meishan, Huang Keren & Jin Yintao leg. (IOZ).

**Specimens examined.** CHINA, Guangxi: 1♂, Longzhou, Longrui, 1984-V-20, Ren Shuzhi leg. (NKU); 1♂, Tianpingshan, 1963-VI-17 (NKU); 1♀, Nonggang, Longzhou, 2003-VIII-15, Zhou Zhihong leg. (CAU); 1♀, Huaping, Tianping mountain, 1963-VI-5, Yang Jikun leg. (NKU); 1♂, 1♀, Jinxiu, 1983-V-27 (NKU); 1♀, Lonhzhou, Nonggang, 1983-V-15 (NKU); 1♀, Pingxiang, Orchid Valley Park, 2014-X-8, Sun & Luo leg. (CAU); 1♀, Guilin, Huaping, Tianping mountain, 1963-VI-5, Yang Jikun leg. (NKU); 1♀, Longzhou, Nonggang, 2003-VIII-15, Zhou Zhihong leg. (CAU); 1♂, 1♀, Jinxiu, 1983-V-27 (NKU); 1♀, Longzhou, Nonggang, 1983-V-15. (NKU).

CHINA, Sichuan: 1♀, Ermeishan, 600–1200 m, 2019-VI-27 (CAU); 1♀, Ermeishan, 580 m, 19-VI, Huang Keren leg. (NKU); 1 ♀, Chengdu, Pengzhou, Bailu Town, 800 m, 2018-VII-21, Zhou Chao leg. (CAU); 1♀, Leshan, Emeishan, 2014-VII-3, Liang Si leg. (CAU).

CHINA, Guizhou: 1♀, Rongjiang, Pingyang, 693 m, 2016-VI-20–24, Wu Shengsheng leg. (CAU); 1♀, Rongjiang, Pingyang, Xiaodanjiang, 920–970 m, 2005-VI-3, Zhao Ping leg. (NNU).

CHINA, Yunnan: 1♀, Honghe, Hekou, Huayu cave, 334 m, 2016-IV-23, Yang Xiaodong leg. (CAU); 1♂, Honghekou, Huayu cave, 200 m, 2016-IV-24, Yu Tang Wang leg. (CAU).

CHINA, Hunan: 1♀, Yongzhou, Ningyuan, Jiuyi mountain, 915 m, 2021-VI-4–7, Peng Huoliang leg. (CAU).

**Distribution.** China (Hunan <Xiangxi, Xiangnan, Yonhzhou>, Hubei, Sichuan <Chengdu, Ermeishan, Ya’nan, Xingjing>, Guizhou<Maolan, Rongjiang, Shiqian>, Guangxi<Longsheng, Longzhou, Guilin, Pingxiang, Jinxiu, Huaping>, Yunan <Gengma, Honghe>); Vietnam.

**Figure 28:**
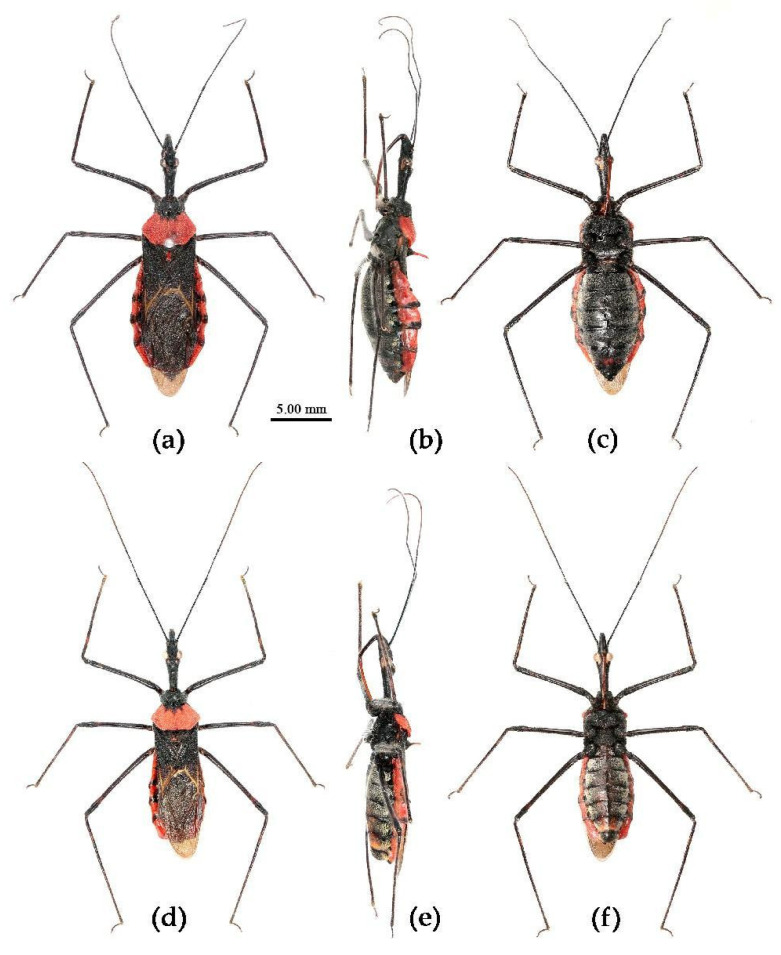
*Sycanus taiwanensis* Zhao & Cai **sp. nov.**, habitus, (**a**–**c**) **Holotype**, female (CAU), (**d**–**f**) **Paratype,** male (CAU), (**a**,**d**) dorsal view, (**b**,**e**) lateral view, (**c**,**f**) ventral view.

**Figure 29:**
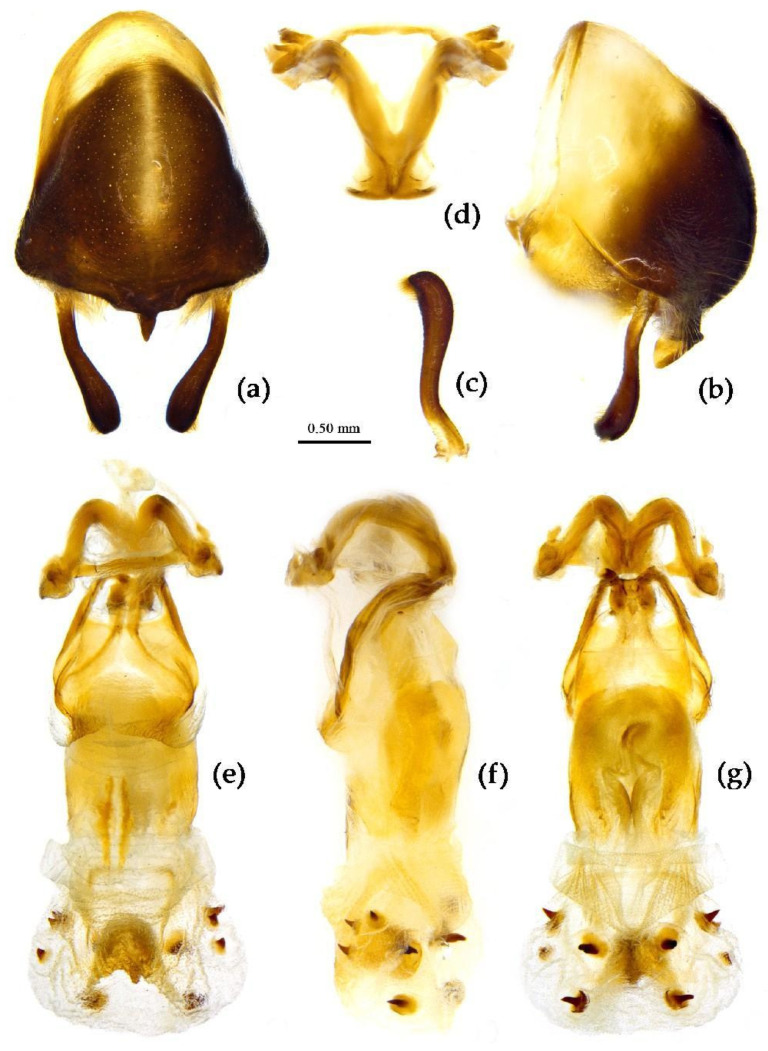
*Sycanus taiwanensis* Zhao & Cai sp. nov., (**a**–**g**) **Paratype**, male external genitalia, (**a**,**b**) pygophore with parameres, (**c**) paramere; (**d**, phallobase; (**e**–**g**) phallus; (**e**) dorsal view; (**b**,**f**) lateral view; (**a**,**g**) ventral view.

**(13) *Sycanus taiwanensis* Zhao & Cai sp. nov.**
urn:lsid:zoobank.org:act:7C6888E2-C8F2-447A-AFFE-F2AC9D3662C5
(Figure 2k, Figure 3k, Figure 4k, Figure 28 and Figure 29)
**Chinese common name**: 台湾犀猎蝽

**Diagnosis.** The male external genitalia of the new species are the most similar to that of *S. sichuanensis*, but there are differences in external body color and structure. In the new species, the body is thin and long, the connexivum is laterally dilated, the posterior pronotal lobe is red (except the middle part, which is black) and posterior margin of corium of fore wing is milk-white (vs. in *S. sichuanensis*, where the body is posteriorly widened, the connexivum is wide and round, the middle part of each segment of connexivum is elevated, the pronotum is totally black, and most of corium of fore wing is white) (Figure 2k and Figure 28).

**Description. Coloration.** Body black, with red markings (Figure 2k and Figure 28). Head (except rostrum and markings around ocelli yellowish brown), antennae, thorax (except lateral and posterior margins of posterior lobe red or posterior lobe totally red), scutellum (except apical spine red), legs, corium of fore wing (except posterior margin yellowish), abdomen (except connexivum) black; membrane of fore wing semitransparent, brown; connexivum red, markings of fourth to seventh inter-segmental sutures black (Figure 28).

**Structure.** Body large-sized. Head, thorax, ventral surface of abdomen, corium of fore wing clothed with procumbent short white setae; legs with pale vertical setae of different length. Anteocular part distinctly shorter than postocular; rostrum incurved, long and slender; antennae slender, first segment longest and somewhat longer than fourth, third shortest and a little shorter than second. Collar process round; anterior pronotal lobe small, hemispherical and bulged; posterior pronotal lobe rugose and reticulated, lateral angle obtuse and round, posterior margin nearly straight; scutellum sub-apically with an erect long spine, its apex bifid (Figure 3k and Figure 28). Femora nearly of equal thickness, apical part somewhat thickened; fore wing extending beyond tip of abdomen. Fourth and fifth connexival segments of abdomen moderately expended (Figure 4k and Figure 28), posterolateral angles of third to fifth connexival segments laterally roundly produced. Pygophore elliptic, median pygophore process "T"-shaped (Figure 29a,b); paramere clavate, apical half somewhat swelled with thick setae, middle part somewhat bent (Figure 29c). Phallobase triangular (Figure 29d); phallosoma elliptic; dorsal phallothecal sclerite sclerotized; endosoma apically with a feebly sclerotized horned process and laterally with five pairs of small spines, and middle part dorsally with two short and slender sclerites (Figure 29e–g).

**Measurement [**Holotype, **♂(n = 3)/♀(n = 3), in mm].** Body length 20.08, 18.73–20.41/22.8–20.00, maximal width of abdomen 6.09, 5.24–6.73/6.09–6.91; Head length 5.91, 4.55–5.13/5.64–6.91; length of anteocular part 1.91, 1.73–1.77/1.88–2.00; length of postocular part 2.82, 2.00–2.57/2.36–2.82; length of synthlipsis 0.66, 0.69–0.80/0.66–0.80; distance between ocelli 0.30, 0.36–0.57/0.30–0.57; length of antennal segments I–IV = 6.21, 5.45–6.56/6.00–6.44, 2.87, 2.36–2.62/2.62–2.87, 1.57, 1.54/1.68, 5.80, 5.00/5.80; length of visible rostral segments I–III = 2.41, 2.00–2.45/2.18–2.41, 3.97, 3.45–3.99/3.64–3.97, 0.71, 0.55–0.74/0.55–0.86; length of anterior pronotal lobe 1.23, 1.09–1.25/1.09–1.37; length of posterior pronotal lobe 2.64, 1.82–2.22/2.39–2.64; maximal width of pronotum 4.31, 3.82–3.99 /4.36–4.39; length of scutellum 1.09, 0.86/1.09–1.25; length of fore wing 13.74, 11.82–12.56 /12.73–13.97.

**Type material. Holotype**, ♀, CHINA, Taiwan, Pingtung County, Lilong Mountain, 650 m, 2015-I-26 (CAU) (Figure 28a–c). **Paratypes**. 1♂, CHINA, Taiwan, Pingtung County, Lilong mountain, 650 m, 2015-I-26 (CAU) (Figure 28d–f); 1♂, CHINA, Taiwan, Pingtung County, Manchu Township, Jialeshui, 2019-XI-08, Liu Xingyue leg. (ZMUC); 1♀, CHINA, Taiwan, Pingtung County, Manchu Township, Jialeshui, 2010-V-29, Zhou Wenyi leg. (ZMUC); 1♀, CHINA, Taiwan, Pingtung County, Heng-Chun, Ken-Ting, 400 m, 2016-VIII-23, Wu S.P. & Chung Y.T. leg. (MfN); 1♂, CHINA, Taiwan, Pingtung County, Fenggang, Lilong Moutain, 650 m, 2015-I-26, Zhong Yiting leg. (MfN); 1♀, CHINA, Taiwan, Pingtung County, Heng-Chun, Ken-Ting, 2010-X-7, Cai Wanzhi leg. (CAU); 2♂, 4♀, CHINA, Taiwan, Pingtung County, Lilong mountain, 650 m, 2015-VIII-17, Y.-T. Chung leg. (CAU); 1♂, 2♀, CHINA, Taiwan, Pingtung County, Lilong mountain, 651 m, 2015-VIII-18, Chung Y.T. leg. (CAU); 1♀, CHINA, Taiwan, Pingtung County, Manchu Township, Gangkou, 2010-VI-13, Zhou Wenyi leg. (CAU); 1♀, CHINA, Taiwan, Pingtung County, Neipu Township, 2001-VI-23, Wu Shuping leg. (CAU); 1♂, CHINA, Taiwan, Kaohsiung City, Taoyuan, Zhou W.Y. leg. (CAU); 1♀, CHINA, Taiwan, Kaohsiung City, Taoyuan District, Meilong Mountain, 1370 m, 2016-VI-15, Po-Hsin Kuo leg. (CAU); 1♀, CHINA, Taiwan, Kaohsiung, Taoyuan, Xiaoguanshan forest road, 1523 m, 2016-V-15, Po-Hsin Kuo leg. (CAU); 1♀, CHINA, Taiwan, Kaohsiung, Maolin (CAU), 365 m, 2012-X-26, (CAU); 1♀, CHINA, Taiwan, Chiayi County, Alishan Township, Danayi Valley, 2012-VI-23, Song Fan leg. (CAU); 1♀, CHINA, Taiwan, Xinbei City, Sanxia District, Beichatian Mountain, 2016-VII-19, Wu Shuping leg. (CAU); 1♀, CHINA, Taiwan, Nantou country, Endemic Species Research Institute, 2016-XI-4, Zhao Yisheng leg. (CAU).

**Etymology.** The specific name *taiwanensis* alludes to the locality of the type species in Taiwan, China. 

**Distribution.** China (Taiwan).

**Figure 30:**
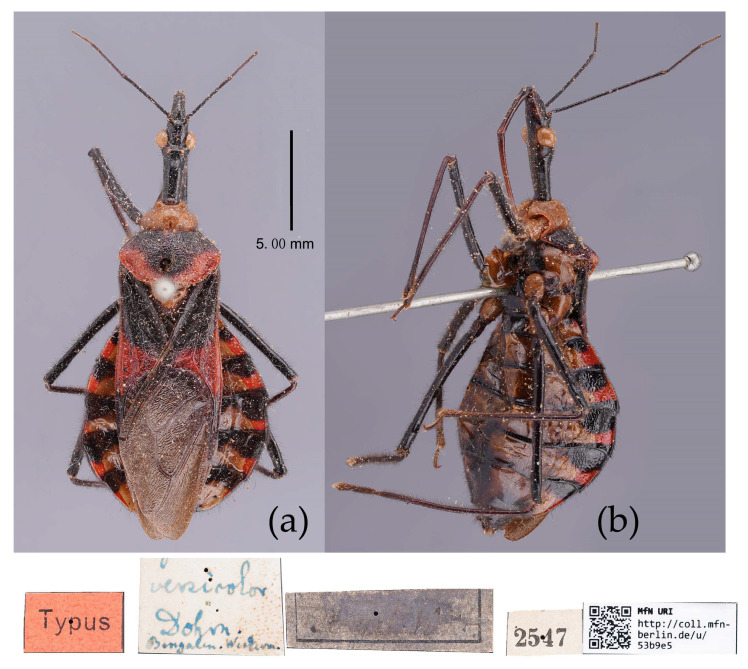
(**a**,**b**), *Sycanus versicolor* Dohrn, 1859, **Type**, female (MfN), habitus. (**a**) dorsal view; (**b**) lateral view.

**Figure 31:**
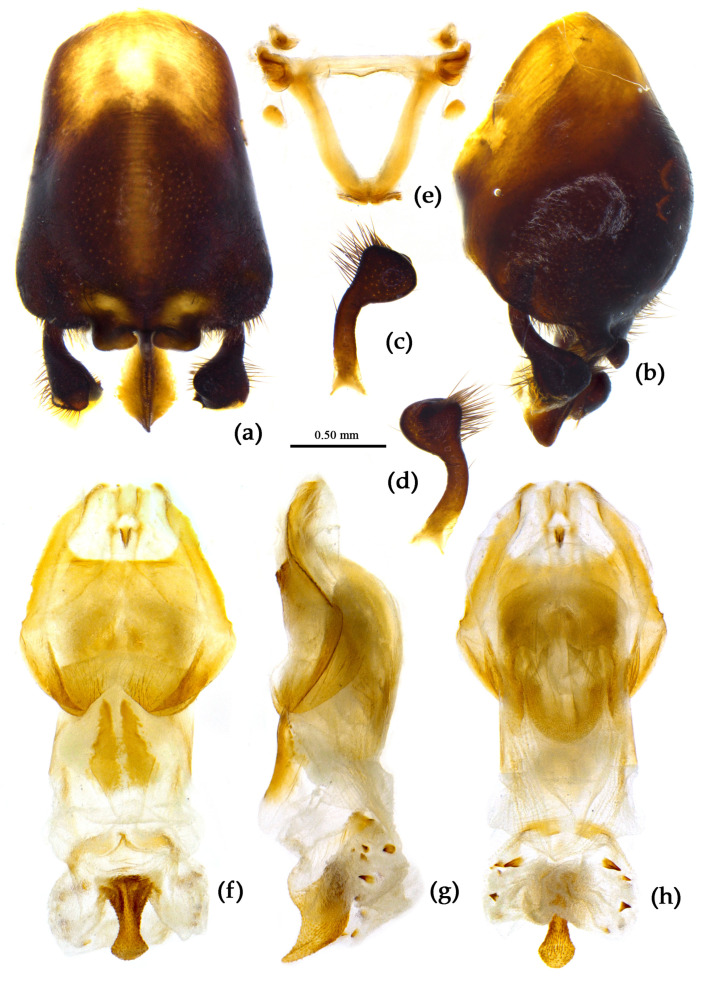
*Sycanus versicolor* Dohrn, 1859, (**a**–**h**) male external genitalia; (**a**,**b**) pygophore with parameres; (**c**,**d**) paramere; (**e**) phallobase; (**f**–**h**) phallosoma; (**f**) dorsal view; (**b**,**g**) lateral view; (**a**,**h**) ventral view.

**(14) *Sycanus versicolor* Dohrn, 1859**
(Figure 2l, Figure 3l, Figure 4l, Figure 30, Figure 31 and Figures S10 and S11)
*Sycanus versicolor* Dohrn, 1859: 96 [6] (Figure 30).
*Sycanus miles* Walker, 1873: 86 [36]. Synonymized by Distant 1903: 212 [40] (Figure S10a,b).
*Sycanus bicolor* Hsiao, 1979: 141, 154 [9]; Hsiao & Ren, 1981: 523 [10]; Maldonado-Capriles, 1990: 310 [3]; Putshkov & Putshkov, 1996: 259 [11] (Figure S10c–e). **syn. nov.**
**Chinese common name**: 二色犀猎蝽

**Redescription. Coloration.** Body black, with red markings (Figure 2l, Figure 30, Figures S10 and S11). Anterior pronotal lobe, propleural episternum, posterior 1/3 of posterior pronotal lobe, scutellum (except posterior margin), apical half of corium of fore wing (except apical angle black), most of meso- and meta-pleura, coxae, posterior half of each connexival segment red; abdomen black with red markings (Figure 30), or abdomen ventrally totally black (Figure S11); rostrum black, second and third segments gradually paler, reddish brown.

**Structure.** Body large-sized (Figure 2l, Figure 30, Figures S10 and S11). Head beneath, pronotum, pleura and sterna of thorax, corium clothed with procumbent short white setae; head (except ventral surface), pronotum and propleuron, scutellum, legs, sterna of abdomen clothed with longer setae; first antennal segment sparsely with oblique short setae, second segment densely with short setae, third and fourth segments with procumbent short setae. Head longer than pronotum, constricted in middle, anterior lobe sub-equal to 1/2 of posterior lobe in length; postocular part of head round, then posteriorly thinned, and longer than anteocular; ocelli separated, distance between ocelli longer than distance between ipsolateral ocellus and eyes. Anterior pronotal lobe hemispherical, smooth, median longitudinal sulcus short and deep; posterior pronotal lobe with irregular wrinkles; lateral pronotal angle round and obtuse; posterior and posterior-lateral margins of pronotum nearly straight; apical spine of scutellum situated at middle part, thick and short, erect (Figure 3l, Figure 30, Figures S10 and S11). Fore wing extending beyond tip of abdomen. Abdomen laterally roundly dilated (Figure 4l, Figure 30, Figures S10 and S11). Pygophore elliptic, median pygophore process with two ear-shaped processes and a median large posteriorly produced process (Figure 31a,b); paramere clavate, apical half swelled with thick setae, middle part bent (Figure 31c,d). Phallobase triangular (Figure 31e); phallosoma elliptic; dorsal phallothecal sclerite widened laterally; endosoma medially with two weakly sclerotized stripe-shaped sclerites, apically with a well sclerotized horned process and laterally with five pairs of small spines (Figure 31f–h).

**Measurement [♂(n = 4)/♀(n = 3), in mm].** Body length 17.21–18.47/20.75–23.09, maximal width of abdomen 5.70–7.41/7.41–7.70; Head length 4.56–4.85/5.13–5.42; length of anteocular part 1.60–1.80/1.82–2.00; length of postocular part 1.82–2.11/2.34–2.39; length of synthlipsis 0.68–0.80/0.86; distance between ocelli 0.40–0.46/0.46; length of antennal segments I–IV = 4.62–5.70/4.85–5.24, 2.28–2.30/2.00, 1.03–1.10/0.97, 7.41–7.50/3.99–5.13; length of visible rostral segments I–III = 2.00–2.39/2.39–2.51, 3.14–3.71/3.99–5.42, 0.63–0.68/0.71–0.80; length of anterior pronotal lobe 1.14–1.25/1.31–1.37; length of posterior pronotal lobe 1.73–2.00/2.57–2.68; maximal width of pronotum 3.53–3.93/4.85; length of scutellum 0.68–1.03/1.43–1.48; length of fore wing 10.83–11.40/13.00–13.68.

**Type material. Holotype** of *Sycanus versicolor* Dohrn, 1859, ♀, collected from Bengalen, 2547 (MfN) (Figure 30).

**Holotype** of *Sycanus miles* Walker, 1873, ♂, collected from Penang, BMNH(E)1255109, (BMNH) (Figure S10a,b).

**Holotype** of *Sycanus bicolor* Hsiao, 1979, ♂, CHINA, Yunnan, Xishuangbanna, Mengzhe, 1400 m, 1958-VI-15, Zhu Zhibin leg. (IOZ). **Allotype**, ♀, same as holotype, 15-VI-1958, Cheng Hanhua leg. (TJNHM). **Paratypes**, 2♂, 3♀, CHINA, Yunnan, Xishuangbanna, Xiaomengyang, Mengzhe, Damenglong, Luxi and river valleys in west area of Nujiang River (IOZ); 1♀, CHINA, Yunnan, Ruili, 1400 m, 1956-VI-6, Zhou Bentao leg. (IOZ) (Figure 10c–e).

**Specimens examined.** CHINA, Yunnan: 2♂, Yuanjiang, 1976-VII-20, Xiong Shaoda leg. (CAU); 1♀, Yongdeng, Hongcheng, 1980-X-4, Xi Gengsi leg. (CAU); 3♂, 3♀, Mengla, Yaoqu, 2005-V-9, 850 m, 21°43′ N, 101°32′ E, Bai Xiaoshuan & Cui Jianxin leg. (CAU); 1♀, Mengla, Shangyong, 2005-V-18, Cui Jianxin leg. (CAU); 1♂, 1♀, Mengla, 1979-IX-18, 650 m, Zheng Leyi leg. (NKU); 1♂, Xishuangbanna, Xiaomengyang, 1957-IX-12, Wang Shuyong leg. (IOZ); 2♂, 1♀, Mangshi, 1000 m, 1956-VI-3-4, 1959-VI-29, Huang Tianrong & Zhou Benshou leg. (NKU); 1♂, 1958-VI-16, 1♀, 1958-VI-14, Jingdong, 1170 m (NKU); 1♂, Zhenyuan, 1400 m, VI-23 (IOZ); 1♂, 1♀, Xishuangbanna, Mengzhe, 1200 m, 1958-VI-13, Pu Zhelong leg. (IOZ); 2♂, Xishuangbanna, Mengzhe, 1400 m, 1400 m, 1958-VI-14 (NKU); 1♀, Xishuangbanna, Menglong, 650 m (IOZ); 1♂, West Nujianghegu, 800 m, 1955-V-9 (NKU); 1♂, Longkou, Longjiang, Mengqiao, 1650 m, 1979-VI-15 (NKU); 1♂, Lianghe (NKU). 

**Distribution.** China (Yunnan <Luxi, river valleys in west area of Nujiang River, Yuanjiang, Jingdong, Hongcheng, Mangshi, Mengla, Menglong, Mengzhe, Xiaomengyang, Damenglong, Lianghe, Zhenyuan, Yongdeng, Ruili>); Bengalen, Penang.

**Remark.** After we examined the type specimen’s photograph of *Sycanus miles* Walker, 1873 (Figure S10a,b) [36] (Penang), male, deposited in BMNH (URL: https://data.nhm.ac.uk/object/836db954-c751-4d1c-8f45-bfde377bea1c, accessed on 26 February 2024), we found that *Sycanus bicolor* Hsiao, 1979 [9] (Figure S10c–e), was similar to *S. miles* in body coloration and structure. However, Distant [40] had assigned *S. miles* as a synonym of *Sycanus versicolor* Dohrn, 1859 (India) [3,6]. After we examined the type specimens of *S. versicolor* in MfN [6], we thought that *S. miles* (Figure S10a,b) and *S. bicolor* (Figure S10c–e) are two synonyms of *S. versicolor* (Figure 30).

## 4. Discussion

### 4.1. Species Delimitation of Chinese Sycanus

In the integrative taxonomic study of the assassin bug genus *Sycanus* in China, we conducted species delimitation and phylogenetic analyses using a dataset of 81 COI DNA barcodes of 12 species identified based on morphological characters. All samples included in this study were identified as belonging to 11 MOTUs (Figure 1 and Figures S1 and S2) because *S. versicolor* and *S. bifidus* failed to be divided (Figure 1 and Figures S1 and S2).

The external morphological differences between *S. versicolor* (Figure 30 and Figure 31) and *S. bifidus* (Figure 7 and Figure 8) are significant. In *S. versicolor*, the body is red with black markings (vs. in *S. bifidus*, where the body is black and the middle part of the corium has a yellow transversal stripe). However, the structure of the male genitalia of *S. versicolor* is similar to that of *S. bifidus* as mentioned above. We speculated that external morphological characters evolve faster under selective pressures than genetic ones [41]. Therefore, it is possible that these two species separated from each other recently, and the complete lineage sorting has not yet been achieved.

### 4.2. Discovery of Island Endemic and Cryptic Species

The prolonged geographic separation between islands and the mainland has resulted in the isolation of the biota found on these islands. In response to the challenges posed by the islands’ environment, radiation adaptations swiftly occur, ultimately leading to speciation events [15,17,42,43,44]. As a result, the continental islands, as natural laboratories of evolution, are distributed with many island endemic species that have become isolated from their mainland counterparts [42,43,44]. In China, Taiwan and Hainan are two large continental islands that form endemic biotas [42,43,44,45].

We discovered in this study two interesting speciation cases within the *Sycanus* genus in the Taiwan and the Hainan islands of China, which were achieved through species delimitation and a phylogeny analysis using COI DNA barcoding. In one case, the molecular analysis results showed that the *S. sichuanensis* species group consists of four species, including the two previously identified continental species *S. sichuanensis* and *S. minor* as well as *S. taiwanensis* sp. nov. in Taiwan island and *S. hainanensis* sp. nov. in Hainan Island, which are two new island endemic cryptic species (Figure 1a; Tables S2 and S3). In the other case, the *S. croceus* species group includes two mainland species, *S. croceus* and *S. rufus,* along with one island endemic known species *S. insularis*, which exhibits morphological similarities to *S. croceus* in terms of body shape and coloration. The results of the species delimitation analysis, conducted using the COI DNA barcode dataset, revealed a genetic distance of 4.28% (>2%) between *S. insularis* and *S. croceus* and classified *S. insularis* as an independent island endemic species due to the prolonged isolation on the island (Figure 1b; Tables S2 and S3). 

The body shape and coloration of the new cryptic species *S*. *flavicorius* Li & Cai sp. nov. from Yunan province, China, were similar to those of *S. croceus* Hsiao, 1979. However, the male genitalia structure of *S. flavicorius* sp. nov. resembled those of *S. marginellus* and *S. falleni*, and the three species shared the same clade with the *S. sichuanensis* species group, and the *S. croceus* species group in the BI tree of the molecular phylogenetic analysis, but the relationship is uncertain (Figure 1c, Tables S2 and S3). Since our taxonomic study on the genus *Sycanus* was limited to the Chinese species and did not include global species, further studies are needed to better explore the interspecific phylogenetic relationship of the genus *Sycanus*.

### 4.3. Polymorphism

Some of the *Sycanus* species show polymorphism in terms of body coloration and size, making it challenging to identify these species accurately. Through a molecular analysis based on COI DNA barcodes, the morphological taxonomic results have been objectively rectified and corrected. 

In *S. falleni*, when we raised them (Figure 11 and Figures S6 and S12c,d), we found that there are two body coloration (bright red and dark red) in adults, which hatched from the one egg mass laid by one female adult. 

In *S. bifidus* (Figure 7 and Figures S3, S4 and S12a,b), the corium of the fore wing displays a transverse stripe of either yellow white or milk-white, reddish brwon. We obtained DNA barcodes of 27 samples of *S. bifidus* (18 samples from Guangxi, 3 from Guangdong, 6 from Fujian, China). No genetic divergences in 27 samples of *S. bifidus* were found in the phylogenetic analyses and species delimitation.

In *S. sichuanensis*, there is variation in body size and the transverse stripe on the corium of fore wing, with the body length ranging from 16.55 to 18.73 mm in males and from 16.73 to 22.58 mm in females, and the apical half or margin of the corium is white (Figure 2j, Figure 26 and Figure S12f). The smaller individual of *S. sichuanensis* distributed in Yunnan, China (Table A1, Sample Number: SCX-1-COI) and southwest Guangxi, China (Table A1, Sample Number: SCX-11-COI, SCX-GX1-COI, SCX-GX3-COI), bears resemblance to *S. minor* in terms of body coloration, size, and shape. In *S. minor*, body coloration can range from orange with black markings (Figure 2h, Figure 22 and Figure S12e) to completely black (Table A1, Sample Number: XX-HC-YN2-COI, XX-HC-YN3-COI). Therefore, we could not distinguish between the two species during the experimental research process. However, now, we clearly distinguish two species according to the results of the phylogenetic tree and species delimitation (Figure 1a; Tables S2 and S3).

## 5. Conclusions

In the present study, fourteen species from the genus *Sycanus* in China were described, redescribed, and illustrated, including three new species. The results from the analyses of the combined COI DNA barcoding sequences and the morphological data, discovered two Chinese continental island cryptic species, *S. taiwanensis* Zhao & Cai sp. nov. and *S. hainanensis* Wang & Cai sp. nov. from Taiwan and Hainan, and a cryptic species, *S. flavicorius* Li & Cai sp. nov. from Southwest China. Moreover, we proposed five synonyms for three *Sycanus* species; three synonyms of *S. bifidus* (Fabricius, 1787): *S. croceovittatus* Dohrn, 1859, *S. leucomesus* Walker, 1873, *S. villicus* Stål, 1863, the synonym of *S. versicolor* Dohrn, 1859: *S. bicolor* Hsiao, 1979; and one synonym of *S. marginellus* Putshkov, 1987: *S. hsiaoi* Maldonado-Capriles, 1990. The taxonomic statuses of the species with distinct individual variations in body coloration and size, such as *S. falleni*, *S. bifidus, S. sichuanensis*, and *S. minor*, could be confirmed by the results of species delimitation and phylogenetic analyses. However, we also found the limitation of DNA barcoding, such as the failure of species delimitation between *S. versicolor* and *S. bifidus*. Further studies combining external morphology and internal anatomy as well as more diverse molecular markers are needed to better explore the relationships among species in the genus *Sycanus*.

## Data Availability

All sequences were deposited in the GenBank under accession numbers of OP927069–OP927154 (part sequences of mtDNA COI).

## Supplementary Materials

The following supporting information can be downloaded at: https://www.mdpi.com/article/10.3390/insects15030165/s1, Figure S1: The maximum likelihood (ML) tree of cytochrome *c* oxidase subunit I (COI) sequences for 81 terminals of *Sycanus*, Figure S2: The neighbor-joining (NJ) tree of cytochrome *c* oxidase subunit I (COI) sequences for 81 terminals of *Sycanus*, Figure S3: (a,b) *Sycanus croceovittatus* Dohrn, 1859, Syntype, female, habitus, deposited in MfN; (c,d), *Sycanus villicus* Stål, 1863, Holotype, female, habitus, deposited in BMNH; (e,f), *Sycanus leucomesus* Walker, 1873, Holotype, female, habitus, deposited in BMNH, Figure S4: *Sycanus bifidus* (Fabricius, 1787), habitus, (a–c), male, (d–f), female, from Guangxi, China, deposited in CAU, Figure S5: *Sycanus croceus* Hsiao, 1979, habitus, (a–c), male, (d–f), female, from Guangxi, China, deposited in CAU, Figure S6: *Sycanus falleni* Stål, 1863, habitus, (a–c), female; (d–f), male, from Guangxi, China, deposited in CAU, Figure S7: (a,b), *Sycanus ventralis* Distant, 1919, Holotype, male, habitus, deposited in BMNH, (c,d), *Sycanus viduus* Distant, 1919, Holotype, male, habitus, deposited in BMNH, Figure S8: *Sycanus marginellus* Putshkov, 1987*,* habitus, (a–c), female, (d–f), male, Figure S9: (a,b), *Sycanus stali* Dohrn, 1859, Holotype, female, habitus, deposited in MfN; (c,d), *Sycanus stali* Dohrn, 1859, Paratype, male, habitus, deposited in MfN; (e,f), *S. generosus* Stål, 1863, Syntype, male, habitus, deposited in NRM, Figure S10: (a,b), *Sycanus miles* Walker, 1873, Holotype, deposited in BMNH, female, habitus; (c–e), *Sycanus bicolor* Hsiao, 1979, Paratype, deposited in IOZ, female, habitus, Figure S11: *Sycanus versicolor* Dohrn, 1859, habitus, (a–c), male, (d–f), female, from Yunnan, China, Figure S12: (a, b) *Sycanus bifidus* (Fabricius, 1787), from Guangxi, China; (c,b) *Sycanus falleni* Stål, 1863, Yunnan, China; (e) *Sycanus minor* Hsiao, 1979, from Guangxi, China; (f) *Sycanus sichuanensis* Hsiao, 1979, from Guizhou, China. Table S1: GenBank Accession number under every sample individual code, Table S2: Pairwise genetic divergence (Kimura two-parameter) within and between 12 sampling species of the genus *Sycanus* using cytochrome *c* oxidase subunit I gene sequence (Table S2 is uploaded separately), Table S3: Pairwise genetic divergence (Kimura two-parameter) between 81 individuals of 12 species of the genus *Sycanus* using cytochrome *c* oxidase subunit I gene sequence (Table S3 is uploaded separately); Table S4: Life history of *Sycanus croceus* Hsiao, 1979 (China, Guangxi, Ningming); Table S5: Life history of *Sycanus falleni* Stål, 1863 (China, Guangxi, Ningming); Alignment S1: Alignment of COI DNA barcodes + outgroup dataset (Alignment S1 is uploaded separately).

## Appendix A

**Table A1:** Information on the samples used in the molecular study.

Species	MtDNA COI Barcodes (675 bp)		Locality, Sampling Time, Elevation	Geographical Coordinates
	Sample Code	Sample Size		
Ingroup				
*Sycanus versicolor* Dohrn, 1859	ESX-1–2-COI ESX-1-COI	2	Yaoqu, Mengla, Yunnan, China, 2005-V-7	21°43′22″ N 101° 32′41″ E
	ES-YN1-COI ES-YN2-COI ES-YN3-COI	3	Dengmacaidi, Hehua, Tengchong, Baoshan, Yunnan, China, 2006-VI-2, 1328 m	24°58′21″ N 98°23′48″ E
*Sycanus bifidus* (Fabricius, 1787)	HDX-COI HDX-1-COI HDX-2-COI HDX-3-COI HDX-5-COI HDX-6-COI HDX-8-COI HDX-10-HD2-COI HDX-11-HD3-COI HDX-12-HD4-COI HDX-13-HD5-COI HDX-14-HD6-COI HDX-15-HD7-COI HDX-16-HD8-COI HDX-17-HD-COI	15	Hudiegu, Huashan, Ningming, Guangxi, China, 2014-VIII-12, 127 m	22°12′50″ N 107°3′21″ E
	HDX-12-COI HDX-13-COI HDX-16-COI	3	Nonggang, Longzhou, Guangxi, China, 2014-VIII-15	22°28′24″ N 106°57′11″ E
	HDX-GD1-COI	1	Mashui, Yangchun, Yangjiang, Guangdong, China, 2002-IV-30, 44 m	22°7′29″ N 111°41′23″ E
	HDX-GD2-COI	1	Huizhou, Guangdong, China, 2004-VIII-19, 22 m	23°6′44″ N 114°24′56″ E
	HDX-GD3-COI	1	Meixian, Guangdong, China, 1981-IX-6, 79 m	24°15′58″ N 116°4′53″ E
	HDX-LC1–6-COI	6	Wanmu tea Garden, Nanping, Simao, Puer, Yunnan, China, 2022-VI-15	22°44′50″ N 100°58′32″ E
*Sycanus croceus* Hsiao, 1979	HUX-1-COI-F HUX-2-COI-F HUX-3-COI-F HUX-4-COI-F HUX-5-COI-F	5	Hudiegu, Huashan, Ningming, Guangxi, China, 2014-VIII-12, 374 m	22°12′29″ N 107°3′18″ E
	HUX-6-COI-F HUX-7-COI-F	2	Pingyang, Rongjiang, Guizhou, China, 2016-VI-1	26°22′59″ N 108°5′9″ E
	HUX-FJ1-COI HUX-FJ2-COI HUX-FJ3-COI	3	Shangyong, Dehua, Quanzhou, Fujian, China, 2014-VI-25, 761 m	25°43′37″ N 118°9′46″ E
*Sycanus falleni* Stål,1863	DHX-10-COI DHX-11-COI DHX-13-COI	3	Huashan, Ningming, Guangxi, China, 2014-VI-4, 127 m	22°12′50″ N 107°3′21″ E
	DHX-GX1-COI	1	Longrui, Ningming, Guangxi, China, 2006-V-18	22°12′50″ N 107°3′21″ E
	DHX-GX2-COI	1	Nonggang, Guangxi, China, 2020-V-27	22°28′16″ N 106°57′40″ E
*Sycanus flavicorius* Li & Cai sp. nov.	XZ3-YN1-COI	1	Tianba, Qianliu, Lancang, Yunnan, China, 2017-VII-20, 1375 m	22°53′52″N 100°10′25″E
	XZ3-YN2-COI XZ3-YN3-COI	2	Gengma, Lincang, Yunnan, China, 2019-VII, 1118 m	23°32′17″ N 99°23′49″ E
*Sycanus hainanensis* Wang & Cai sp. nov.	XZ2-HaN1-COI	1	Hainan Tropical Botanical Garden, Nada, Danzhou, Hainan, China, 2016-IV-11, 120 m	19°30′42″ N 109°30′13″ E
	XZ2-HaN2-COI	1	Hongxin, Yuanmen, Baisha, Hainan, China, 2008-X-11, 228 m	19°9′27″ N 109°29′10″ E
*Sycanus insularis* Hsiao, 1979	HBX-HaN1-COI	1	Jianfengling, Ledong, Hainan, China, 2013-IV-12	18°44′34″ N 108°50′54″ E
	HBX-HaN2-COI HBX-HaN3-COI	2	Tianchi, Jianfengling, Ledong, Hainan, China, 2015-V-07, 951 m	18°44′34″ N 108°50′54′ ′E
*Sycanus minor* Hsiao,1979	XX-HC-YN2,-COI XX-HC-YN3-COI	2	Jinghong, Banna, Yunnan, China, 2009-V-3, 530 m	21°35′58″ N 100°28′41″ E
	XX-YN1-COI XX-YN3-COI XX-YN4 XX-YN5 XX-YN9 XX-YN10	6	Jiruo, Jinuo, Jinghong, Xishuangbanna, Yunnan, China, 2021-VII-22, 1090 m	22°3′22″ N 101°59′7″ E
*Sycanus rufus* Hsiao, 1979	HOX-YN2-COI HOX-YN3-COI HOX-YN4-COI	3	Yunfenggushanfeng, Jingmeng, Jinuo, Jinghong, Xishuangbanna, Yunnan, China, 2021-VII-20, 1070 m	22°0′33″ N 100°49′12″ E
*Sycanus sichuanensis* Hsiao, 1979	SCX-1-COI	1	Nanxi, Hekou, Yunnan, China, 2014-V-30, 117 m	22°37′32″ N 103°56′53″ E
	SCX-5-COI	1	Fangxiang, Leishan, Guizhou, China, 2014-VII-22, 900 m	26°26′21″ N 108°16′42″ E
	SCX-8-COI	1	Maolan, Guizhou, China, 2007-V-28, 535 m	25°23′45″ N 108°4′45″ E
	SCX-11-COI	1	Daqingshan, Pingxiang, Chongzuo, Guangxi, China, 2014-VIII-8, 1000 m	22°18′23″ N 106°42′21″ E
	SCX-13-2-COI	1	Huashang, Ningming, Guangxi, China, 262 m	22°15′24″ N 107°0′56″ E
	SCX-GX1-COI	1	Lanhuagu park, Pingxiang, Guangxi, China, 2014-X-8, 127 m	22°12′50″ N 107°3′21″ E
	SCX-GZ1-COI	1	Xiaodanjiang, Pingyang, Rongjiang, Guizhou, China, 2016-VI-20-24, 693 m	26°20′14″ N 108°20′41″ E
	SCX-GX3-COI	1	Nonggang, Longzhou, Guangxi, China, 2003-VIII-15	22°30′14″ N 106°59′0″ E
	SCX-SC1	1	Bailu, Pengzhou, Chengdu, Sichuan, China, 2018-VII-21, 800 m	31°9′32″ N 103°51′54″ E
	SCX-SC2	1	Emei Mountain, Leshan, Sichuan, China, 2014-VII-3, 2215 m	29°32′50″ N 103°20′21″ E
*Sycanus marginellus* Putshkov, 1987	ZYX-YN1-COI	1	Fula, Huimin, Lancang, Yunnan, China, 2017-VII-11, 1055 m	22°15′32″ N 100°4′45″ E
	ZYX-LC9-COI	1	Wanmu tea Garden, Nanping, Simao, Puer, Yunnan, China, 2022-VI-15, 1396 m	22°44′50″ N 100°58′32″ E
*Sycanus taiwanensis* Zhao & Cai sp. nov.	XZ1-TW1-COI	1	Danayi valley, Alishan, Jiayi, Taiwan, China, 2012-VI-23, 42 m	23°28′42″ N 120°27′11″ E
	XZ1-TW2-COI	1	Fengganglilong mountain, Pingdong, Taiwan, China, 2015-I-26, 650 m	22°11′33″N 120°41′10″E
	XZ1-TW3-COI	1	Jialeshui, Manzhou, Pingdong, Taiwan, China, 2019-XI-08, 56 m	21°59′20″ N 120°50′46″ E
Outgroup				
*Agriosphodrus dohrni* (Signoret, 1862)	B-LC1-COI B-LC2-COI	2	Kaili, Guizhou, China, 2017-IV-10, 700 m	26°33′58″ N 107°58′53″ E
*Maldonadocoris annulipes* Zhao, Yuan & Cai, 2006	MS-HN-COI	1	Wuzhishan, Hainan, China, 2022-V-10, 666 m	18°53′0″ N 109°39′45″ E
*Yolinus albopustulatus* China, 1940	DQ-3-COI	1	Heishiding, Fengkai, Zhaoqing, Guangdong, China, 2016-VIII-14, 920 m	23°26′9″ N 111°53′4″ E
	DQ-COI	1	Guiyang, Guizhou, China, 2016-IX-8, 1 200 m	26°38′48″ N 106°37′41″ E
*Yolinus annulicornis* Hsiao, 1979	HJQ-LC-COI	1	Puer, Yunnan, China, 2022-VI-15, 1350 m	22°49′30″ N 100°57′57″ E
