# Supplementary material for: Review of the Genus Sycanus Amyot & Serville, 1843 (Heteroptera: Reduviidae: Harpactorinae), from China Based on DNA Barcoding and Morphological Evidence
Source: Insects. 2024 Feb 28;15(3):165. doi: 10.3390/insects15030165 (PMC10971704; doi:10.3390/insects15030165)
Supplement: Supplementary file 1 [file insects-15-00165-s001.zip › Supporting information-20240217.pdf]

Supporting information

Review of the Genus *Sycanus* Amyot & Serville, 1843 (Heteroptera: Reduviidae: Harpactorinae), from China Based on DNA Barcoding and Morphological Evidence

Ping Zhao [1], Suyi Chen [2,3], Yingqi Liu [2,3], Jianyun Wang [4], Zhuo Chen [2,3], Hu Li [2,3] and Wanzhi Cai [2,3,*]

[1] Key Laboratory of Environment Change and Resources Use in Beibu Gulf (Ministry of Education) and Guangxi Key Laboratory of Earth Surface Processes and Intelligent Simulation, Nanning Normal University, Nanning 530001, China; zpyayjl@126.com
[2] Department of Entomology and MOA Key Lab of Pest Monitoring and Green Management, College of Plant Protection, China Agricultural University, Beijing 100193, China; chensuyiii@126.com (S.C.); yingqiliu0720@163.com (Y.L.); insectchen625@126.com (Z.C.); tigerleecau@hotmail.com (H.L.)
[3] Sanya Institute of China Agricultural University, Sanya 572025, China
[4] Environment and Plant Protection Institute, Chinese Academy of Tropical Agricultural Sciences, Haikou 571101, China; wjy-1989@163.com
* Correspondence: caiwz@cau.edu.cn

http://www.zoobank.org/urn:lsid:zoobank.org:pub:6412AE5B-755B-41B6-AC59-4D0AE0E09904

Figure S1. The maximum likelihood (ML) tree of cytochrome c oxidase subunit I (COI) sequences for 81 terminals of *Sycanus*. The numbers above the branches are bootstrap values. The clades / species identified in this study are indicated in different colors.

Figure S2. The neighbor-joining (NJ) tree of cytochrome c oxidase subunit I (COI) sequences for 81 terminals of *Sycanus*. The numbers above the branches are bootstrap values. The clades / species identified in this study are indicated in different colors.

Figure S3. (a, b) *Sycanus croceovittatus* Dohrn, 1859, **Syntype**, female, habitus, deposited in MfN; (c, d), *Sycanus villicus* Stål, 1863, **Holotype**, female, habitus, deposited in BMNH, URL: https://data.nhm.ac.uk/object/9ac6a020-304f-4c2d-9cd5-7c0def47b4aa; (e, f), *Sycanus leucomesus* Walker, 1873, **Holotype**, female, habitus, deposited in BMNH, URL: https://data.nhm.ac.uk/object/456b420e-6818-4b48-88c6-fa861fa18d76; (a, c, e), dorsal view; (b, d, f), lateral view.

Figure S4. *Sycanus bifidus* (Fabricius, 1787), habitus, (a–c), male, (d–f), female, from Guangxi, China, deposited in CAU. (a, d), dorsal view; (b, e), lateral view; (c, f), ventral view.

Figure S5. *Sycanus croceus* Hsiao, 1979, habitus, (a–c), male, (d–f), female, from Guangxi, China, deposited in CAU. (a, d), dorsal view; (b, e), lateral view; (c, f), ventral view.

Figure S6. *Sycanus falleni* Stål, 1863, habitus, (a–c), female; (d–f), male, from Guangxi, China, deposited in CAU. (a, d), dorsal view; (b, e), lateral view; (c, f), ventral view.

Figure S7. (a, b), *Sycanus ventralis* Distant, 1919, **Holotype**, male, habitus, deposited in BMNH, London, URL: https://data.nhm.ac.uk/object/fd802d93-58b1-4c1e-a72c-49e6cae36788; (c, d), *Sycanus viduus* Distant, 1919, **Holotype**, male, habitus, deposited in BMNH, London, URL: https://data.nhm.ac.uk/object/9ac6a020-304f-4c2d-9cd5-7c0def47b4aa. (a, c) dorsal view; (b, d) lateral view.

Figure S8. *Sycanus generosus* Stål, 1863, habitus, (a–c), female, (d–f), male. (a, d), dorsal view; (b, e), lateral view; (c, f), ventral view.

Figure S9. (a, b), *Sycanus stali* Dohrn, 1859, **Holotype**, female, habitus, deposited in MfN; (c, d), *Sycanus stali* Dohrn, 1859, **Paratype**, male, habitus, deposited in MfN; (e, f), *S. generosus* Stål, 1863, **Syntype**, male, habitus, deposited in NRM, URL: http://www2.nrm.se/en/het_nrm/g/sycanus_generosus.html.

Figure S10. (a, b), *Sycanus miles* Walker, 1873, **Holotype**, deposited in BMNH, female, habitus, URL: https://data.nhm.ac.uk/object/836db954-c751-4d1c-8f45-bfde377bea1c; (c–e), *Sycanus bicolor* Hsiao, 1979, **Paratype**, deposited in IOZ, female, habitus; (a, c), dorsal view; (b, d), lateral view; (e), ventral view.

Figure S11. *Sycanus versicolor* Dohrn, 1859, habitus, (a–c), male, (d–f), female, from Yunnan, China. (a, d), dorsal view; (b, e), lateral view; (c, f), ventral view.

Figure S12. (a, b) *Sycanus bifidus* (Fabricius, 1787), from Guangxi, China; (c, b) *Sycanus falleni* Stål, 1863, Yunnan, China; (e) *Sycanus minor* Hsiao, 1979, from Guangxi, China; (f) *Sycanus sichuanensis* Hsiao, 1979, from Guizhou, China.

Table S1. GenBank Accession number under every sample individual code.

Table S2. Pairwise genetic divergence (Kimura two-parameter) within and between 12 sampling species of the genus *Sycanus* using cytochrome *c* oxidase subunit I gene sequence (Table S2 is uploaded separately)

Table S3. Pairwise genetic divergence (Kimura two-parameter) between 81 individuals of 12 species of the genus *Sycanus* using cytochrome *c* oxidase subunit I gene sequence (Table S3 is uploaded separately)

Table S4. Life history of *Sycanus croceus* Hsiao, 1979 (China, Guangxi, Ningming)

Table S5. Life history of *Sycanus falleni* Stål, 1863 (China, Guangxi, Ningming)

Alignment S1. Alignment of COI DNA barcodes + outgroup dataset (Alignment S1 is uploaded separately)

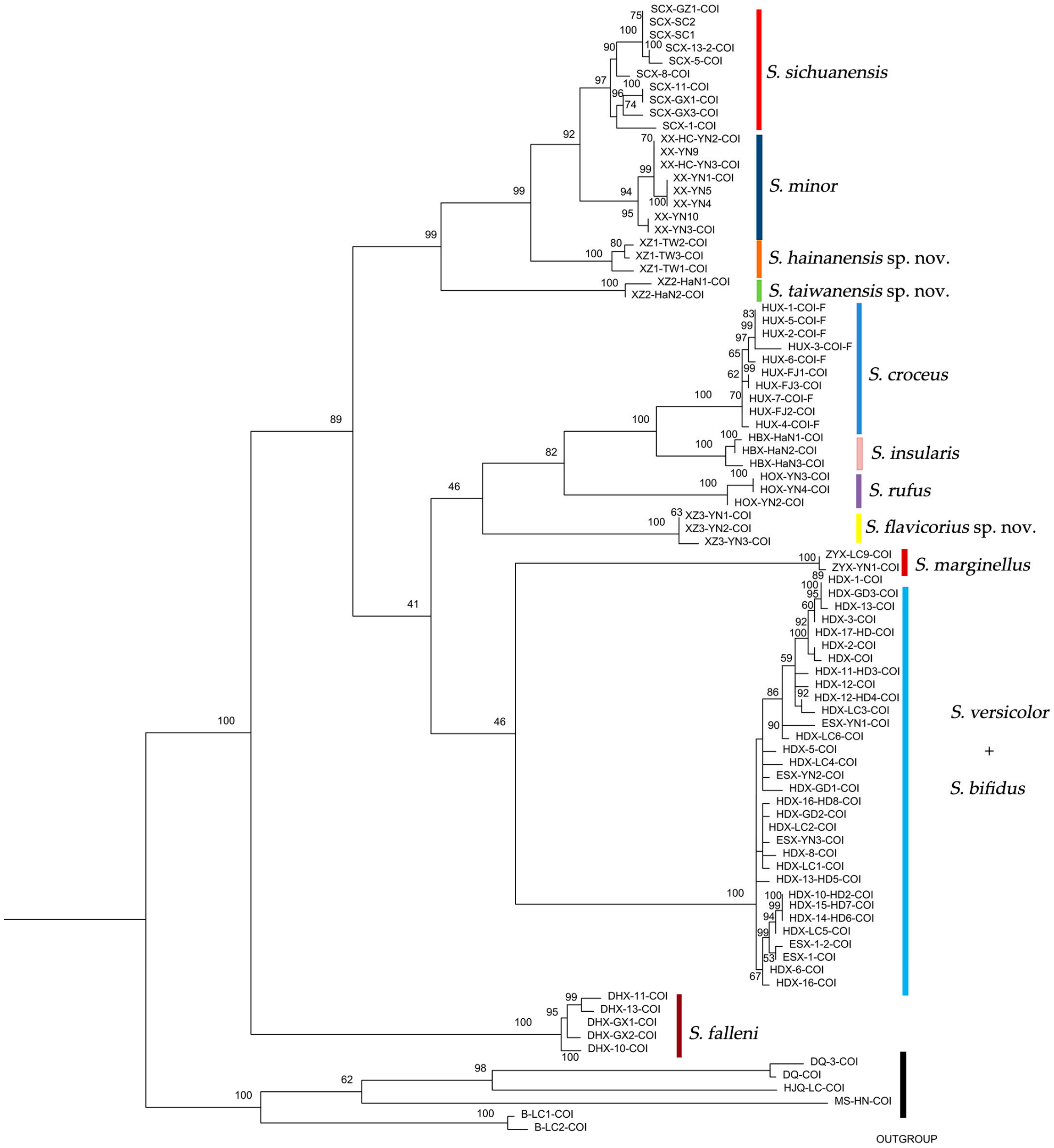

Figure S1. The maximum likelihood (ML) tree of cytochrome c oxidase subunit I (COI) sequences for 81 terminals of *Sycanus*. The numbers above the branches are bootstrap values. The clades / species identified in this study are indicated in different colors.

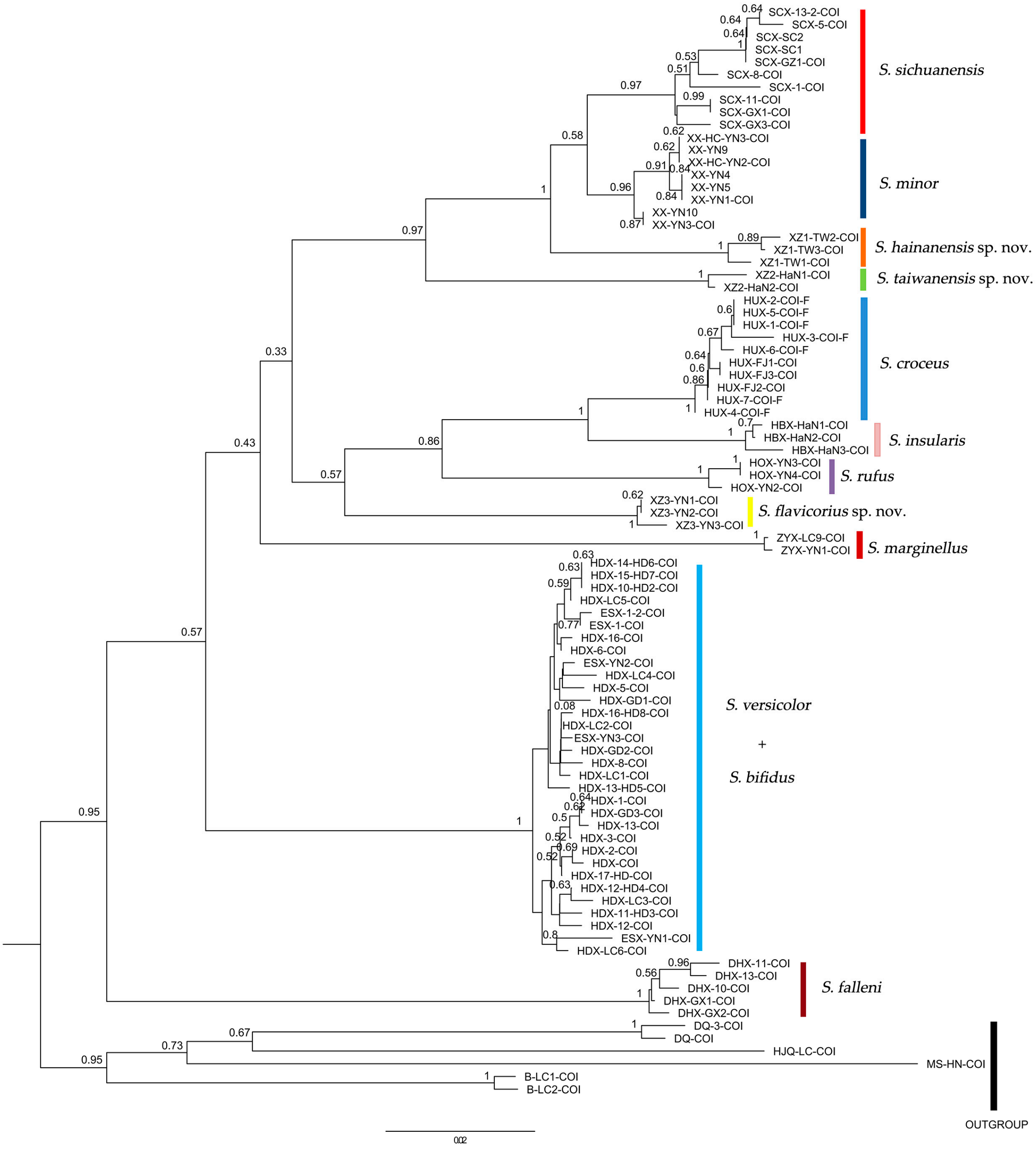

Figure S2. The neighbor-joining (NJ) tree of cytochrome c oxidase subunit I (COI) sequences for 81 terminals of *Sycanus*. The numbers above the branches are bootstrap values. The clades / species identified in this study are indicated in different colors.

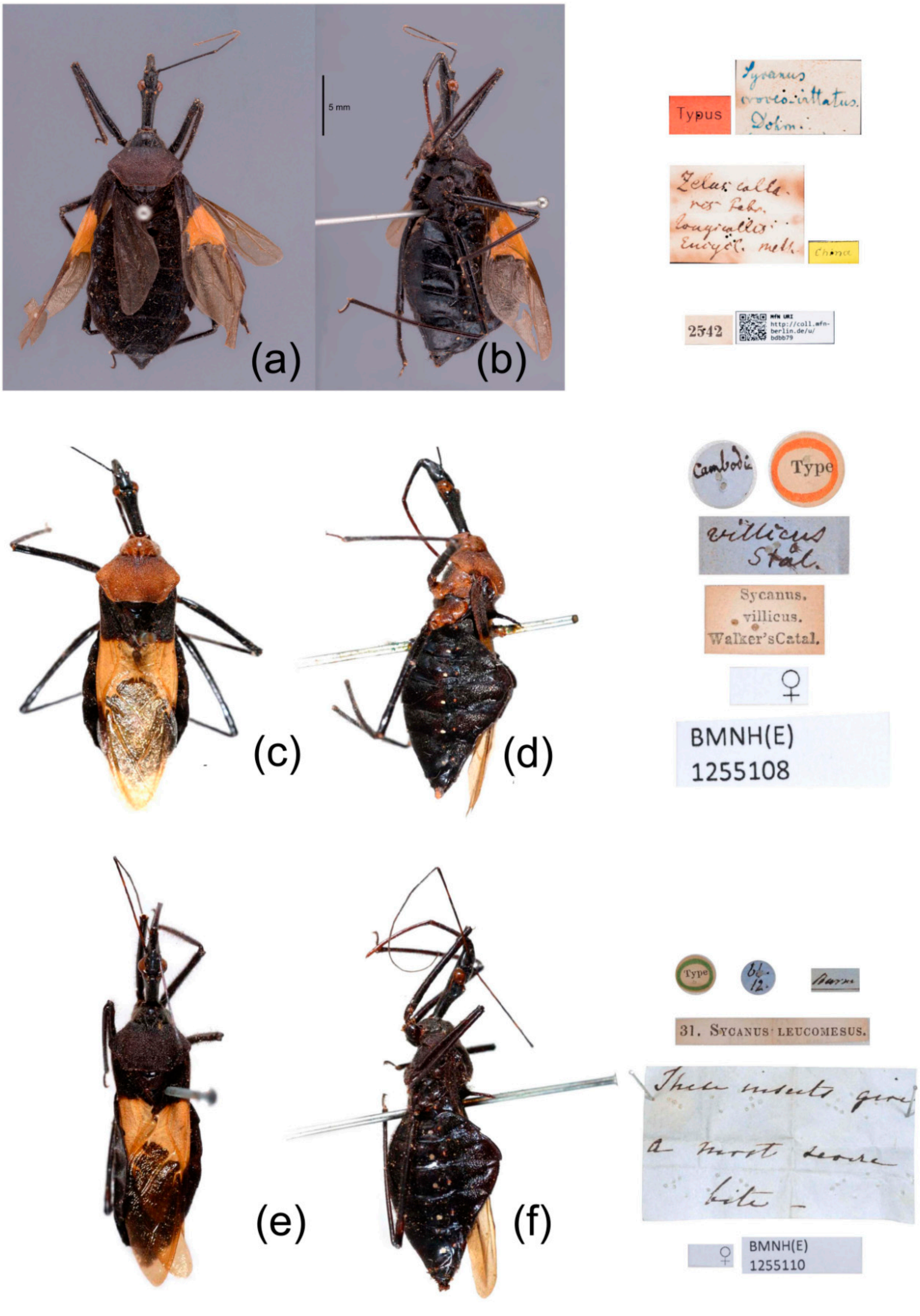

Figure S3. (a, b) *Sycanus croceovittatus* Dohrn, 1859, **Syntype**, female, habitus, deposited in MfN; (c, d), *Sycanus villicus* Stål, 1863, **Holotype**, female, habitus, deposited in BMNH, URL: https://data.nhm.ac.uk/object/9ac6a020-304f-4c2d-9cd5-7c0def47b4aa; (e, f), *Sycanus leucomesus* Walker, 1873, **Holotype**, female, habitus, deposited in BMNH, URL: https://data.nhm.ac.uk/object/456b420e-6818-4b48-88c6-fa861fa18d76; (a, c, e), dorsal view; (b, d, f), lateral view.

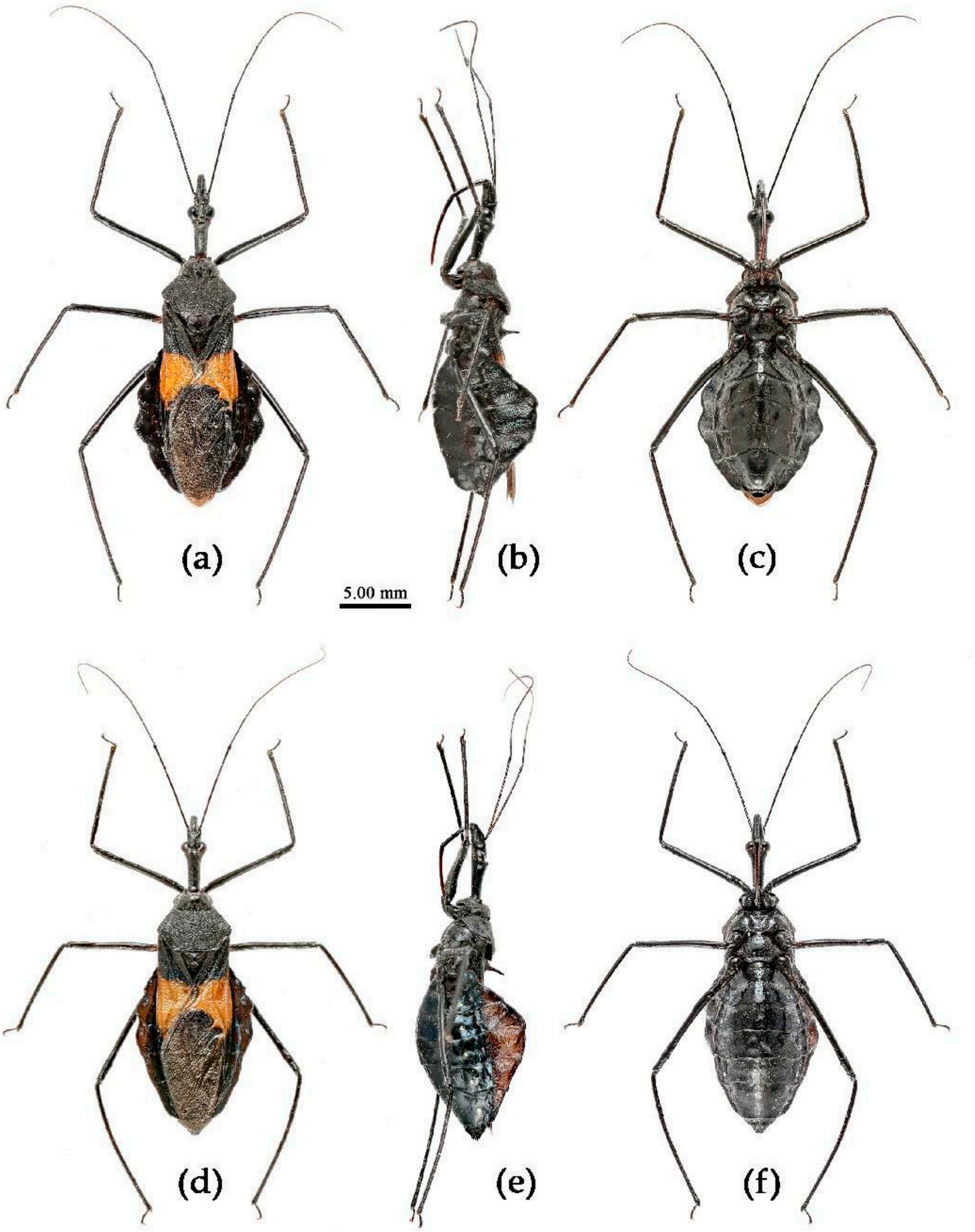

Figure S4. *Sycanus bifidus* (Fabricius, 1787), habitus, (a–c), male, (d–f), female, from Guangxi, China, deposited in CAU. (a, d), dorsal view; (b, e), lateral view; (c, f), ventral view.

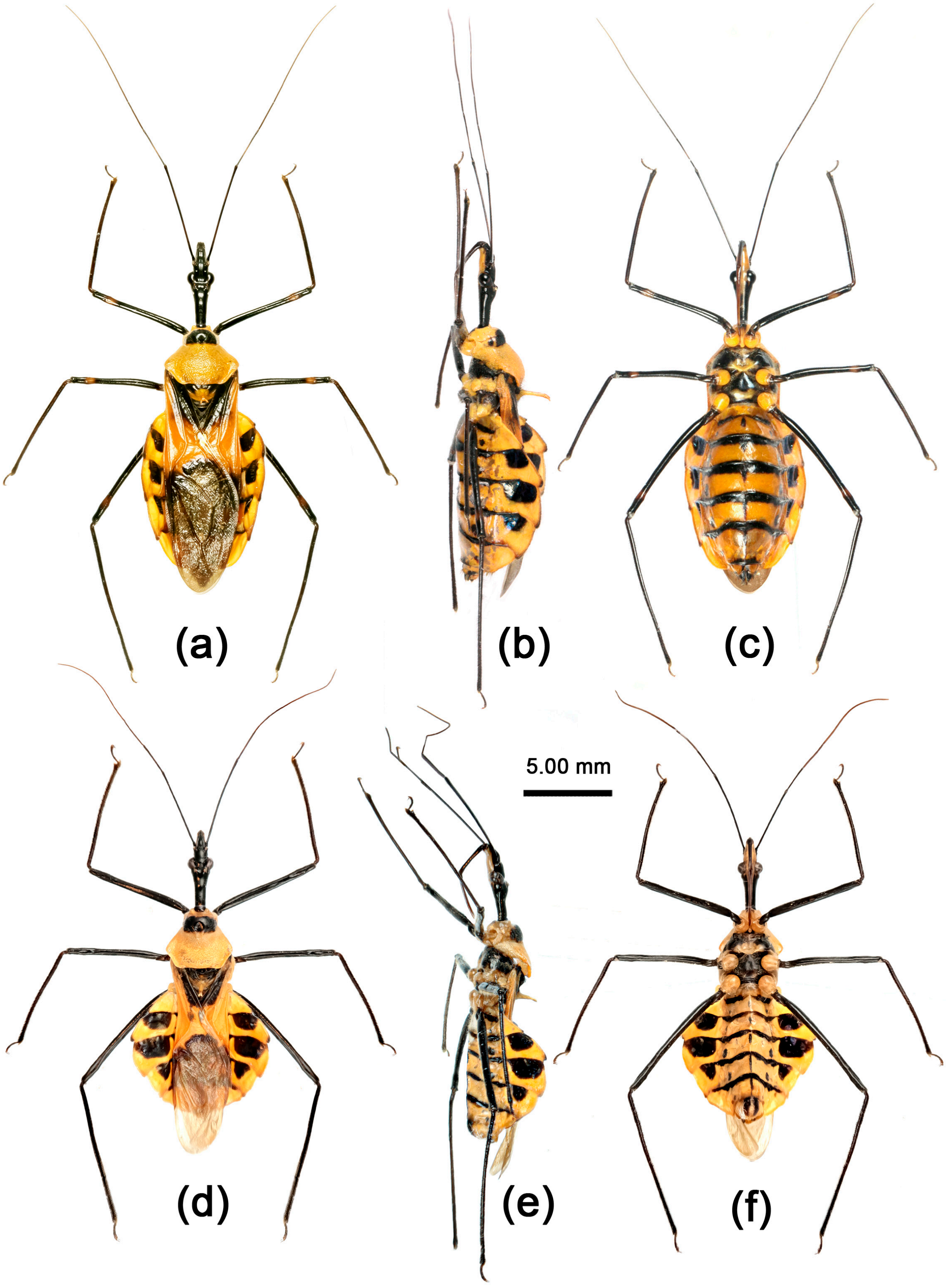

Figure S5. *Sycanus croceus* Hsiao, 1979, habitus, (a–c), male, (d–f), female, from Guangxi, China, deposited in CAU. (a, d), dorsal view; (b, e), lateral view; (c, f), ventral view.

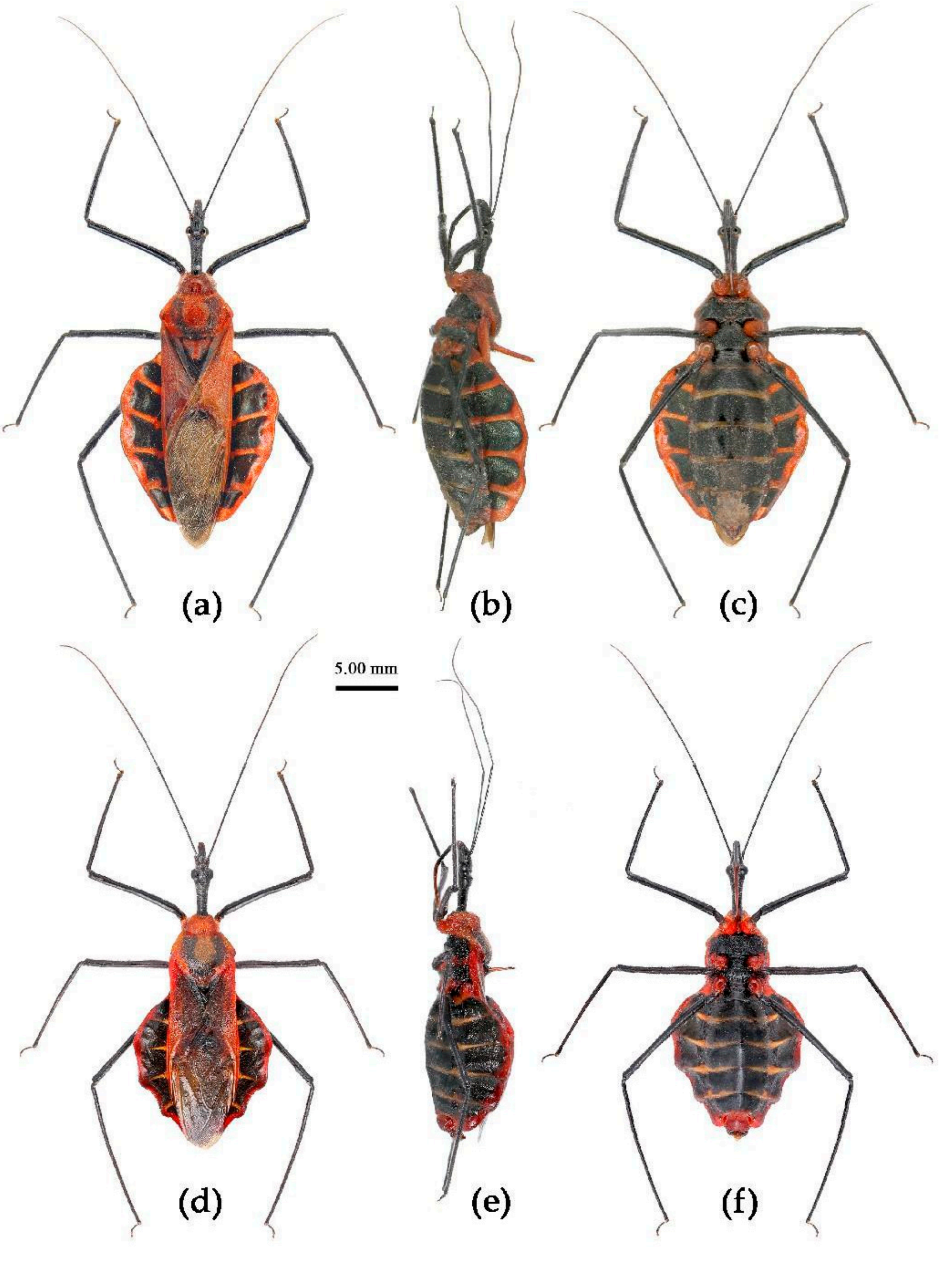

Figure S6. *Sycanus falleni* Stål, 1863, habitus, (a–c), female; (d–f), male, from Guangxi, China, deposited in CAU. (a, d), dorsal view; (b, e), lateral view; (c, f), ventral view.

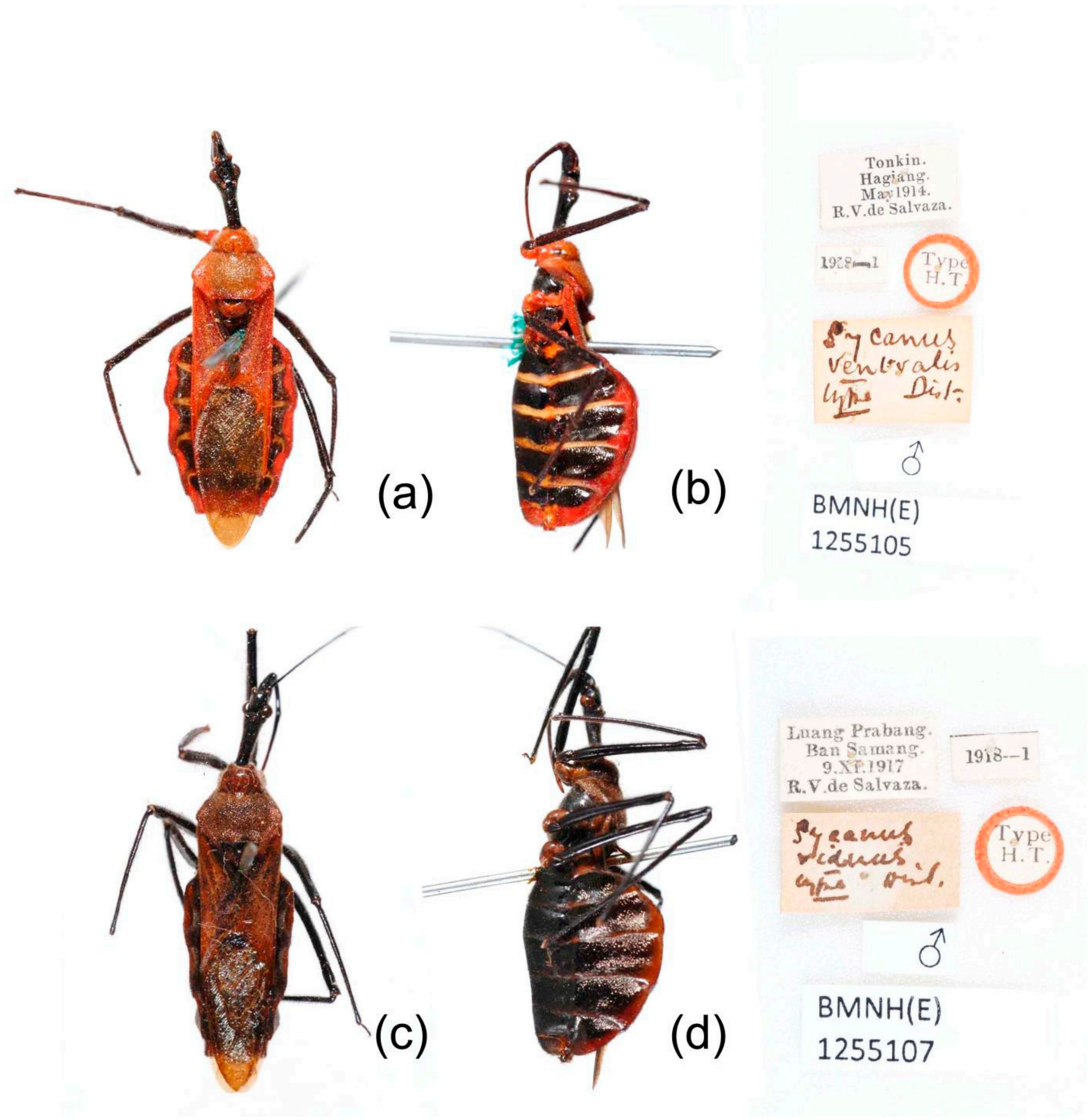

Figure S7. (a, b), *Sycanus ventralis* Distant, 1919, **Holotype**, male, habitus, deposited in BMNH, London, URL: https://data.nhm.ac.uk/object/fd802d93-58b1-4c1e-a72c-49e6cae36788; (c, d), *Sycanus viduus* Distant, 1919, **Holotype**, male, habitus, deposited in BMNH, London, URL: https://data.nhm.ac.uk/object/9ac6a020-304f-4c2d-9cd5-7c0def47b4aa. (a, c) dorsal view; (b, d) lateral view.

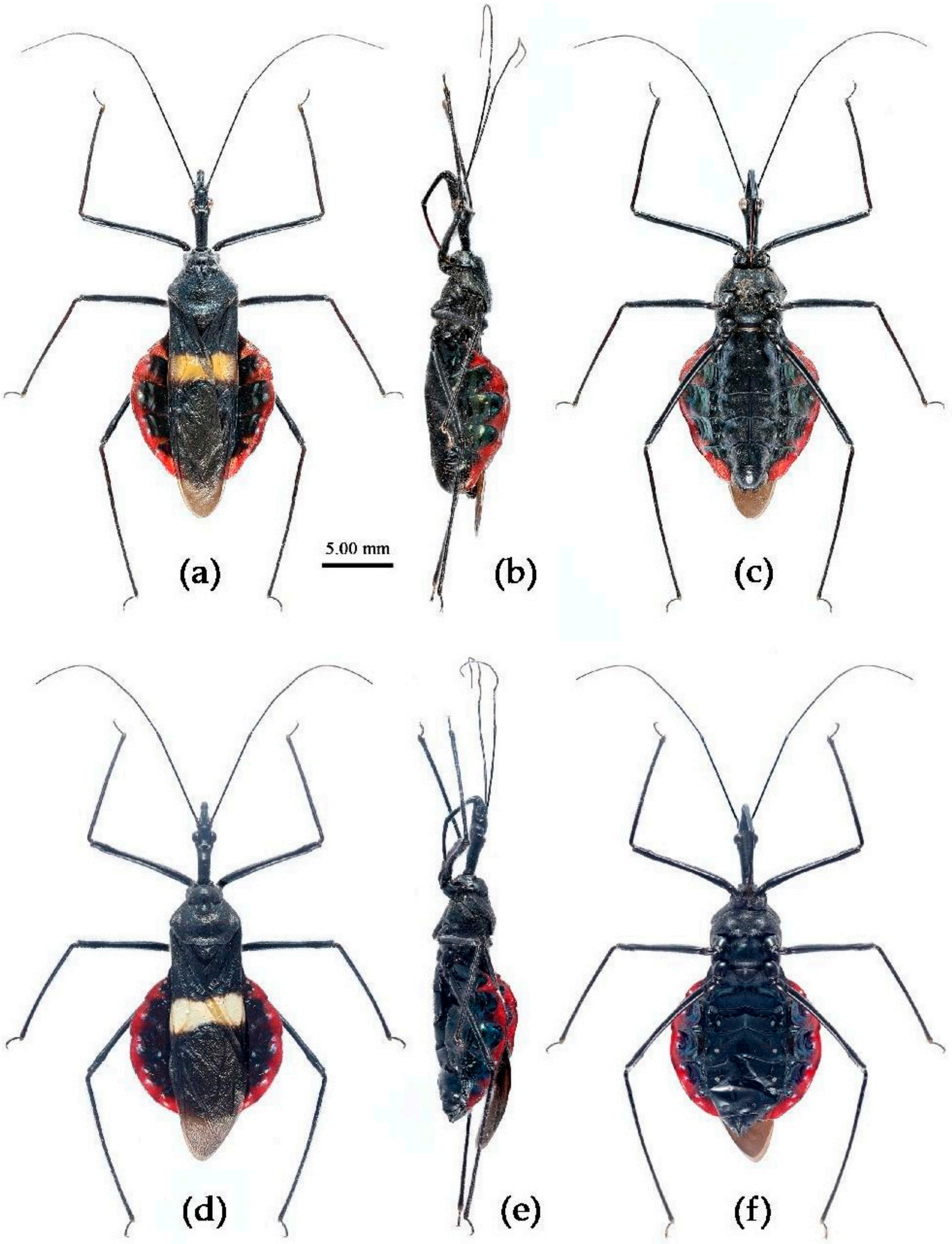

Figure S8. *Sycanus marginellus* Putshkov, 1987, habitus, (a–c), female, (d–f), male. (a, d), dorsal view; (b, e), lateral view; (c, f), ventral view.

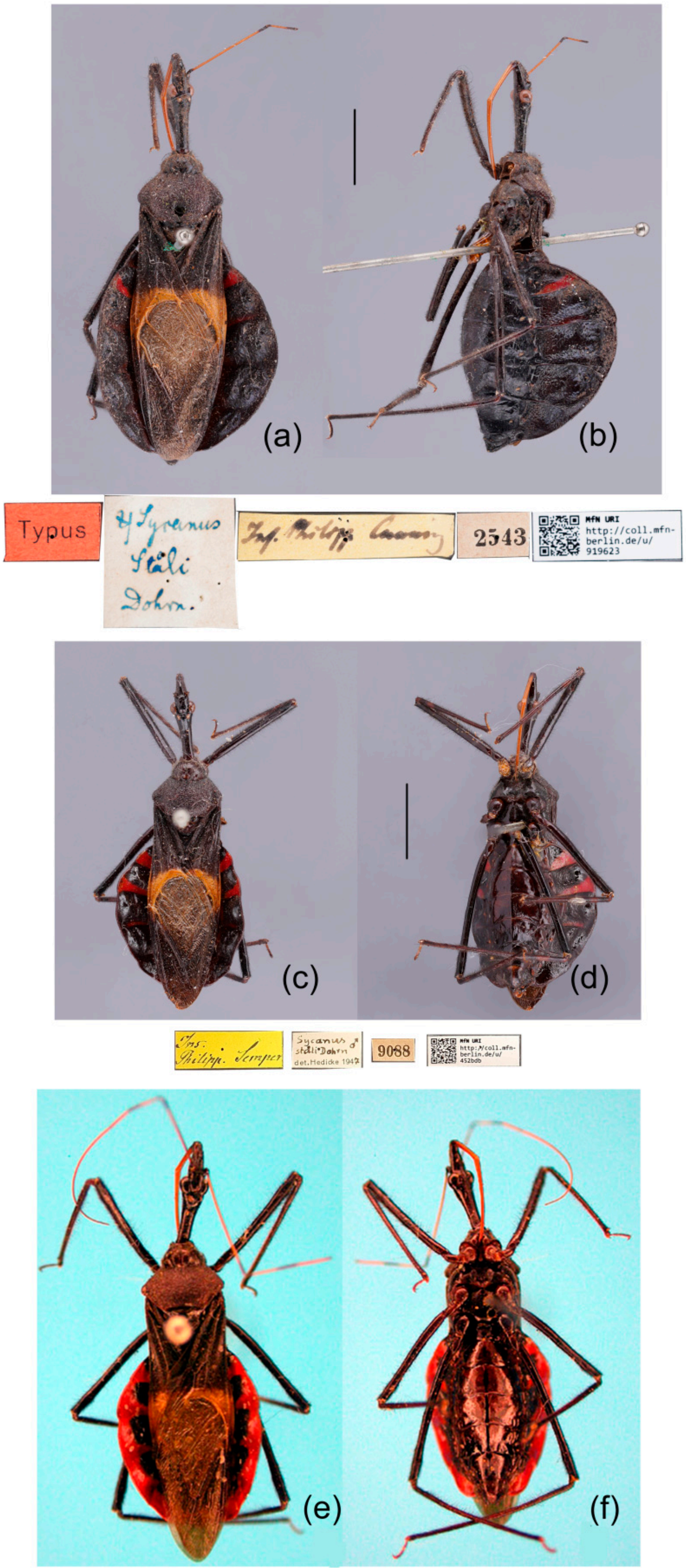

Figure S9. (a, b), *Sycanus stali* Dohrn, 1859, **Holotype**, female, habitus, deposited in MfN; (c, d), *Sycanus stali* Dohrn, 1859, **Paratype**, male, habitus, deposited in MfN; (e, f), *S. generosus* Stål, 1863, **Syntype**, male, habitus, deposited in NRM, URL: http://www2.nrm.se/en/het_nrm/g/sycanus_generosus.html.

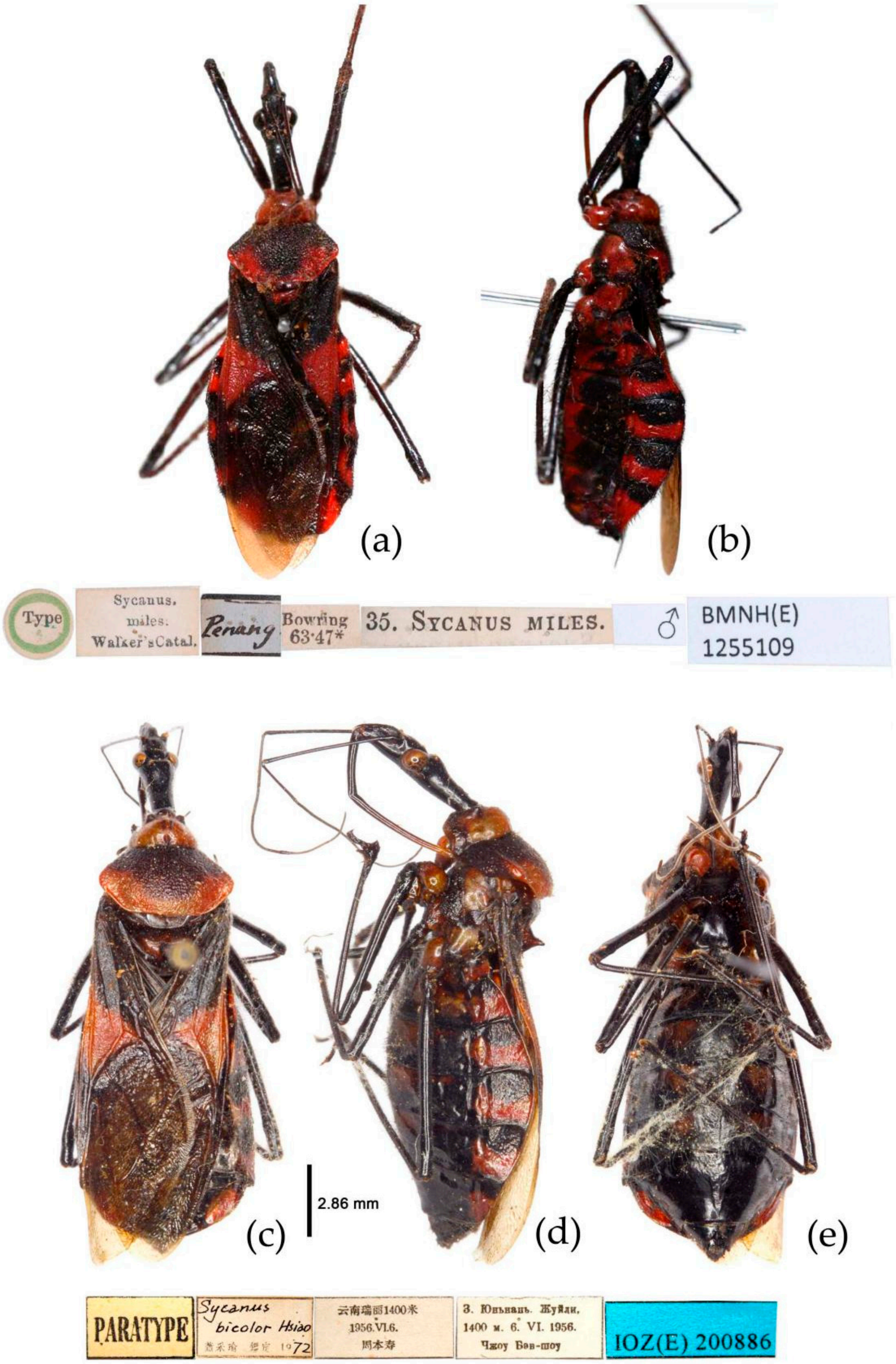

Figure S10. (a, b), *Sycanus miles* Walker, 1873, **Holotype**, deposited in BMNH, female, habitus, URL: https://data.nhm.ac.uk/object/836db954-c751-4d1c-8f45-bfde377bea1c; (c–e), *Sycanus bicolor* Hsiao, 1979, **Paratype**, deposited in IOZ, female, habitus; (a, c), dorsal view; (b, d), lateral view; (e), ventral view.

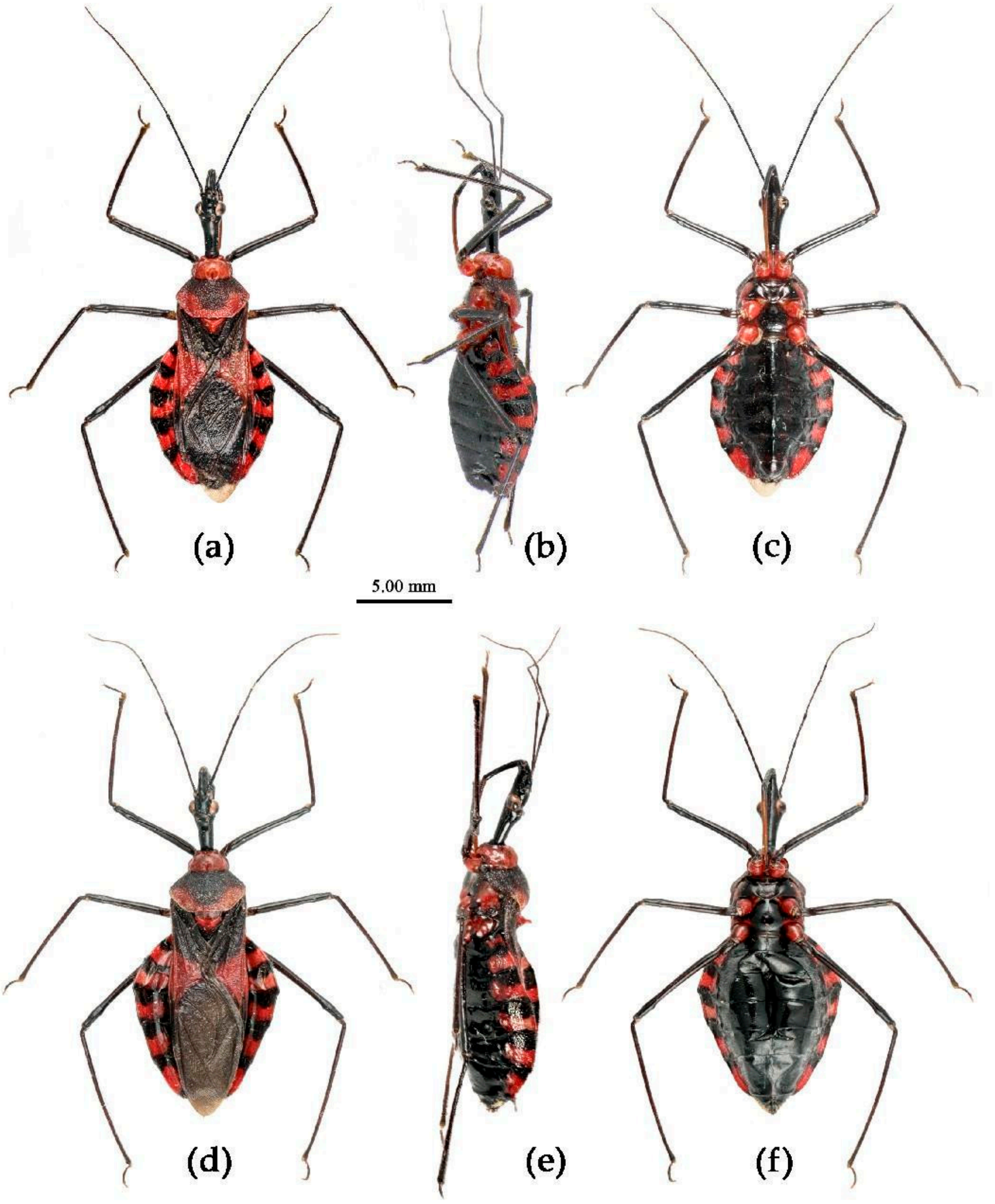

Figure S11. *Sycanus versicolor* Dohrn, 1859, habitus, (a–c), male, (d–f), female, from Yunnan, China. (a, d), dorsal view; (b, e), lateral view; (c, f), ventral view.

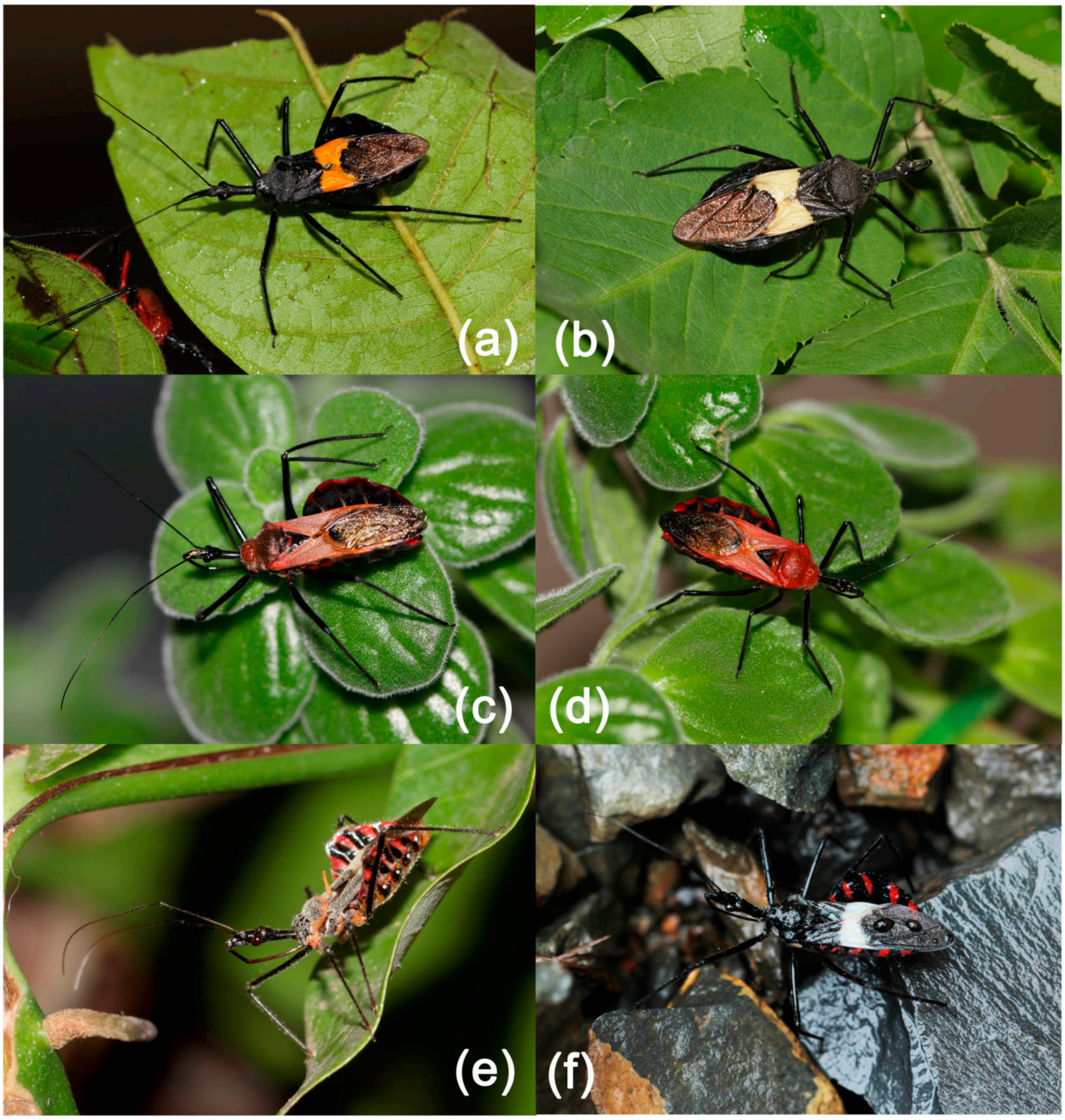

Figure S12. (a, b) *Sycanus bifidus* (Fabricius, 1787), from Guangxi, China; (c, b) *Sycanus falleni* Stål, 1863, Yunnan, China; (e) *Sycanus minor* Hsiao, 1979, from Guangxi, China; (f) *Sycanus sichuanensis* Hsiao, 1979, from Guizhou, China.

Table S1. GenBank Accession number under every sample individual code (in PDF file of Supporting information)

COI DNA barcodes

No.	Sample code	GenBank Accession number	No.	Sample code	GenBank Accession number
1*	MS-HN-COI	OP927069	44	HDX-LC5-COI	OP927112
2*	B-LC1-COI	OP927070	45	HDX-LC6-COI	OP927113
3*	DQ-3-COI	OP927071	46	HOX-YN2-COI	OP927114
4*	DQ-COI	OP927072	47	HOX-YN3-COI	OP927115
5*	B-LC2-COI	OP927073	48	HOX-YN4-COI	OP927116
6	DHX-10-COI	OP927074	49	HUX-1-COI-F	OP927117
7	DHX-11-COI	OP927075	50	HUX-2-COI-F	OP927118
8	DHX-13-COI	OP927076	51	HUX-3-COI-F	OP927119
9	DHX-GX1-COI	OP927077	52	HUX-4-COI-F	OP927120
10	DHX-GX2-COI	OP927078	53	HUX-5-COI-F	OP927121
11	ESX-1-2-COI	OP927079	54	HUX-6-COI-F	OP927122
12	ESX-1-COI	OP927080	55	HUX-7-COI-F	OP927123
13	ESX-YN1-COI	OP927081	56	HUX-FJ1-COI	OP927124
14	ESX-YN2-COI	OP927082	57	HUX-FJ2-COI	OP927125
15	ESX-YN3-COI	OP927083	58	HUX-FJ3-COI	OP927126
16	HBX-HaN1-COI	OP927084	59	SCX-11-COI	OP927127
17	HBX-HaN2-COI	OP927085	60	SCX-13-2-COI	OP927128
18	HBX-HaN3-COI	OP927086	61	SCX-1-COI	OP927129
19	HDX-10-HD2-COI	OP927087	62	SCX-5-COI	OP927130
20	HDX-11-HD3-COI	OP927088	63	SCX-8-COI	OP927131
21	HDX-12-COI	OP927089	64	SCX-GX1-COI	OP927132
22	HDX-12-HD4-COI	OP927090	65	SCX-GX3-COI	OP927133
23	HDX-13-COI	OP927091	66	SCX-GZ1-COI	OP927134
24	HDX-13-HD5-COI	OP927092	67	SCX-SC1	OP927135

25	HDX-14-HD6-COI	OP927093	68	SCX-SC2	OP927136
26	HDX-15-HD7-COI	OP927094	69	XX-HC-YN2-COI	OP927137
27	HDX-16-COI	OP927095	70	XX-HC-YN3-COI	OP927138
28	HDX-16-HD8-COI	OP927096	71	XX-YN10	OP927139
29	HDX-17-HD-COI	OP927097	72	XX-YN1-COI	OP927140
30	HDX-1-COI	OP927098	73	XX-YN3-COI	OP927141
31	HDX-2-COI	OP927099	74	XX-YN4	OP927142
32	HDX-3-COI	OP927100	75	XX-YN5	OP927143
33	HDX-5-COI	OP927101	76	XX-YN9	OP927144
34	HDX-6-COI	OP927102	77	XZ1-TW1-COI	OP927145
35	HDX-8-COI	OP927103	78	XZ1-TW2-COI	OP927146
36	HDX-COI	OP927104	79	XZ1-TW3-COI	OP927147
37	HDX-GD1-COI	OP927105	80	XZ2-HaN1-COI	OP927148
38	HDX-GD2-COI	OP927106	81	XZ2-HaN2-COI	OP927149
39	HDX-GD3-COI	OP927107	82	XZ3-YN1-COI	OP927150
40	HDX-LC1-COI	OP927108	83	XZ3-YN2-COI	OP927151
41	HDX-LC2-COI	OP927109	84	XZ3-YN3-COI	OP927152
42	HDX-LC3-COI	OP927110	85	ZYX-LC9-COI	OP927153
43	HDX-LC4-COI	OP927111	86	ZYX-YN1-COI	OP927154
			87*	HJQ-LC-COI	not submitted

Note. The outgroups are marked with an asterisk in the serial number.

Table S4. Life history of *Sycanus croceus* Hsiao, 1979 (China, Guangxi, Ningming)

	January			February			March			April			May			June			July			August			September			October			November			December		
	A	B	C	A	B	C	A	B	C	A	B	C	A	B	C	A	B	C	A	B	C	A	B	C	A	B	C	A	B	C	A	B	C	A	B	C
Egg																0	0	0	0	0	0	0	0													
1st instar nymph																		1	1	1	1	1	1	1	1											
2nd instar nymph																				2	2	2	2	2	2	2	2									
3rd instar nymph																						3	3	3	3	3	3	3	3							
4th instar nymph																									4	4	4	4	4	4	4	4				
5th instar nymph																												5	5	5	5	5	5	5		
Adult	(+)	(+)	(+)	(+)	(+)	(+)	(+)	(+)	(+)	(+)	(+)	(+)	+	+	+	+	+	+	+	+	+	+						+	+	+	(+)	(+)	(+)	(+)	(+)	(+)

†A: the first 10 days of month; B: the middle 10 days of month; C: the last 10 days of month. ‡ "0": egg; "1" : 1st instar nymph; "2" : 2nd instar nymph; "3" : 3rd instar nymph; "4": 4th instar of nymph; "5": 5th instar of nymph.

§“(+)”: overwinter adult; “+”: adult.

Table S5. Life history of *Sycanus falleni* Stål, 1863 (China, Guangxi, Ningming)

	January			February			March			April			May			June			July			August			September			October			November			December		
	A	B	C	A	B	C	A	B	C	A	B	C	A	B	C	A	B	C	A	B	C	A	B	C	A	B	C	A	B	C	A	B	C	A	B	C
Egg																0	0	0	0	0	0	0	0	0	0											
1st instar nymph																		1	1	1	1	1	1	1	1	1	1									
2nd instar nymph																			2	2	2	2	2	2	2	2	2	2								
3rd instar nymph																					3	3	3	3	3	3	3	3	3							
4th instar nymph																								4	4	4	4	4	4	4	4	4				
5th instar nymph																											5	5	5	5	5	5	5	5	5	5
Adult	(+)	(+)	(+)	(+)	(+)	(+)	(+)	(+)	(+)	(+)	(+)	(+)	(+)	+	+	+	+	+	+	+	+	+	+											(+)	(+)	(+)

†A: the first 10 days of month; B: the middle 10 days of month; C: the last 10 days of month. ‡ “0”: egg; “1” : 1st instar nymph; “2” : 2nd instar nymph; “3” : 3rd instar nymph; “4”: 4th instar of nymph; “5”: 5th instar of nymph.

§"(+)": overwinter adult; "+": adult.
